# The Kinetics and Mechanism of Niche Filling in Healthy and Cystic Mice

**DOI:** 10.34067/KID.0000001217

**Published:** 2026-05-07

**Authors:** Alex Yashchenko, Sarah J. Miller, Audrey M. Cordova, Morgan E. Yashchenko, Shuja Khan, Bibi Maryam, Isabella G. Darby, Yohan Park, Fariha Nusrat, Ummey Khalecha Bintha Ahmed, Kurt A. Zimmerman

**Affiliations:** Division of Nephrology, Department of Internal Medicine, University of Oklahoma Health Campus, Oklahoma City, Oklahoma

**Keywords:** immunology, macrophages, polycystic kidney disease

## Abstract

**Key Points:**

- The rate and mechanism of kidney-resident macrophage niche filling differs between healthy and cystic mice.
- An incompletely filled kidney-resident macrophage niche is associated with worsened polycystic kidney disease outcomes.

**Background:**

Previous data indicate that bone marrow–derived Ly6c^hi^ monocytes can become tissue-resident macrophages when the niche is available. However, the balance between monocyte recruitment and tissue-resident macrophage self-proliferation following partial depletion is unclear.

**Methods:**

We investigated the kinetics and mechanism of kidney-resident macrophage (KRM) niche filling in healthy mice and in a mouse model of autosomal dominant polycystic kidney disease after partial depletion with liposomal clodronate.

**Results:**

Our data indicate that healthy mice efficiently refilled their KRM niche through monocyte-dependent recruitment, engraftment, and differentiation while cystic mice relied preferentially on *in situ* KRM self-proliferation for niche filling. Furthermore, we found sex-specific differences in niche-filling capacity in cystic mice as female, but not male, mice were unable to completely fill their niche 90 days postdepletion. The inability of cystic female mice to fill the niche following depletion was likely due to their reduced ability to recruit monocytes and convert them into KRM. Phenotypically, delayed KRM niche filling was associated with worsened outcomes including increased cystic index and kidney fibrosis as well as worsened kidney function.

**Conclusions:**

Collectively, our data demonstrate that sex and disease status affect the kinetics and mechanism of niche filling in the kidney.

## Visual Abstract

**Figure:**
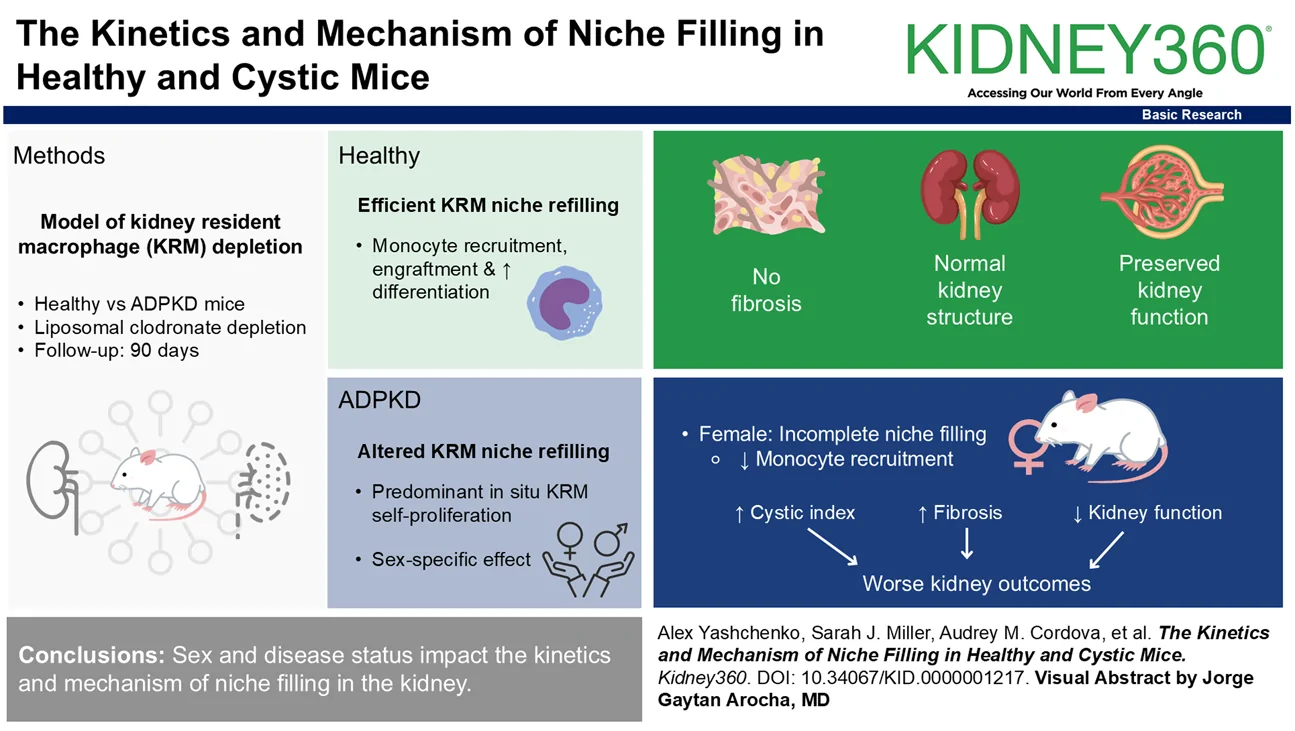

## Introduction

Recent studies indicate that tissue-resident macrophage (TRM) phenotype is dictated by ontogeny, time in tissue, localization, and the surrounding microenvironment.^1^ These factors dictate TRM function in the liver, pancreas, arteries, and central nervous system during homeostasis and disease^2–10^ and likely affect other TRM populations throughout the body. Previous literature indicates that kidney resident macrophages (KRMs) are embryonically seeded,^11^ with yolk sac-derived and fetal liver monocyte-derived macrophages filling the KRM niche during development.^11–13^ Upon birth, the healthy kidney undergoes moderate levels of monocyte recruitment,^14^ resulting in approximately 30% of KRM which are of monocyte origin by 12 weeks of age.^15,16^ Additional studies suggest that KRM ontogeny,^14^ localization,^16,17^ time spent in tissue,^18,19^ and the surrounding microenvironment^20^ may affect KRM function in health and disease.

Niche filling is the study of how TRM are restored following depletion resulting from pharmacologic or genetic means. Previous data in the healthy liver indicate that upon TRM (Kupffer cell [KC]) ablation, the KC niche is rapidly repopulated by monocyte recruitment, engraftment, and differentiation into KCs.^21^ Moreover, the acquisition of a KC-like phenotype was dependent on signaling between hepatic stellate and endothelial cells,^6^ suggesting the critical role of the microenvironment in driving niche filling. Similarly, in the healthy kidney, the KRM niche undergoes rapid bone marrow–derived monocyte infiltration and near-complete niche filling a week after depletion.^14,22^ The authors noted that functional phenotypes of KRM differed based on ontogeny, with embryonically seeded KRM displaying increased phagocytosis, production of proinflammatory cytokines, and a more active metabolic state as compared with bone marrow monocyte–derived KRM.^14^

KRM are associated with autosomal dominant polycystic kidney disease (ADPKD) as these cells increase in number and accumulate around cysts in animal models of the disease.^23–26^ KRM are thought to promote disease progression as chronic ablation of KRM and other phagocytes by liposomal clodronate (LC) decreases cyst number and cyst size.^23,27,28^ Furthermore, specific KRM ablation *via* GW2580, a colony stimulating factor-1 receptor kinase inhibitor, also slows polycystic kidney disease (PKD) progression in mouse models of the disease.^23,29^ Data from ADPKD patients suggest that KRM found in cystic regions of biopsied tissues are alternatively activated and express CD163.^24^ The presence of an alternatively activated (M2 like) KRM signature has been validated through single-cell RNA sequencing studies, where *C1qa*^30^ was identified as a shared, cross-species KRM marker enriched in mouse^31^ and human PKD samples.^32^ Follow-up studies from our laboratory similarly show that a subset of KRM, defined by *C1qa* and *Mrc1* expression, is present in cystic regions of mice and humans.^29,33^

The cystic microenvironment is thought to mediate KRM accumulation by producing chemokines such as macrophage migratory inhibitory factor, monocyte chemoattractant protein-1 ( C-C motif chemokine ligand 2), and colony stimulating factor 1 (CSF1). Inhibition of migratory inhibitory factor reduces pericystic KRM accumulation and delays cyst growth,^34^ whereas loss of *Mcp1* reduces monocyte infiltration into the kidney, delaying cystogenesis.^19^ Epithelial production of membrane-bound CSF1 is enriched upon cilia loss, and inhibiting CSF1-colony stimulating factor-1 receptor signaling reduces KRM proliferation and cyst progression.^29^ Moreover, the cystic microenvironment appears to dictate KRM phenotype in a time-dependent manner, with recruited monocytes first differentiating into M1 like macrophages that accumulate around cysts.^19^ After a week, most of the M1 like macrophages are replaced by M2 like macrophages, which promote fibrosis and drive rapid cyst growth.^19^ Overall, these studies suggest that the cystic microenvironment perturbs KRM phenotype, accumulation, and localization.

Sex-dimorphic differences in ADPKD are established in the literature, with male patients experiencing earlier onset of kidney growth and a more rapid rate of disease development which correlates with earlier onset of hypertension, cardiovascular complications, and kidney failure.^35,36^ However, these effects are complicated by factors such as the identity of the disease-causing mutation, with female *PKD2* patients experiencing better outcomes than male *PKD2* patients, but no sex-based differences are evident in *PKD1* patients.^37^ The severity of the mutation is another factor, as patients with truncating *PKD1* mutations develop ESKD earlier than either males or females with nontruncating *PKD1* or *PKD2* mutations.^38^ In orthologous *Pkd1* and *Pkd2* models of PKD, adult male mice experience more rapid disease progression than female mice,^39,40^ while in the hypomorphic *Pkd1*^RC/RC^ model, no sex differences are present until mice are 9-month-old, upon which point females experience worsened disease outcomes.^41^ Studies indicate that sex-dimorphic effects can be altered by manipulating the gonads; orchiectomized male PKD rats display reduced disease severity compared with intact controls, while testosterone supplemented male or ovariectomized female rats display increased disease severity.^42,43^

To study the role of the cystic microenvironment in regulating KRM niche filling, we performed a one-time, partial KRM depletion in a monocyte fate-mapping mouse model.^15^ We contrasted healthy and cystic mice on a sex-dimorphic level and observed differences in the rate and mechanism of niche filling. Surprisingly, our findings indicate that incomplete KRM niche filling correlates with worsened PKD outcomes, contrasting with previously published literature where chronic KRM depletion was associated with better PKD outcomes.

## Methods

### Mice

*Ms4a3*^Cre/Cre^ and *Pkd1*^RC/RC^ mice were gifted by Dr. Florent Ginhoux and Dr. Peter Harris, respectively. *Rosa*^TdT/TdT^ mice (B6.Cg-Gt[ROSA]26Sortm14[chicken beta-actin promoter-tdTomato]Hze/J; Stock No: 007914) were purchased from Jackson Laboratories. Mice were interbred to generate healthy (*Ms4a3*^Cre^ *Rosa*^TdT^) and cystic (*Ms4a3*^Cre^ *Rosa*^TdT^ *Pkd1*^RC/RC^) fate-mapping cohorts. The *Ms4a3*^Cre^ *Rosa*^TdT^ model labels approximately 90%–95% of monocyte-derived cells *via* tdTomato (TdT) expression that is turned on in granulocyte-monocyte progenitors.^15^ Specifically, upon Cre activation in these cells, the Rosa stop^f/f^ TdT reporter is permanently switched on, fluorescently labeling bone marrow–derived monocytes and their progeny. The *Pkd1*^RC/RC^ model is an orthologous model of PKD, carrying the R3277C mutation that leads to reduced polycystin-1 dosage and slowly progressive PKD.^44,45^ Animals were housed in Association for Assessment and Accreditation of Laboratory Animal Care-accredited facilities in accordance with Institutional Animal Care and Use Committee regulations at the University of Oklahoma Health Campus. Procedures were approved under protocol 23-002-SECHIX.

### Macrophage Depletion and Niche Filling

Five milligram per milliliter LC and liposomal vehicle (LV) were purchased from Liposoma B.V. Three-month–old mice received a single intraperitoneal injection of LC or LV at a dose of 50 mg/kg. KRM niche filling was analyzed on D3, D10, D22, D42, and D90 after depletion. These time points were chosen because of varied outcomes in KRM niche filling—near-complete KRM niche filling by D7 in a rapid genetic depletion model,^14,22^ and no KRM niche filling in a chronic LC depletion model.^23^ We performed a one-time, partial KRM depletion *via* LC and chose D22, D42, and D90 as prospective time points of complete KRM niche filling.

### Flow Cytometry

Following 1× Dulbecco's phosphate-buffered saline (DPBS) perfusion, kidneys and livers were harvested, minced, and digested (Kidneys: 1 ml Roswell Park Memorial Institute Medium (RPMI) 1640 medium containing 1 mg/ml collagenase type 1 and 100 U/ml deoxyribonuclease I; 30 minutes at 37°C. Livers: 5 ml RPMI in 1 mg/ml collagenase type 4 and 100 U/ml deoxyribonuclease I; 30 minutes at 37°C). Blood was collected from the right atrium into EDTA-coated tubes. Cell suspensions were passed through 70 *µ*m filters, centrifuged at 330 relative centrifugal force (RCF) for 5 minutes, and then ammonium-chloride-potassium red blood cell lysed for 5 minutes at room temperature. Ammonium-chloride-potassium lysis was stopped by adding 10 ml of RPMI. Cells were centrifuged at 300 RCF, resuspended in 1 ml of 1:200 1% BSA-containing Fc-blocking solution, and incubated for 30 minutes on ice. Following cell counting with trypan blue, approximately two million kidney, one million liver, and 300,000 blood cells were stained for 30 minutes at room temperature with primary antibodies (Supplemental Table 23). Cells were washed with 1% BSA, centrifuged at 300 RCF, and fixed in 5% formalin for 30 minutes on ice. Cells were washed with 1% BSA, centrifuged at 300 RCF, resuspended in 1× DPBS, and stored overnight at 4°C. Cells were analyzed on a Cytek Aurora 5L, and data were processed using FlowJo v10.10. A full list of reagents used in this study is provided in Supplemental Table 24.

### Histology and Kidney Function

Right kidneys were bisected along the midsagittal plane. One kidney half was immersion fixed in 10% formalin overnight at 4°C and then paraffin-embedded and sectioned (5 *µ*m). Kidney sections were stained with Harris-modified hematoxylin and eosin solution or picrosirius red. Cystic and fibrotic index, cystic area, cyst count, and average cyst size were quantified using Fiji/ImageJ. Cystic area, cyst count, and average cyst size were calculated using measure particles in Fiji/ImageJ. BUN was measured *via* a urea assay kit (Supplemental Table 24).

### Immunofluorescence and Image Analysis

The second kidney half was quartered—one-quarter was stored in −80°C; the second overnight-fixed in 10% formalin, cryoprotected in 30% sucrose, embedded in OCT and sectioned (6 *µ*m). Sections were permeabilized with 0.2% Triton X-100, and incubated in blocking solution (0.1% Triton X-100 in 1× DPBS, 1% w/v BSA, 1% v/v donkey serum, and 0.02% sodium azide) for 30 minutes at room temperature. Sections were incubated in primary antibody overnight at 4°C, washed with 1× DPBS, and incubated in secondary antibody for 1 hour at room temperature. The primary antibody was rat anti-mouse F4/80 (Thermo Fisher, Cat. No. 14-4801-82, Clone: BM8, 1:200). The secondary antibody was AF647-conjugated donkey anti-rat polyclonal antibody (Jackson ImmunoResearch, Cat. No. 712-606-153, Lot: 148033, 1:500). Nuclei were counterstained with Hoechst 33258 (Invitrogen, Cat. No. H3569, 1:1000) and samples mounted using Immu-Mount. Images were captured on a Nikon CSU-W1 SoRa confocal microscope.

Quantification was performed *via* Imaris Microscopy Image Analysis Software 10.2 using automated object detection. Channels were binarized using the Otsu method to calculate: (*1*) macrophage fraction—F4/80+/total nuclei, (*2*) TdT+ macrophage fraction—TdT+F4/80+/TdT+, (*3*) Jaccard/Dice indices—spatial overlap, and (*4*) pericystic macrophage fraction within a 25-*µ*m band defined by creating a surface object (cyst lining boundary) and selecting a region of interest (neighboring cells) within that boundary.

### Quantitative Reverse Transcription-PCR

RNA was isolated from frozen kidney quarters using TRIzol Plus RNA Purification Kit (Thermo Fisher). cDNA was synthesized using GoScript Reverse Transcriptase Kit (Promega) and PCR performed *via* TaqMan Gene Expression Master Mix (Thermo Fisher) on a QuantStudio 5 system using primers found in Supplemental Table 25. Data were normalized to *Hprt* using the comparative Ct method.

### Statistical Analyses

Data were analyzed using GraphPad Prism 10.6.1. For graphs comparing three independent variables, a three-way ANOVA followed by Benjamini–Krieger–Yekutieli *post hoc* test was used. A two-way ANOVA and Tukey *post hoc* test was used for graphs with two independent variables, while a one-way ANOVA with Tukey *post hoc* test was used for graphs with one independent variable. For graphs involving two groups with one independent variable, significance was determined using a Welch *t* test.

## Results

### Healthy and Cystic Mice Have Distinct KRM Niche-Filling Dynamics

To examine genotype- and sex-based differences during niche filling, we harvested mouse cohorts at sequential time points after LC injection (*e.g*., 3 days after depletion, D3; 10 days after depletion, D10) and quantified the abundance of KRM, Ly6c^hi^ infiltrating monocytes (Ly6c^hi^ IMs), Ly6c^hi^ monocyte-derived (TdT+) KRM, and proliferating (Ki67+ labeled) KRM by flow cytometry (Figure 1A). Three tissues were examined: (*1*) blood, as validation of the fate mapping model; (*2*) liver, because of a high degree of Ly6c^hi^ monocyte input after liver resident macrophage (KC) depletion^21^; and (*3*) kidney, the primary tissue of interest (Figure 1A). Immune cell abundance at each time point was compared with noninjected 3-month-old sex-matched littermate controls. The gating strategy used to identify blood, liver, and kidney immune cells is shown in Supplemental Figure 1, A–C.

**Figure 1:**
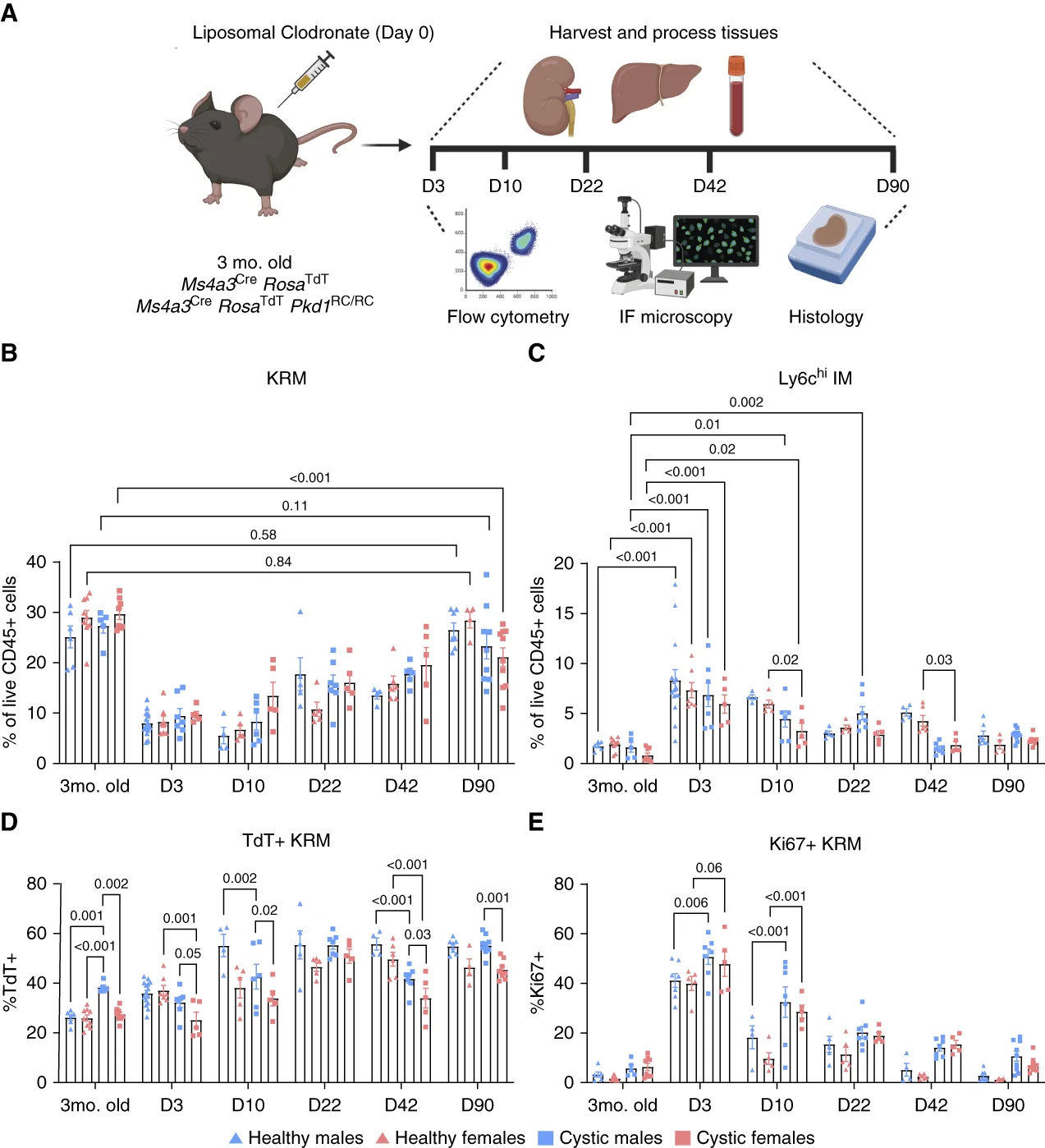
**Healthy and cystic mice possess distinct KRM niche filling kinetics.** (A) Schematic of the depletion and harvest strategy used to study TRM niche filling in healthy and cystic mice. (B) Quantification of KRM abundance by flow cytometry as a percentage of live CD45^+^ cells. (C) Quantification of kidney Ly6c^hi^ IM abundance by flow cytometry as a percentage of live CD45^+^ cells. (D) TdT labeling in KRM, signifying Ly6c^hi^ monocyte-derived KRM, expressed as a percentage of TdT+ KRM over all KRM in the kidney. (E) Ki67 expression in KRM, signifying proliferating KRM, expressed as a percentage of Ki67+ KRM over all KRM in the kidney. Statistics: three-way ANOVA with Benjamini–Krieger–Yekutieli *post hoc* multiple comparisons test. IF, immunofluorescence; KRM, kidney resident macrophage; Ly6c^hi^ IM, Ly6c^hi^ infiltrating monocyte; TdT, tdTomato; TRM, tissue-resident macrophage.

We first validated our fate-mapping model by analyzing TdT labeling in blood Ly6c^hi^ monocytes from healthy *Ms4a3*^Cre^ *Rosa*^TdT^ mice. Our data indicate that approximately 90% of Ly6c^hi^ monocytes are TdT+ in noninjected 3-month–old healthy male and healthy female mice, in agreement with previous data^15^ (Supplemental Figure 2A). We next turned to the liver and confirmed efficient LC-mediated KC depletion in healthy male and female mice on D3 (Supplemental Figure 2B). Additionally, we confirmed significant Ly6c^hi^ IM recruitment to the liver (Supplemental Figure 2C), as well as increased TdT labeling in KCs D3 postdepletion (Supplemental Figure 2D), in agreement with previous data.^15,21^ Altogether, these data indicate that the *Ms4a3*^Cre^ *Rosa*^TdT^ model labels the majority of Ly6c^hi^ monocytes in the blood and confirms previously published work showing significant monocyte input into the KC niche following depletion.^21^

A three-way ANOVA revealed a significant time×genotype interaction (*P* = 0.003) for KRM niche filling (Supplemental Table 1), indicating distinct repopulation patterns between healthy and cystic mice. Peak KRM depletion occurred 10 days after LC injection (*i.e*., D10) in both genotypes (Figure 1B and Supplemental Table 2). Healthy male and female mice showed similar rates of KRM niche filling over the span of the examined time points, leading to refilled KRM niches by D90 (*P* = 0.58 and *P* = 0.84, respectively; Figure 1B and Supplemental Table 2). On the contrary, while cystic male mice experienced complete KRM niche filling by D90 (*P* = 0.11), cystic female mice did not (*P* < 0.001; Figure 1B and Supplemental Table 2). To confirm complete KRM niche filling in healthy male, cystic male, and healthy female mice, we compared D90 LC-injected mice to D90 LV-injected mice as well as 3-month–old nondepleted controls (Supplemental Figure 3A). Our data indicate that these three groups of mice possessed refilled KRM niches by D90 (Supplemental Figure 3A and Supplemental Tables 3–5). On the contrary, LC-injected cystic female mice had significantly reduced KRM niche filling when compared with 3-month–old nondepleted or D90 LV-injected cystic female controls (*P* = 0.04; *P* = 0.04; Supplemental Figure 3A and Supplemental Table 6), further suggesting the presence of a KRM niche filling deficit in cystic female mice. We also checked whether CD11c+ dendritic cells (DCs), the major DC subtype in the murine kidney,^46,47^ were enriched following KRM depletion. Our data suggest an expansion of this cell type in healthy male and female mice by D10 (*P* < 0.001; Supplemental Figure 3B and Supplemental Tables 7 and 8), with the expanded DCs still present by D90 in both groups (healthy males: *P* < 0.001; healthy females: *P* = 0.001). On the contrary, although cystic male and female mice experienced an initial expansion of CD11c+ DCs on D3 and D10 (*P* < 0.001; *P* = 0.01), the DC population contracted back to baseline levels by D90 in cystic male mice (*P* = 0.47) and dropped below baseline levels in cystic female mice (*P* = 0.03; Supplemental Figure 3B and Supplemental Tables 7 and 8), suggesting that cystic female mice do not experience sustained compensatory niche filling from CD11c+ DCs.

We next quantified Ly6c^hi^ IM abundance in the kidney, which also showed a significant time×genotype interaction (*P* = 0.006; Supplemental Table 9). Ly6c^hi^ IM abundance was increased on D3 in all groups of mice compared with 3-month–old nondepleted controls (*P* < 0.001; Figure 1C and Supplemental Table 10). We also found that healthy female mice displayed increased amounts of Ly6c^hi^ IMs at D10 and D42 compared with their cystic counterparts (*P* = 0.02; *P* = 0.03; Figure 1C and Supplemental Table 10), suggesting that healthy female mice are more efficient at continuously recruiting monocytes to refill the open niche. When comparing cystic male to cystic female mice, we did not find significant differences in Ly6c^hi^ IM abundance at any of the time points; however, we noted that cystic females only had enriched Ly6c^hi^ IM abundance at D3 and D10 (*P* < 0.001; *P* = 0.02), whereas cystic males had enriched Ly6c^hi^ IM abundance at D3, D10, and D22 when compared with 3-month–old nondepleted controls (*P* < 0.001; *P* = 0.01; *P* = 0.002; Figure 1C and Supplemental Table 10). To further investigate the propensity of Ly6c^hi^ monocytes to engraft into the cystic kidney, we quantified the expression of the key chemokine driving this process, monocyte chemoattractant protein-1 (*Ccl2*). Our data suggest that during the active niche filling period (D22), LC-injected cystic male mice have significantly enriched *Ccl2* levels compared with LV-injected controls (*P* = 0.03; Supplemental Figure 4A). No difference was seen in cystic female mice (*P* = 0.60), and no sex-dimorphic effects were present (Supplemental Figure 4B).

The capacity of Ly6c^hi^ IMs to differentiate into KRM was measured by analyzing the proportion of KRM with TdT labeling following depletion. In line with previous work,^15^ approximately 25%–30% of KRM were TdT+ in 3-month–old nondepleted healthy male, healthy female, and cystic female mice (Figure 1D). However, 3-month–old nondepleted cystic male mice had a significantly increased percentage of TdT+ KRM (approximately 40%) compared with other controls (Figure 1D and Supplemental Table 12). During niche filling (time×genotype interaction, *P* < 0.0001; Supplemental Table 11), healthy male and female mice had increased percentage of TdT+ KRM at multiple time points compared with their cystic counterparts (D3 females: *P* = 0.001; D10 males: *P* = 0.002; D42 females: *P* < 0.001; D42 males: *P* < 0.001; Figure 1D and Supplemental Table 12). Additionally, cystic female mice exhibited significantly reduced rates of Ly6c^hi^ IM-to-KRM differentiation compared with cystic male mice (Figure 1D). Specifically, the percentage of TdT+ KRM was reduced in cystic females when compared with cystic males on D3, D10, D42, and D90 (*P* = 0.05; *P* = 0.02; *P* = 0.03; *P* = 0.001, respectively; Figure 1D and Supplemental Table 12). We also quantified the expression of fractalkine (*Cx3cl1*), a driver of monocyte-to-KRM differentiation, and of *Csf1*, a key cytokine regulating KRM maintenance. When compared with LV-injected controls, we saw no difference in *Cx3cl1* expression among LC-injected cystic male or female mice (*P* = 0.08; *P* = 0.78), but noted an increase in *Csf1* expression in cystic males (*P* = 0.04; Supplemental Figure 4A). No significant sex-dimorphic differences were seen for either factor (Supplemental Figure 4B).

Finally, we examined the proliferative capacity of KRM by quantifying the percentage of Ki67+ labeled cells by flow cytometry. Once again, an overall analysis of Ki67+ labeling over the niche-filling period indicated a significant time×genotype interaction (*P* = 0.02; Supplemental Table 13), indicating that genotype (*i.e*., disease status) was a significant driver of the observed differences in KRM self-renewal over time. Cystic mice of both sexes had greater Ki67+ labeling compared with their healthy counterparts at multiple time points (D3 males: *P* = 0.006; D3 females: *P* = 0.06; D10 males: *P* < 0.001; D10 females: *P* < 0.001; Figure 1E and Supplemental Table 14). We did not observe significant differences in the level of KRM proliferation between sexes within each genotype group (Figure 1E and Supplemental Table 14). In summary, we find that healthy mice preferentially recruit and engraft monocytes into the KRM niche, whereas cystic mice preferentially replenish their niche through KRM self-proliferation. We also find that cystic male mice undergo a sustained duration of increased monocyte recruitment, monocyte-to-KRM differentiation, and chemokine enrichment compared with cystic female mice, likely contributing to incomplete niche filling observed in cystic female mice.

### Cystic Female Mice Have Decreased TdT+ and Cyst-Localized Macrophage Abundance after Depletion

Previous data from our laboratory and others indicate that macrophages are cyst-localized in mouse models of PKD.^23–28^ To confirm our flow cytometry data and test whether depletion affects KRM localization after refilling, we performed confocal microscopy on kidney sections stained with the pan-macrophage marker F4/80 (Figure 2A). We first analyzed F4/80+ area over nuclear area in cystic male and female mice as we identified sex as an overall driver of variation in our data (*P* = 0.006; Supplemental Table 15). When compared with 3-month–old nondepleted controls, we observed nonsignificant trends toward reduced F4/80+ area in cystic male and cystic female mice on D10 (*P* = 0.24; *P* = 0.24; Figure 2B and Supplemental Table 16). Analysis of KRM abundance across sex also showed a nonsignificant trend toward KRM reduction in cystic females compared with cystic males on D22 and D90 (*P* = 0.05; *P* = 0.05; Figure 2B and Supplemental Table 16). We next quantified the percentage of TdT and F4/80 double positive cells over the niche-filling period (Figure 2C). With sex again identified as an overall driver of variation in our data (*P* = 0.003; Supplemental Table 17), we observed that 3-month–old nondepleted male mice had a nonsignificant trend toward an increased percentage of TdT+ KRM compared with 3-month–old nondepleted cystic female mice (*P* = 0.06; Figure 2C and Supplemental Table 18). This trend, although not significant, matched the previous increase in the percentage of TdT+ KRM by flow cytometry (Figure 1D and Supplemental Table 12). Starting on D10, cystic mice displayed a nonsignificant sex-based divergence in the percentage of TdT+ KRM (Figure 2C). This divergence grew over the niche-filling period, reaching near-significance on D42 (*P* = 0.07) and significance on D90 (*P* = 0.004), with cystic male mice at approximately 40% TdT+ KRM and cystic female mice at approximately 25% TdT+ KRM (Figure 2C and Supplemental Table 18).

**Figure 2:**
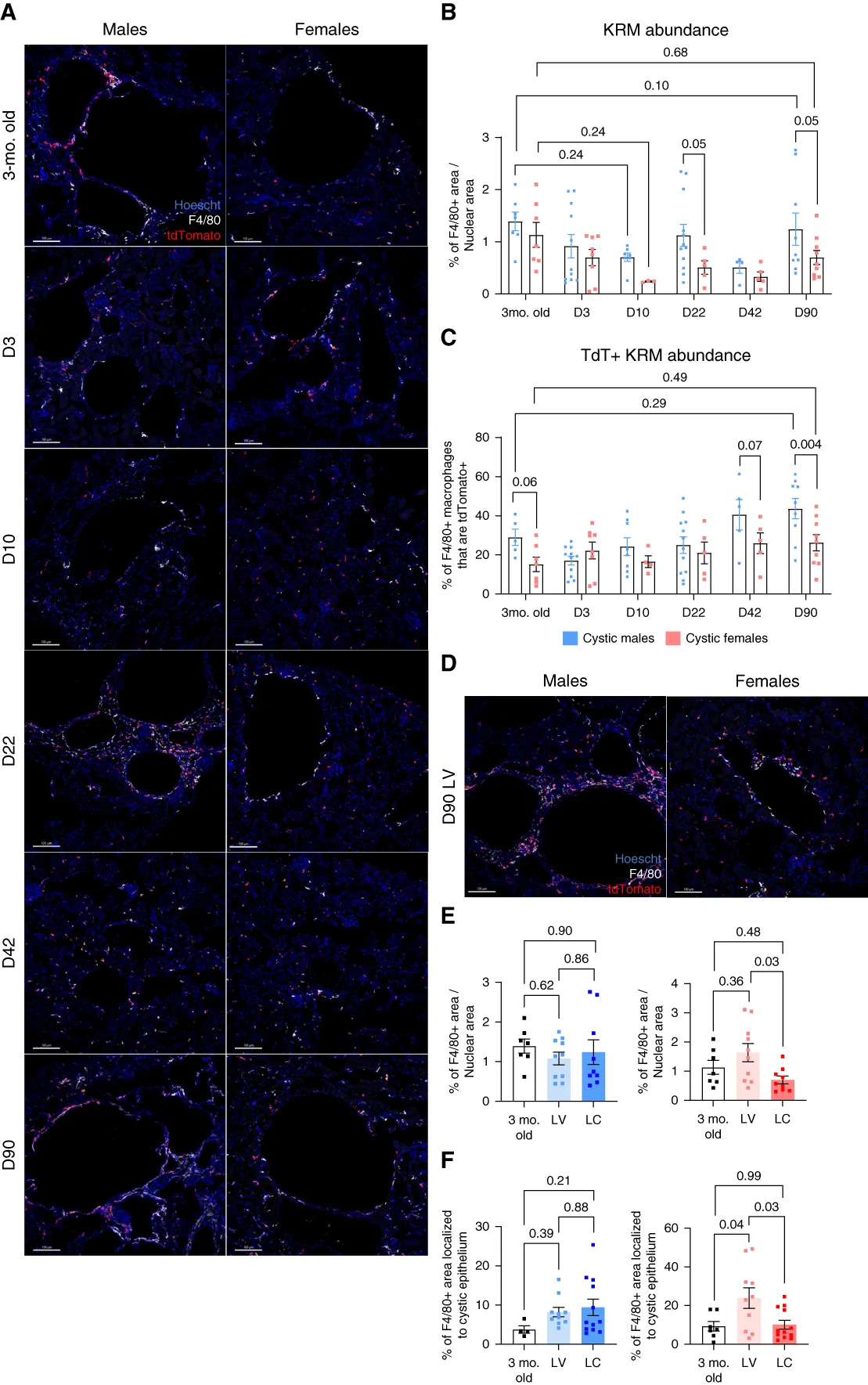
**Cystic female mice display a lower abundance of total KRM, TdT+ KRM, and cyst-localized KRM.** (A) Representative confocal microscopy images of kidneys harvested from male and female *Ms4a3*^Cre^ *Rosa*^TdT^ *Pkd1*^RC/RC^ mice during the niche filling period. Sections were stained with a pan-macrophage marker F4/80 and imaged with a 10× objective. (B) Quantification of total KRM abundance in cystic male and female mice during the niche filling period. (C) Quantification of TdT+ KRM abundance during the niche filling period in cystic male and cystic female mice. (D) Representative confocal microscopy images from male and female *Ms4a3*^Cre^ *Rosa*^TdT^ *Pkd1*^RC/RC^ mice harvested 90 days after LV administration. (E) Quantification of total KRM abundance in cystic male and female 3-month-old nondepleted controls, D90 LV, and D90 LC groups. (F) Quantification of cyst-localized KRM in cystic male and female 3-month-old nondepleted controls, D90 LV-injected controls, and D90 LC-depleted experimental mice. Statistics: (B and C) Two-way ANOVA with Tukey multiple comparisons test. (E and F) One-way ANOVA with Tukey multiple comparisons test. LC, liposomal clodronate; LV, liposomal vehicle.

To control for the expected progression of cystic disease and macrophage accumulation over the 90-day timeframe in the absence of macrophage depletion, we compared KRM abundance between LC- and LV-injected age-matched littermates (Figure 2, A and D, respectively). Our data confirm that LC cystic male mice have a filled KRM niche by D90 (*P* = 0.86) while LC cystic female mice do not (*P* = 0.03; Figure 2E and Supplemental Table 19). Finally, we quantified the abundance of F4/80+ cells associated with cysts in male and female mice D90 postdepletion. Our data indicate no significant difference in cyst-associated KRM abundance when comparing noninjected 3-month–old male mice with D90 LV- and LC-injected cystic male mice (Figure 2F and Supplemental Table 20). On the contrary, we observed a significant reduction in cyst-associated KRM abundance in LC-injected cystic female mice compared with LV-injected cystic female mice on D90 (*P* = 0.03; Figure 2F and Supplemental Table 20).

### Delayed KRM Niche Filling Is Associated with Worsened Cystic Kidney Disease

To test the impact of delayed niche filling on PKD progression, we quantified cystic index, fibrosis, and kidney function at the D90 time point in both sexes. For these analyses, we compared disease phenotypes between LV- and LC-injected age-matched littermates. There was no difference in two kidney weight to body weight ratio, cystic index, cyst count, or average cyst size between LC- and LV-injected cystic male mice at the D90 time point (Figure 3, A–C and Supplemental Table 21). By contrast, we found that LC-injected female mice had significantly increased two kidney weight to body weight and cystic index (*P* = 0.01; *P* = 0.004) and nonsignificantly increased cyst count and average cyst size (*P* = 0.09; *P* = 0.11) compared with LV-injected mice (Figure 3, A–C and Supplemental Table 21). We also found that female, but not male, cystic mice had increased picrosirius red-positive area (*P* = 0.03) and worsened kidney function as measured by BUN (*P* = 0.01) compared with LV-injected controls (Figure 3, D–F and Supplemental Table 22). Altogether, these data indicate that delayed niche filling in cystic female mice is associated with accelerated PKD progression.

**Figure 3:**
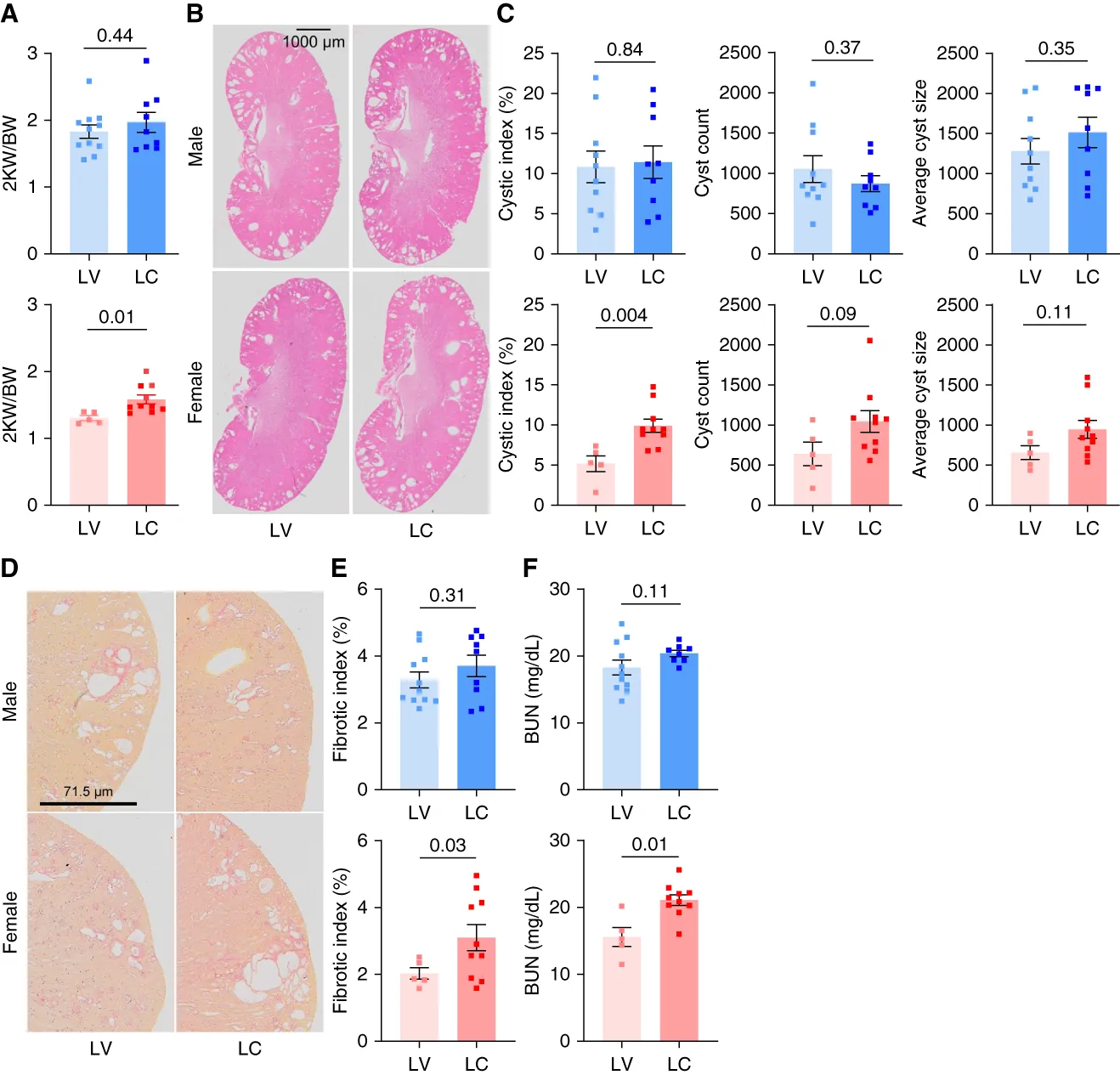
**An unfilled KRM niche is associated with worsened PKD and kidney function.** (A) 2KW/BW ratios from male and female *Ms4a3*^Cre^ *Rosa*^TdT^ *Pkd1*^RC/RC^ mice at the D90 time point. LV-injected mice are compared with LC-injected mice. Blue—cystic males, red—cystic females. (B) Representative H&E kidney images from D90 cystic male and female mice. (C) Quantification of disease phenotype from D90 cystic male and female mice. (D) Representative Picrosirius red images from D90 cystic male and female mice. (E) Quantification of fibrosis phenotype. (F) Quantification of BUN analysis. Statistics: Welch *t* test. H&E, hematoxylin and eosin; PKD, polycystic kidney disease; 2KW/BW, two kidney weight to body weight.

## Discussion

In this study, we investigated the process of KRM niche filling following a one-time, partial KRM depletion using a bone marrow-derived Ly6c^hi^ monocyte fate-mapping model. We found that healthy mice primarily rely on monocyte recruitment and differentiation to restore their KRM niche, whereas cystic mice preferentially replenish their niche through *in situ* KRM self-proliferation. We also found that cystic female, but not cystic male, mice were unable to completely fill their niche, which we attributed to a reduced capacity of female mice to recruit and differentiate monocytes into KRM. Surprisingly, delayed niche filling in cystic female mice was associated with worsened PKD outcomes including increased cystic index and worsened kidney function. Collectively, these data indicate that both biologic sex and disease status may heavily affect niche filling in the kidney.

One important finding in our study is the difference in the mechanism of niche filling between healthy and cystic mice. In healthy mice, the process is monocyte-driven, mirroring the niche-filling dynamics observed in previous studies of the homeostatic kidney, liver, and skin,^14,21,22,48^ suggesting that the healthy kidney microenvironment favors the recruitment and differentiation of circulating monocytes. Conversely, in cystic mice, niche filling appears to predominantly occur *via* a proliferation-driven mechanism. As we used the noninducible *Pkd1*^RC/RC^ model of PKD, this shift is likely dictated by differences in the cystic microenvironment of the tissue at the time of depletion. Multiple studies indicate enriched local production of growth factors and chemokines in PKD, such as C-C motif chemokine ligand 2 and CSF1.^19,29,34,49,50^ Throughout the niche-filling period, we observed significantly higher KRM proliferation by cystic mice compared with healthy mice (Figure 1E and Supplemental Table 14), supporting the idea that the cystic microenvironment may prime the KRM niche for self-renewal. Because the LC-mediated approach does not completely deplete the KRM niche (approximately 75% depletion), our approach allowed us to test the importance of both KRM self-proliferation and monocyte recruitment during the process of niche filling.

It is important to note that our one-time, partial KRM deletion *via* LC significantly differs from previous KRM niche filling studies using rapid, complete, and highly specific ablation of KRM.^14,22^ This difference between our study and previous data is stark. For example, previous studies using a CD59/intermedilysin approach to deplete KRM found that the KRM niche was approximately 85% recovered 7 days after depletion, with recovery primarily driven by monocyte recruitment.^14,22^ These data are similar to those reported by Scott and colleagues, which showed near-complete KC niche filling approximately 7 days postdiphtheria toxin-mediated KC depletion, driven once again predominantly by monocyte recruitment and differentiation.^21^ In contrast to these studies, our data indicate that the KRM niche in healthy mice is not refilled until approximately 90 days postdepletion. The difference in the kinetics of niche filling between this study and previous data is likely due to the slower mechanism of LC-mediated KRM apoptosis, with our data indicating maximum KRM reduction 10 days after depletion. Unfortunately, we cannot rule out indirect effects of LC, as literature indicates changes to tissue architecture, alteration of stromal cells, and a shift of hematopoietic stem cells toward myeloid differentiation.^51–53^ This suggests that while LC-mediated KRM depletion may trigger a compensatory expansion of myeloid progenitors, overall structural damage to the tissue may delay these cells from effectively filling the open niche. Additionally, as LC-mediated KRM depletion is not 100% effective, it is likely that residual KRM are allowed to self-proliferate to fill the niche, as we observe significant KRM self-proliferation even in healthy mice. Studying niche-filling dynamics in the context of partial KRM depletion may be necessary to fully understand what happens to the open TRM niche.

Another important finding is the inability of cystic female mice to fully refill their KRM niche. Although initial Ly6c^hi^ monocyte infiltration is similar across sexes, cystic males maintain a longer duration of enriched monocyte recruitment (Figure 1C and Supplemental Table 10) and have higher rates of monocyte-to-KRM differentiation (Figure 1D and Supplemental Table 12) compared with cystic females. We propose that the female cystic microenvironment is less permissive to the engraftment, differentiation, and successful integration of monocyte-derived cells into the KRM niche. This could be due to sex hormone-dependent differences in chemokine production or by differences in extracellular matrix remodeling, which provides the physical scaffold required for monocyte engraftment. We noted that on D22, cystic male, but not female, mice had an enrichment in *Ccl2* and *Csf1* expression (Supplemental Figure 4A), suggesting that lack of enriched chemokines could be driving incomplete niche filling in female mice. Previous studies indicate that vitronectin produced by KRM induces fibroblast activation and enriches Col1a1, *α*alpha-smooth muscle actin, and TGF-*β*1 levels in the kidney.^54^ As myofibroblast-epithelial-KRM cross talk has been proposed in PKD,^27,55,56^ it is possible that dysregulation of the cross talk under diminished KRM conditions leads to remodeled extracellular matrix, contributing to diminished monocyte engraftment and worsened PKD outcomes. Finally, we attempted to identify a compensatory cell type, such as DCs, which could be filling the KRM niche in cystic females, but did not observe their prolonged enrichment in PKD mice. Recent studies suggest that CD8^+^ T cells are protective in the *Pkd1*^RC/RC^ model of PKD.^57^ Although mechanisms driving T-cell activation in PKD are unknown, our data indicate cross talk between T cells and KRM,^31^ with additional preliminary studies in the *Ift88* model suggesting that T-cell–derived IFN*γ* can exacerbate PKD outcomes.^58^ Whether LC-induced shifts in myelopoiesis further polarize T-cell subtypes toward a more proinflammatory state, potentially contributing to the exacerbated damage seen in females, warrants further investigation.

The correlation between incomplete niche filling in cystic female mice and worsening PKD raises questions about the role of macrophages in this model of PKD. Previous data indicate that chronic KRM depletion improves PKD-associated outcomes in two rapidly progressing disease models.^23,29^ In our study, we find that a one-time, transient KRM reduction in the noninducible, slowly progressive disease model (*Pkd1*^RC/RC^) results in long-lasting macrophage depletion in cystic female mice, which is associated with worsened PKD outcomes. Our data suggest that macrophages present in the tissue at the time of depletion are likely disease-restricting, such that prolonged depletion of this population accelerates PKD progression. These results also suggest that phenotype and function of kidney macrophages may differ depending on the mouse model of PKD, with macrophages in rapidly progressing models being disease promoting and those in slowly progressing models being disease restricting. Future studies directly comparing macrophage phenotype and function in slowly and rapidly progressing models of PKD are warranted.

The finding that a one-time, partial KRM depletion accelerates PKD may carry implications for human therapeutic strategies, given that our findings are from an orthologous model of PKD. Our findings suggest that temporary KRM depletion may be risky: Temporarily disrupting the KRM niche may inadvertently remove a potentially protective KRM population and leave the kidney vulnerable to accelerated PKD. Our data suggest that KRM-targeted therapies in humans should aim for (*1*) identification and classification of KRM subsets based on disease-restricting or promoting phenotypes and (*2*) specific depletion of a disease-promoting KRM subset or enhancement of a disease-restricting cell type.

The primary limitation in our study is the lack of an experimental model that would strengthen the association between delayed KRM niche filling and accelerated PKD. As such, we cannot definitively rule out that worsened disease in cystic females may be due to factors that coincide with, but are not caused by, delayed KRM niche filling. Second, we lack mechanistic insights into the observed sex differences in cystic mice. Future studies examining hormone replacement therapy or ovariectomy surgeries would likely strengthen our correlative findings by conclusively teasing out sex-based differences among our cystic mice. Third, LC affects several types of mononuclear phagocytes, including monocytes and DCs, preventing cell type specificity that is achieved in transgenic models of depletion.

## Supplementary Material

## Data Availability

All original data, including deidentified patient-level data or individual laboratory data measurements, are included in the manuscript and/or supplemental material.

## Declarative Statements

All animal experiments were conducted in accordance with the NIH Guide for the Care and Use of Laboratory Animals or an equivalent standard that meets or exceeds the ethical and welfare requirements outlined in the NIH Guide. All protocols were approved by the appropriate Institutional Animal Care and Use Committee.

## Supplemental Material

Supplemental Table 1. Three-way ANOVA for Figure 1B.

Supplemental Table 2. Three-way ANOVA *post hoc* multiple comparisons test for Figure 1B.

Supplemental Table 3. One-way ANOVA of healthy male mice for Supplemental Figure 3A.

Supplemental Table 4. One-way ANOVA of healthy female mice for Supplemental Figure 3A.

Supplemental Table 5. One-way ANOVA of cystic male mice for Supplemental Figure 3A.

Supplemental Table 6. One-way ANOVA of cystic female mice for Supplemental Figure 3A.

Supplemental Table 7. Three-way ANOVA for Supplemental Figure 3B.

Supplemental Table 8. Three-way ANOVA *post hoc* multiple comparisons test for Supplemental Figure 3B.

Supplemental Table 9. Three-way ANOVA for Figure 1C.

Supplemental Table 10. Three-way ANOVA *post hoc* multiple comparisons test for Figure 1C.

Supplemental Table 11. Three-way ANOVA for Figure 1D.

Supplemental Table 12. Three-way ANOVA *post hoc* multiple comparisons test for Figure 1D.

Supplemental Table 13. Three-way ANOVA for Figure 1E.

Supplemental Table 14. Three-way ANOVA *post hoc* multiple comparisons test for Figure 1E.

Supplemental Table 15. Two-way ANOVA for Figure 2B.

Supplemental Table 16. Two-way ANOVA *post hoc* multiple comparisons test for Figure 2B.

Supplemental Table 17. Two-way ANOVA for Figure 2C.

Supplemental Table 18. Two-way ANOVA *post hoc* multiple comparisons test for Figure 2C.

Supplemental Table 19. One-way ANOVA and *post hoc* multiple comparisons test for Figure 2E.

Supplemental Table 20. One-way ANOVA and *post hoc* multiple comparisons test for Figure 2F.

Supplemental Table 21. Welch *t* tests for Figure 3, A–C.

Supplemental Table 22. Welch *t* tests for Figure 3, D–F.

Supplemental Table 23. Primary antibodies used for flow cytometry.

Supplemental Table 24. List of flow cytometry, staining, and immunofluorescence reagents.

Supplemental Table 25. Quantitative reverse transcription-PCR primer sequences.

Supplemental Figure 1. Flow cytometry gating strategy.

Supplemental Figure 2. Validation of the fate-mapping mouse model; blood and liver data.

Supplemental Figure 3. Validation of KRM niche filling and DC abundance.

Supplemental Figure 4. Chemokine comparison between cystic male and female mice.

## References

[1] Bleriot C Chakarov S Ginhoux F. Determinants of resident tissue macrophage identity and function. Immunity. 2020;52(6):957–970. doi:10.1016/j.immuni.2020.05.014 32553181

[2] Weinberger T Esfandyari D Messerer D, . Ontogeny of arterial macrophages defines their functions in homeostasis and inflammation. Nat Commun. 2020;11(1):4549. doi:10.1038/s41467-020-18287-x PMC7486394 32917889

[3] De Ponti FF Bujko A Liu Z, . Spatially restricted and ontogenically distinct hepatic macrophages are required for tissue repair. Immunity. 2025;58(2):362–380.e10. doi:10.1016/j.immuni.2025.01.002 39862865

[4] Zhu Y Herndon JM Sojka DK, . Tissue-resident macrophages in pancreatic ductal adenocarcinoma originate from embryonic hematopoiesis and promote tumor progression. Immunity. 2017;47(2):323–338.e6. doi:10.1016/j.immuni.2017.07.014 PMC5578409 28813661

[5] Soysa R Lampert S Yuen S, . Fetal origin confers radioresistance on liver macrophages via p21(cip1/waf1). J Hepatol. 2019;71(3):553–562. doi:10.1016/j.jhep.2019.04.015 PMC12509797 31077791

[6] Bonnardel J T'Jonck W Gaublomme D, . Stellate cells, hepatocytes, and endothelial cells imprint the Kupffer cell identity on monocytes colonizing the liver macrophage niche. Immunity. 2019;51(4):638–654.e9. doi:10.1016/j.immuni.2019.08.017 PMC6876284 31561945

[7] Ydens E Amann L Asselbergh B, . Profiling peripheral nerve macrophages reveals two macrophage subsets with distinct localization, transcriptome and response to injury. Nat Neurosci. 2020;23(5):676–689. doi:10.1038/s41593-020-0618-6 PMC7611025 32284604

[8] Lavin Y Winter D Blecher-Gonen R, . Tissue-resident macrophage enhancer landscapes are shaped by the local microenvironment. Cell. 2014;159(6):1312–1326. doi:10.1016/j.cell.2014.11.018 PMC4437213 25480296

[9] Silvin A Uderhardt S Piot C, . Dual ontogeny of disease-associated microglia and disease inflammatory macrophages in aging and neurodegeneration. Immunity. 2022;55(8):1448–1465.e6. doi:10.1016/j.immuni.2022.07.004 35931085

[10] Guilliams M Bonnardel J Haest B, . Spatial proteogenomics reveals distinct and evolutionarily conserved hepatic macrophage niches. Cell. 2022;185(2):379–396.e38. doi:10.1016/j.cell.2021.12.018 PMC8809252 35021063

[11] Hoeffel G Chen J Lavin Y, . C-myb(+) erythro-myeloid progenitor-derived fetal monocytes give rise to adult tissue-resident macrophages. Immunity. 2015;42(4):665–678. doi:10.1016/j.immuni.2015.03.011 PMC4545768 25902481

[12] Gomez Perdiguero E Klapproth K Schulz C, . Tissue-resident macrophages originate from yolk-sac-derived erythro-myeloid progenitors. Nature. 2015;518(7540):547–551. doi:10.1038/nature13989 PMC5997177 25470051

[13] Epelman S Lavine KJ Beaudin AE, . Embryonic and adult-derived resident cardiac macrophages are maintained through distinct mechanisms at steady state and during inflammation. Immunity. 2014;40(1):91–104. doi:10.1016/j.immuni.2013.11.019 PMC3923301 24439267

[14] Liu F Dai S Feng D, . Distinct fate, dynamics and niches of renal macrophages of bone marrow or embryonic origins. Nat Commun. 2020;11(1):2280. doi:10.1038/s41467-020-16158-z PMC7210253 32385245

[15] Liu Z Gu Y Chakarov S, . Fate mapping via ms4a3-expression history traces monocyte-derived cells. Cell. 2019;178(6):1509–1525.e19. doi:10.1016/j.cell.2019.08.009 31491389

[16] Yashchenko A Bland SJ Song CJ, . Cx3cr1 controls kidney resident macrophage heterogeneity. Front Immunol. 2023;14:1082078. doi:10.3389/fimmu.2023.1082078 PMC10225589 37256130

[17] Chew C Brand OJ Yamamura T, . Kidney resident macrophages have distinct subsets and multifunctional roles. Matrix Biol. 2024;127:23–37. doi:10.1016/j.matbio.2024.02.002 PMC7619362 38331051

[18] Puranik AS Leaf IA Jensen MA, . Kidney-resident macrophages promote a proangiogenic environment in the normal and chronically ischemic mouse kidney. Sci Rep. 2018;8(1):13948. doi:10.1038/s41598-018-31887-4 PMC6141464 30224726

[19] Cassini MF Kakade VR Kurtz E, . Mcp1 promotes macrophage-dependent cyst expansion in autosomal dominant polycystic kidney disease. J Am Soc Nephrol. 2018;29(10):2471–2481. doi:10.1681/ASN.2018050518 PMC6171277 30209078

[20] Cheung MD Erman EN Moore KH, . Resident macrophage subpopulations occupy distinct microenvironments in the kidney. JCI Insight. 2022;7(20):e161078. doi:10.1172/jci.insight.161078 PMC9714795 36066976

[21] Scott CL Zheng F De Baetselier P, . Bone marrow-derived monocytes give rise to self-renewing and fully differentiated Kupffer cells. Nat Commun. 2016;7:10321. doi:10.1038/ncomms10321 PMC4737801 26813785

[22] Islamuddin M Evangelista J Kabir ME Vidal AKN Liu S Qin X. Rapid depletion of renal macrophages using human cd59/intermedilysin cell ablation tool. J Vis Exp. 2025;(219):e67957. doi:10.3791/67957 40418671

[23] Karihaloo A Koraishy F Huen SC, . Macrophages promote cyst growth in polycystic kidney disease. J Am Soc Nephrol. 2011;22(10):1809–1814. doi:10.1681/ASN.2011010084 PMC3187181 21921140

[24] Swenson-Fields KI Vivian CJ Salah SM, . Macrophages promote polycystic kidney disease progression. Kidney Int. 2013;83(5):855–864. doi:10.1038/ki.2012.446 PMC4028685 23423256

[25] Karl R Ashraf APK Viola MF Hopp K Mass E Wachten D. Renal tissue-resident macrophages promote cystogenesis in early polycystic kidney disease. J Cell Sci. 2025;138(20):jcs263992. doi:10.1242/jcs.263992 PMC12450461 40814262

[26] Aloria EJG Song CJ Li Z, . Ly6c(hi) infiltrating macrophages promote cyst progression in injured conditional ift88 mice. Kidney360. 2021;2(6):989–995. doi:10.34067/KID.0000882021 PMC8359900 34396149

[27] Yang Y Chen M Zhou J, . Interactions between macrophages and cyst-lining epithelial cells promote kidney cyst growth in pkd1-deficient mice. J Am Soc Nephrol. 2018;29(9):2310–2325. doi:10.1681/ASN.2018010074 PMC6115670 30042193

[28] Li Z Hombal RP Wang J, . Macrophage accumulation and cyst expansion in pkd2, ift88, and double mutant mouse models. J Am Soc Nephrol. 2025;36(11):2131–2144. doi:10.1681/ASN.0000000746 PMC12591675 40455584

[29] Zimmerman KA Song CJ Li Z, . Tissue-resident macrophages promote renal cystic disease. J Am Soc Nephrol. 2019;30(10):1841–1856. doi:10.1681/ASN.2018080810 PMC6779366 31337691

[30] Zimmerman KA Bentley MR Lever JM, . Single-cell RNA sequencing identifies candidate renal resident macrophage gene expression signatures across species. J Am Soc Nephrol. 2019;30(5):767–781. doi:10.1681/ASN.2018090931 PMC6493978 30948627

[31] Song CJ Li Z Ahmed UKB, . A comprehensive immune cell atlas of cystic kidney disease reveals the involvement of adaptive immune cells in injury-mediated cyst progression in mice. J Am Soc Nephrol. 2022;33(4):747–768. doi:10.1681/ASN.2021030278 PMC8970461 35110364

[32] Liu F Li X Li Q, . Deciphering intercellular communication of the immune landscape within autosomal dominant polycystic kidney disease microenvironment at single-cell resolution. Kidney Dis (Basel). 2025;11(1):302–318. doi:10.1159/000545663 PMC12105836 40421435

[33] Li Z Zimmerman KA Cherakara S, . A kidney resident macrophage subset is a candidate biomarker for renal cystic disease in preclinical models. Dis Model Mech. 2023;16(1):dmm049810. doi:10.1242/dmm.049810 PMC9884121 36457161

[34] Chen L Zhou X Fan LX, . Macrophage migration inhibitory factor promotes cyst growth in polycystic kidney disease. J Clin Invest. 2015;125(6):2399–2412. doi:10.1172/JCI80467 PMC4497763 25961459

[35] Cornec-Le Gall E Audrezet MP Chen JM, . Type of pkd1 mutation influences renal outcome in ADPKD. J Am Soc Nephrol. 2013;24(6):1006–1013. doi:10.1681/ASN.2012070650 PMC3665389 23431072

[36] Bae KT Zhou W Shen C, . Growth pattern of kidney cyst number and volume in autosomal dominant polycystic kidney disease. Clin J Am Soc Nephrol. 2019;14(6):823–833. doi:10.2215/CJN.10360818 PMC6556721 31088850

[37] Hateboer N v Dijk MA Bogdanova N, . Comparison of phenotypes of polycystic kidney disease types 1 and 2. European pkd1-pkd2 study group. Lancet. 1999;353(9147):103–107. doi:10.1016/s0140-6736(98)03495-3 10023895

[38] Cornec-Le Gall E Audrezet MP Rousseau A, . The PROPKD score: a new algorithm to predict renal survival in autosomal dominant polycystic kidney disease. J Am Soc Nephrol. 2016;27(3):942–951. doi:10.1681/ASN.2015010016 PMC4769200 26150605

[39] Menezes LF Lin CC Zhou F Germino GG. Fatty acid oxidation is impaired in an orthologous mouse model of autosomal dominant polycystic kidney disease. EBioMedicine. 2016;5:183–192. doi:10.1016/j.ebiom.2016.01.027 PMC4816756 27077126

[40] Outeda P Summers P Basquin D, . Inactivation of pkd2 in adult mice results in delayed cyst formation and identifies sex as a major modifier of disease severity. Kidney Int. 2026;109(2):323–336. doi:10.1016/j.kint.2025.08.039 PMC13449052 41077129

[41] Arroyo J Escobar-Zarate D Wells HH, . The genetic background significantly impacts the severity of kidney cystic disease in the pkd1(rc/rc) mouse model of autosomal dominant polycystic kidney disease. Kidney Int. 2021;99(6):1392–1407. doi:10.1016/j.kint.2021.01.028 PMC9107297 33705824

[42] Cowley BD Jr. Rupp JC Muessel MJ Gattone VH II. Gender and the effect of gonadal hormones on the progression of inherited polycystic kidney disease in rats. Am J Kidney Dis. 1997;29(2):265–272. doi:10.1016/s0272-6386(97)90039-1 9016899

[43] Stringer KD Komers R Osman SA Oyama TT Lindsley JN Anderson S. Gender hormones and the progression of experimental polycystic kidney disease. Kidney Int. 2005;68(4):1729–1739. doi:10.1111/j.1523-1755.2005.00589.x 16164649

[44] Rossetti S Kubly VJ Consugar MB, . Incompletely penetrant pkd1 alleles suggest a role for gene dosage in cyst initiation in polycystic kidney disease. Kidney Int. 2009;75(8):848–855. doi:10.1038/ki.2008.686 PMC2813773 19165178

[45] Hopp K Ward CJ Hommerding CJ, . Functional polycystin-1 dosage governs autosomal dominant polycystic kidney disease severity. J Clin Invest. 2012;122(11):4257–4273. doi:10.1172/JCI64313 PMC3484456 23064367

[46] Kruger T Benke D Eitner F, . Identification and functional characterization of dendritic cells in the healthy murine kidney and in experimental glomerulonephritis. J Am Soc Nephrol. 2004;15(3):613–621. doi:10.1097/01.asn.0000114553.36258.91 14978163

[47] Tittel AP Heuser C Ohliger C Knolle PA Engel DR Kurts C. Kidney dendritic cells induce innate immunity against bacterial pyelonephritis. J Am Soc Nephrol. 2011;22(8):1435–1441. doi:10.1681/ASN.2010101072 PMC3148698 21757770

[48] Tamoutounour S Guilliams M Montanana Sanchis F, . Origins and functional specialization of macrophages and of conventional and monocyte-derived dendritic cells in mouse skin. Immunity. 2013;39(5):925–938. doi:10.1016/j.immuni.2013.10.004 24184057

[49] Reed BY Masoumi A Elhassan E, . Angiogenic growth factors correlate with disease severity in young patients with autosomal dominant polycystic kidney disease. Kidney Int. 2011;79(1):128–134. doi:10.1038/ki.2010.355 PMC3815472 20881939

[50] Zimmerman KA Huang J He L, . Interferon regulatory factor-5 in resident macrophage promotes polycystic kidney disease. Kidney360. 2020;1(3):179–190. doi:10.34067/KID.0001052019 PMC7822573 33490963

[51] Han X Li Q Lan X El-Mufti L Ren H Wang J. Microglial depletion with clodronate liposomes increases proinflammatory cytokine levels, induces astrocyte activation, and damages blood vessel integrity. Mol Neurobiol. 2019;56(9):6184–6196. doi:10.1007/s12035-019-1502-9 PMC6684378 30734229

[52] Summan M Warren GL Mercer RR, . Macrophages and skeletal muscle regeneration: a clodronate-containing liposome depletion study. Am J Physiol Regul Integr Comp Physiol. 2006;290(6):R1488–R1495. doi:10.1152/ajpregu.00465.2005 16424086

[53] Ju W Lu W Bao Y, . Clodronate-liposomes aggravate irradiation-induced myelosuppression by promoting myeloid differentiation. Int J Radiat Biol. 2021;97(2):240–248. doi:10.1080/09553002.2021.1857452 33253621

[54] Peng Y Li L Shang J, . Macrophage promotes fibroblast activation and kidney fibrosis by assembling a vitronectin-enriched microenvironment. Theranostics. 2023;13(11):3897–3913. doi:10.7150/thno.85250 PMC10334827 37441594

[55] Dwivedi N Tao S Jamadar A, . Epithelial vasopressin type-2 receptors regulate myofibroblasts by a yap-ccn2-dependent mechanism in polycystic kidney disease. J Am Soc Nephrol. 2020;31(8):1697–1710. doi:10.1681/ASN.2020020190 PMC7460894 32554753

[56] Dwivedi N Jamadar A Mathew S Fields TA Rao R. Myofibroblast depletion reduces kidney cyst growth and fibrosis in autosomal dominant polycystic kidney disease. Kidney Int. 2023;103(1):144–155. doi:10.1016/j.kint.2022.08.036 PMC9822873 36273656

[57] Kleczko EK Marsh KH Tyler LC, . Cd8(+) t cells modulate autosomal dominant polycystic kidney disease progression. Kidney Int. 2018;94(6):1127–1140. doi:10.1016/j.kint.2018.06.025 PMC6319903 30249452

[58] Smith ME Hopp K Zimmerman K. Interferon gamma drives rapidly progressing cystic kidney diseases. J Am Soc Nephrol. 2025;36(10S). doi:10.1681/ASN.2025366xmkpq

