# Supplementary material for: The Kinetics and Mechanism of Niche Filling in Healthy and Cystic Mice
Source: Kidney360. 2026 May 7;7(9):1956–67. doi: 10.34067/KID.0000001217 (PMC13641440; doi:10.34067/KID.0000001217)
Supplement: Supplementary file 2 [file kidney360-7-1956-s002.pdf]

Table of Contents

Supplemental Table 1. Three-way ANOVA for Figure 1B.

Supplemental Table 2. Three-way ANOVA post-hoc multiple comparisons test for Figure 1B.

Supplemental Table 3. One-way ANOVA of healthy male mice for Supplemental Figure 3A.

Supplemental Table 4. One-way ANOVA of healthy female mice for Supplemental Figure 3A.

Supplemental Table 5. One-way ANOVA of cystic male mice for Supplemental Figure 3A.

Supplemental Table 6. One-way ANOVA of cystic female mice for Supplemental Figure 3A.

Supplemental Table 7. Three-way ANOVA for Supplemental Figure 3B.

Supplemental Table 8. Three-way ANOVA post-hoc multiple comparisons test for Supplemental Figure 3B.

Supplemental Table 9. Three-way ANOVA for Figure 1C.

Supplemental Table 10. Three-way ANOVA post-hoc multiple comparisons test for Figure 1C.

Supplemental Table 11. Three-way ANOVA for Figure 1D.

Supplemental Table 12. Three-way ANOVA post-hoc multiple comparisons test for Figure 1D.

Supplemental Table 13. Three-way ANOVA for Figure 1E.

Supplemental Table 14. Three-way ANOVA post-hoc multiple comparisons test for Figure 1E.

Supplemental Table 15. Two-way ANOVA for Figure 2B.

Supplemental Table 16. Two-way ANOVA post-hoc multiple comparisons test for Figure 2B.

Supplemental Table 17. Two-way ANOVA for Figure 2C.

Supplemental Table 18. Two-way ANOVA post-hoc multiple comparisons test for Figure 2C.

Supplemental Table 19. One-way ANOVA and post-hoc multiple comparisons test for Figure 2E.

Supplemental Table 20. One-way ANOVA and post-hoc multiple comparisons test for Figure 2F.

Supplemental Table 21. Welch’s t-tests for Figure 3A-C.

Supplemental Table 22. Welch’s t-tests for Figure 3D-F.

Supplemental Table 23. Primary antibodies used for flow cytometry.

Supplemental Table 24. List of flow cytometry, staining, and immunofluorescence reagents.

Supplemental Table 25. qRT-PCR primer sequences.

Supplemental Figure 1. Flow cytometry gating strategy

(A) Gating strategy used to identify blood Ly6c^{hi} monocytes and confirm efficient TdT labeling. (B) Gating strategy used to identify liver tissue-resident macrophage (Kupffer cells; KCs), liver Ly6c^{hi} infiltrating monocytes (Ly6c^{hi} IMs), and TdT+ KCs. (C) Gating strategy used identify kidney resident macrophages (KRM), kidney Ly6c^{hi} IMs, and TdT+ KRM, and CD11c+ dendritic cells (DCs).

Supplemental Figure 2. Validation of the fate-mapping mouse model; blood and liver data

(A) Quantification of TdT+ labeling in blood Ly6c^{hi} monocytes in 3-month-old non-depleted (control) healthy *Ms4a3*^{Cre} *Rosa*^{TdT} male and female mice by flow cytometry. Expressed as a percentage of TdT+ Ly6c^{hi} monocytes over all Ly6c^{hi} monocytes in blood. (B) Kupffer cell (KC) abundance in control and 3 days post-depletion (D3) healthy *Ms4a3*^{Cre} *Rosa*^{TdT} male and female mice. Expressed as a percentage of live CD45+ cells. (C) Liver Ly6c^{hi} infiltrating monocyte (Ly6c^{hi} IM) abundance in control and D3 healthy *Ms4a3*^{Cre} *Rosa*^{TdT} male and female. Expressed as a percentage of live CD45+ cells. (D) TdT+ labeling in KCs in control and D3 healthy *Ms4a3*^{Cre} *Rosa*^{TdT} male and female mice. Expressed as a percentage of TdT+ KCs over all KCs in the liver. Statistics: Panel A, Welch's t-test. Panels B-E, 2-way ANOVA with Tukey's multiple comparisons test.

Supplemental Figure 3. Validation of KRM niche filling and dendritic cell abundance

(A) Quantification of kidney resident macrophage (KRM) abundance by flow cytometry as a percentage of live CD45+ cells. 3-month-old non-depleted, D90 liposomal vehicle (LV), and D90 liposomal clodronate (LC) groups are compared in each of the four groups of mice. (B) Quantification of CD11c+ DCs by flow cytometry as a percentage of live CD45+ cells.

Statistics: Panel A, 1-way ANOVA with Tukey's multiple comparisons test. Panel B, 3-way ANOVA with Benjamini-Krieger-Yekutieli post-hoc multiple comparisons test.

Supplemental Figure 4. Chemokine comparison between cystic male and female mice

(A) Analysis of *Ccl2*, *Cx3cl1*, and *Csf1* transcripts on D22 in cystic male and female mice. (B) Analysis of transcript sex-dimorphic effects on D22. Statistics: Panel A, Welch's t-test. Panel B, 1-way ANOVA with Tukey's multiple comparisons test.

Supplemental Table 1. Three-way ANOVA for Figure 1B.

Table Analyzed	KRM % live CD45+				
Three-way ANOVA	Ordinary				
Alpha	0.05				
Source of Variation	% of total variation	P value	P value summary	Significant?	
Time	67.24	<0.0001	****	Yes	
Genotype	0.5174	0.0780	ns	No	
Sex	0.2115	0.2582	ns	No	
Time x Genotype	3.135	0.0029	**	Yes	
Time x Sex	1.419	0.1321	ns	No	
Genotype x Sex	0.05147	0.5763	ns	No	
Time x Genotype x Sex	0.9929	0.3077	ns	No	
ANOVA table	SS (Type III)	DF	MS	F (DFn, DFd)	P value
Time	8124	5	1625	F (5, 131) = 81.99	P<0.0001
Genotype	62.51	1	62.51	F (1, 131) = 3.154	P=0.0780
Sex	25.55	1	25.55	F (1, 131) = 1.289	P=0.2582
Time x Genotype	378.7	5	75.75	F (5, 131) = 3.822	P=0.0029
Time x Sex	171.4	5	34.28	F (5, 131) = 1.730	P=0.1321
Genotype x Sex	6.219	1	6.219	F (1, 131) = 0.3138	P=0.5763
Time x Genotype x Sex	120.0	5	23.99	F (5, 131) = 1.211	P=0.3077
Residual	2596	131	19.82		

Supplemental Table 2. Three-way ANOVA post-hoc multiple comparisons test for Figure 1B.

Compare each cell mean with every other cell mean				
Number of families	1			
Number of comparisons per family	276			
Q	0.05			
Two-stage linear step-up procedure of Benjamini, Krieger and Yekutieli	Mean diff.	Discovery?	q value	Individual P Value
3mo. old:healthy male vs. 3mo. old:healthy female	-3.856	No	0.0523	0.1027
3mo. old:healthy male vs. 3mo. old:cystic male	-2.187	No	0.1777	0.4187
3mo. old:healthy male vs. 3mo. old:cystic female	-4.567	Yes	0.0315	0.0597
3mo. old:healthy male vs. D3:healthy male	17.13	Yes	<0.0001	<0.0001
3mo. old:healthy male vs. D3:healthy female	16.84	Yes	<0.0001	<0.0001
3mo. old:healthy male vs. D3:cystic male	15.70	Yes	<0.0001	<0.0001
3mo. old:healthy male vs. D3:cystic female	15.43	Yes	<0.0001	<0.0001
3mo. old:healthy male vs. D10:healthy male	19.60	Yes	<0.0001	<0.0001
3mo. old:healthy male vs. D10:healthy female	18.43	Yes	<0.0001	<0.0001
3mo. old:healthy male vs. D10:cystic male	16.83	Yes	<0.0001	<0.0001
3mo. old:healthy male vs. D10:cystic female	11.69	Yes	<0.0001	<0.0001
3mo. old:healthy male vs. D22:healthy male	7.393	Yes	0.0044	0.0069
3mo. old:healthy male vs. D22:healthy female	14.36	Yes	<0.0001	<0.0001
3mo. old:healthy male vs. D22:cystic male	9.343	Yes	0.0002	0.0002
3mo. old:healthy male vs. D22:cystic female	9.073	Yes	0.0007	0.0010
3mo. old:healthy male vs. D42:healthy male	11.58	Yes	<0.0001	<0.0001
3mo. old:healthy male vs. D42:healthy female	9.283	Yes	0.0004	0.0004
3mo. old:healthy male vs. D42:cystic male	7.367	Yes	0.0031	0.0048
3mo. old:healthy male vs. D42:cystic female	5.547	Yes	0.0227	0.0416
3mo. old:healthy male vs. D90:healthy male	-1.381	No	0.2261	0.5781
3mo. old:healthy male vs. D90:healthy female	-3.317	No	0.1146	0.2505
3mo. old:healthy male vs. D90:cystic male	1.800	No	0.1855	0.4443
3mo. old:healthy male vs. D90:cystic female	4.023	Yes	0.0422	0.0824
3mo. old:healthy female vs. 3mo. old:cystic male	1.669	No	0.2030	0.5027
3mo. old:healthy female vs. 3mo. old:cystic female	-0.7111	No	0.2796	0.7429
3mo. old:healthy female vs. D3:healthy male	20.99	Yes	<0.0001	<0.0001
3mo. old:healthy female vs. D3:healthy female	20.69	Yes	<0.0001	<0.0001
3mo. old:healthy female vs. D3:cystic male	19.55	Yes	<0.0001	<0.0001
3mo. old:healthy female vs. D3:cystic female	19.29	Yes	<0.0001	<0.0001
3mo. old:healthy female vs. D10:healthy male	23.45	Yes	<0.0001	<0.0001
3mo. old:healthy female vs. D10:healthy female	22.28	Yes	<0.0001	<0.0001
3mo. old:healthy female vs. D10:cystic male	20.68	Yes	<0.0001	<0.0001
3mo. old:healthy female vs. D10:cystic female	15.55	Yes	<0.0001	<0.0001
3mo. old:healthy female vs. D22:healthy male	11.25	Yes	<0.0001	<0.0001
3mo. old:healthy female vs. D22:healthy female	18.22	Yes	<0.0001	<0.0001
3mo. old:healthy female vs. D22:cystic male	13.20	Yes	<0.0001	<0.0001
3mo. old:healthy female vs. D22:cystic female	12.93	Yes	<0.0001	<0.0001
3mo. old:healthy female vs. D42:healthy male	15.44	Yes	<0.0001	<0.0001
3mo. old:healthy female vs. D42:healthy female	13.14	Yes	<0.0001	<0.0001
3mo. old:healthy female vs. D42:cystic male	11.22	Yes	<0.0001	<0.0001
3mo. old:healthy female vs. D42:cystic female	9.403	Yes	0.0002	0.0002
3mo. old:healthy female vs. D90:healthy male	2.475	No	0.1239	0.2720

3mo. old:healthy female vs. D90:healthy female	0.5389	No	0.3141	0.8407
3mo. old:healthy female vs. D90:cystic male	5.656	Yes	0.0050	0.0080
3mo. old:healthy female vs. D90:cystic female	7.879	Yes	0.0002	0.0002
3mo. old:cystic male vs. 3mo. old:cystic female	-2.380	No	0.1532	0.3501
3mo. old:cystic male vs. D3:healthy male	19.32	Yes	<0.0001	<0.0001
3mo. old:cystic male vs. D3:healthy female	19.02	Yes	<0.0001	<0.0001
3mo. old:cystic male vs. D3:cystic male	17.88	Yes	<0.0001	<0.0001
3mo. old:cystic male vs. D3:cystic female	17.62	Yes	<0.0001	<0.0001
3mo. old:cystic male vs. D10:healthy male	21.79	Yes	<0.0001	<0.0001
3mo. old:cystic male vs. D10:healthy female	20.61	Yes	<0.0001	<0.0001
3mo. old:cystic male vs. D10:cystic male	19.01	Yes	<0.0001	<0.0001
3mo. old:cystic male vs. D10:cystic female	13.88	Yes	<0.0001	<0.0001
3mo. old:cystic male vs. D22:healthy male	9.580	Yes	0.0007	0.0009
3mo. old:cystic male vs. D22:healthy female	16.55	Yes	<0.0001	<0.0001
3mo. old:cystic male vs. D22:cystic male	11.53	Yes	<0.0001	<0.0001
3mo. old:cystic male vs. D22:cystic female	11.26	Yes	<0.0001	0.0001
3mo. old:cystic male vs. D42:healthy male	13.77	Yes	<0.0001	<0.0001
3mo. old:cystic male vs. D42:healthy female	11.47	Yes	<0.0001	<0.0001
3mo. old:cystic male vs. D42:cystic male	9.553	Yes	0.0004	0.0005
3mo. old:cystic male vs. D42:cystic female	7.734	Yes	0.0044	0.0069
3mo. old:cystic male vs. D90:healthy male	0.8057	No	0.2842	0.7577
3mo. old:cystic male vs. D90:healthy female	-1.130	No	0.2667	0.7057
3mo. old:cystic male vs. D90:cystic male	3.987	No	0.0559	0.1108
3mo. old:cystic male vs. D90:cystic female	6.210	Yes	0.0072	0.0120
3mo. old:cystic female vs. D3:healthy male	21.70	Yes	<0.0001	<0.0001
3mo. old:cystic female vs. D3:healthy female	21.40	Yes	<0.0001	<0.0001
3mo. old:cystic female vs. D3:cystic male	20.26	Yes	<0.0001	<0.0001
3mo. old:cystic female vs. D3:cystic female	20.00	Yes	<0.0001	<0.0001
3mo. old:cystic female vs. D10:healthy male	24.17	Yes	<0.0001	<0.0001
3mo. old:cystic female vs. D10:healthy female	22.99	Yes	<0.0001	<0.0001
3mo. old:cystic female vs. D10:cystic male	21.39	Yes	<0.0001	<0.0001
3mo. old:cystic female vs. D10:cystic female	16.26	Yes	<0.0001	<0.0001
3mo. old:cystic female vs. D22:healthy male	11.96	Yes	<0.0001	<0.0001
3mo. old:cystic female vs. D22:healthy female	18.93	Yes	<0.0001	<0.0001
3mo. old:cystic female vs. D22:cystic male	13.91	Yes	<0.0001	<0.0001
3mo. old:cystic female vs. D22:cystic female	13.64	Yes	<0.0001	<0.0001
3mo. old:cystic female vs. D42:healthy male	16.15	Yes	<0.0001	<0.0001
3mo. old:cystic female vs. D42:healthy female	13.85	Yes	<0.0001	<0.0001
3mo. old:cystic female vs. D42:cystic male	11.93	Yes	<0.0001	<0.0001
3mo. old:cystic female vs. D42:cystic female	10.11	Yes	0.0001	0.0001
3mo. old:cystic female vs. D90:healthy male	3.186	No	0.0793	0.1691
3mo. old:cystic female vs. D90:healthy female	1.250	No	0.2480	0.6473
3mo. old:cystic female vs. D90:cystic male	6.367	Yes	0.0026	0.0038
3mo. old:cystic female vs. D90:cystic female	8.590	Yes	<0.0001	<0.0001
D3:healthy male vs. D3:healthy female	-0.2971	No	0.3288	0.8843
D3:healthy male vs. D3:cystic male	-1.437	No	0.1978	0.4819
D3:healthy male vs. D3:cystic female	-1.700	No	0.1900	0.4609
D3:healthy male vs. D10:healthy male	2.465	No	0.1456	0.3269
D3:healthy male vs. D10:healthy female	1.292	No	0.2258	0.5751
D3:healthy male vs. D10:cystic male	-0.3067	No	0.3288	0.8868

D3:healthy male vs. D10:cystic female	-5.442	Yes	0.0112	0.0194
D3:healthy male vs. D22:healthy male	-9.740	Yes	<0.0001	<0.0001
D3:healthy male vs. D22:healthy female	-2.772	No	0.1058	0.2301
D3:healthy male vs. D22:cystic male	-7.790	Yes	0.0002	0.0002
D3:healthy male vs. D22:cystic female	-8.060	Yes	0.0005	0.0006
D3:healthy male vs. D42:healthy male	-5.550	Yes	0.0158	0.0285
D3:healthy male vs. D42:healthy female	-7.850	Yes	0.0003	0.0004
D3:healthy male vs. D42:cystic male	-9.767	Yes	<0.0001	<0.0001
D3:healthy male vs. D42:cystic female	-11.59	Yes	<0.0001	<0.0001
D3:healthy male vs. D90:healthy male	-18.51	Yes	<0.0001	<0.0001
D3:healthy male vs. D90:healthy female	-20.45	Yes	<0.0001	<0.0001
D3:healthy male vs. D90:cystic male	-15.33	Yes	<0.0001	<0.0001
D3:healthy male vs. D90:cystic female	-13.11	Yes	<0.0001	<0.0001
D3:healthy female vs. D3:cystic male	-1.140	No	0.2437	0.6327
D3:healthy female vs. D3:cystic female	-1.403	No	0.2304	0.5914
D3:healthy female vs. D10:healthy male	2.762	No	0.1449	0.3240
D3:healthy female vs. D10:healthy female	1.589	No	0.2159	0.5431
D3:healthy female vs. D10:cystic male	-0.009524	No	0.3603	0.9969
D3:healthy female vs. D10:cystic female	-5.145	Yes	0.0272	0.0505
D3:healthy female vs. D22:healthy male	-9.443	Yes	0.0003	0.0004
D3:healthy female vs. D22:healthy female	-2.475	No	0.1515	0.3441
D3:healthy female vs. D22:cystic male	-7.493	Yes	0.0014	0.0020
D3:healthy female vs. D22:cystic female	-7.763	Yes	0.0023	0.0035
D3:healthy female vs. D42:healthy male	-5.253	Yes	0.0324	0.0620
D3:healthy female vs. D42:healthy female	-7.553	Yes	0.0019	0.0028
D3:healthy female vs. D42:cystic male	-9.470	Yes	0.0002	0.0002
D3:healthy female vs. D42:cystic female	-11.29	Yes	<0.0001	<0.0001
D3:healthy female vs. D90:healthy male	-18.22	Yes	<0.0001	<0.0001
D3:healthy female vs. D90:healthy female	-20.15	Yes	<0.0001	<0.0001
D3:healthy female vs. D90:cystic male	-15.04	Yes	<0.0001	<0.0001
D3:healthy female vs. D90:cystic female	-12.81	Yes	<0.0001	<0.0001
D3:cystic male vs. D3:cystic female	-0.2629	No	0.3386	0.9198
D3:cystic male vs. D10:healthy male	3.902	No	0.0784	0.1643
D3:cystic male vs. D10:healthy female	2.729	No	0.1335	0.2970
D3:cystic male vs. D10:cystic male	1.130	No	0.2480	0.6488
D3:cystic male vs. D10:cystic female	-4.005	No	0.0636	0.1268
D3:cystic male vs. D22:healthy male	-8.303	Yes	0.0013	0.0018
D3:cystic male vs. D22:healthy female	-1.335	No	0.2356	0.6094
D3:cystic male vs. D22:cystic male	-6.353	Yes	0.0053	0.0086
D3:cystic male vs. D22:cystic female	-6.623	Yes	0.0073	0.0122
D3:cystic male vs. D42:healthy male	-4.113	No	0.0706	0.1429
D3:cystic male vs. D42:healthy female	-6.413	Yes	0.0065	0.0107
D3:cystic male vs. D42:cystic male	-8.330	Yes	0.0007	0.0010
D3:cystic male vs. D42:cystic female	-10.15	Yes	0.0001	0.0002
D3:cystic male vs. D90:healthy male	-17.08	Yes	<0.0001	<0.0001
D3:cystic male vs. D90:healthy female	-19.01	Yes	<0.0001	<0.0001
D3:cystic male vs. D90:cystic male	-13.90	Yes	<0.0001	<0.0001
D3:cystic male vs. D90:cystic female	-11.67	Yes	<0.0001	<0.0001
D3:cystic female vs. D10:healthy male	4.165	No	0.0786	0.1655
D3:cystic female vs. D10:healthy female	2.992	No	0.1308	0.2899

D3:cystic female vs. D10:cystic male	1.393	No	0.2352	0.6061
D3:cystic female vs. D10:cystic female	-3.742	No	0.0868	0.1861
D3:cystic female vs. D22:healthy male	-8.040	Yes	0.0032	0.0050
D3:cystic female vs. D22:healthy female	-1.072	No	0.2667	0.7040
D3:cystic female vs. D22:cystic male	-6.090	Yes	0.0120	0.0210
D3:cystic female vs. D22:cystic female	-6.360	Yes	0.0143	0.0255
D3:cystic female vs. D42:healthy male	-3.850	No	0.0926	0.1996
D3:cystic female vs. D42:healthy female	-6.150	Yes	0.0136	0.0241
D3:cystic female vs. D42:cystic male	-8.067	Yes	0.0023	0.0033
D3:cystic female vs. D42:cystic female	-9.886	Yes	0.0005	0.0006
D3:cystic female vs. D90:healthy male	-16.81	Yes	<0.0001	<0.0001
D3:cystic female vs. D90:healthy female	-18.75	Yes	<0.0001	<0.0001
D3:cystic female vs. D90:cystic male	-13.63	Yes	<0.0001	<0.0001
D3:cystic female vs. D90:cystic female	-11.41	Yes	<0.0001	<0.0001
D10:healthy male vs. D10:healthy female	-1.173	No	0.2646	0.6951
D10:healthy male vs. D10:cystic male	-2.772	No	0.1492	0.3365
D10:healthy male vs. D10:cystic female	-7.907	Yes	0.0056	0.0091
D10:healthy male vs. D22:healthy male	-12.21	Yes	<0.0001	<0.0001
D10:healthy male vs. D22:healthy female	-5.237	Yes	0.0421	0.0818
D10:healthy male vs. D22:cystic male	-10.26	Yes	0.0003	0.0003
D10:healthy male vs. D22:cystic female	-10.53	Yes	0.0005	0.0006
D10:healthy male vs. D42:healthy male	-8.015	Yes	0.0072	0.0120
D10:healthy male vs. D42:healthy female	-10.32	Yes	0.0004	0.0005
D10:healthy male vs. D42:cystic male	-12.23	Yes	<0.0001	<0.0001
D10:healthy male vs. D42:cystic female	-14.05	Yes	<0.0001	<0.0001
D10:healthy male vs. D90:healthy male	-20.98	Yes	<0.0001	<0.0001
D10:healthy male vs. D90:healthy female	-22.92	Yes	<0.0001	<0.0001
D10:healthy male vs. D90:cystic male	-17.80	Yes	<0.0001	<0.0001
D10:healthy male vs. D90:cystic female	-15.58	Yes	<0.0001	<0.0001
D10:healthy female vs. D10:cystic male	-1.599	No	0.2185	0.5542
D10:healthy female vs. D10:cystic female	-6.734	Yes	0.0106	0.0182
D10:healthy female vs. D22:healthy male	-11.03	Yes	0.0001	0.0001
D10:healthy female vs. D22:healthy female	-4.064	No	0.0733	0.1513
D10:healthy female vs. D22:cystic male	-9.082	Yes	0.0005	0.0007
D10:healthy female vs. D22:cystic female	-9.352	Yes	0.0008	0.0012
D10:healthy female vs. D42:healthy male	-6.842	Yes	0.0134	0.0236
D10:healthy female vs. D42:healthy female	-9.142	Yes	0.0007	0.0009
D10:healthy female vs. D42:cystic male	-11.06	Yes	<0.0001	<0.0001
D10:healthy female vs. D42:cystic female	-12.88	Yes	<0.0001	<0.0001
D10:healthy female vs. D90:healthy male	-19.81	Yes	<0.0001	<0.0001
D10:healthy female vs. D90:healthy female	-21.74	Yes	<0.0001	<0.0001
D10:healthy female vs. D90:cystic male	-16.63	Yes	<0.0001	<0.0001
D10:healthy female vs. D90:cystic female	-14.40	Yes	<0.0001	<0.0001
D10:cystic male vs. D10:cystic female	-5.135	Yes	0.0313	0.0590
D10:cystic male vs. D22:healthy male	-9.433	Yes	0.0005	0.0006
D10:cystic male vs. D22:healthy female	-2.465	No	0.1564	0.3621
D10:cystic male vs. D22:cystic male	-7.483	Yes	0.0021	0.0030
D10:cystic male vs. D22:cystic female	-7.753	Yes	0.0031	0.0047
D10:cystic male vs. D42:healthy male	-5.243	Yes	0.0363	0.0703
D10:cystic male vs. D42:healthy female	-7.543	Yes	0.0026	0.0039

D10:cystic male vs. D42:cystic male	-9.460	Yes	0.0003	0.0003
D10:cystic male vs. D42:cystic female	-11.28	Yes	<0.0001	<0.0001
D10:cystic male vs. D90:healthy male	-18.21	Yes	<0.0001	<0.0001
D10:cystic male vs. D90:healthy female	-20.14	Yes	<0.0001	<0.0001
D10:cystic male vs. D90:cystic male	-15.03	Yes	<0.0001	<0.0001
D10:cystic male vs. D90:cystic female	-12.80	Yes	<0.0001	<0.0001
D10:cystic female vs. D22:healthy male	-4.298	No	0.0645	0.1293
D10:cystic female vs. D22:healthy female	2.670	No	0.1515	0.3447
D10:cystic female vs. D22:cystic male	-2.348	No	0.1588	0.3694
D10:cystic female vs. D22:cystic female	-2.618	No	0.1536	0.3542
D10:cystic female vs. D42:healthy male	-0.1080	No	0.3549	0.9712
D10:cystic female vs. D42:healthy female	-2.408	No	0.1598	0.3733
D10:cystic female vs. D42:cystic male	-4.325	No	0.0559	0.1110
D10:cystic female vs. D42:cystic female	-6.144	Yes	0.0170	0.0309
D10:cystic female vs. D90:healthy male	-13.07	Yes	<0.0001	<0.0001
D10:cystic female vs. D90:healthy female	-15.01	Yes	<0.0001	<0.0001
D10:cystic female vs. D90:cystic male	-9.891	Yes	0.0001	0.0001
D10:cystic female vs. D90:cystic female	-7.668	Yes	0.0014	0.0021
D22:healthy male vs. D22:healthy female	6.968	Yes	0.0087	0.0146
D22:healthy male vs. D22:cystic male	1.950	No	0.1892	0.4557
D22:healthy male vs. D22:cystic female	1.680	No	0.2184	0.5517
D22:healthy male vs. D42:healthy male	4.190	No	0.0781	0.1630
D22:healthy male vs. D42:healthy female	1.890	No	0.1981	0.4845
D22:healthy male vs. D42:cystic male	-0.02667	No	0.3599	0.9921
D22:healthy male vs. D42:cystic female	-1.846	No	0.2064	0.5132
D22:healthy male vs. D90:healthy male	-8.774	Yes	0.0007	0.0010
D22:healthy male vs. D90:healthy female	-10.71	Yes	0.0004	0.0005
D22:healthy male vs. D90:cystic male	-5.593	Yes	0.0145	0.0259
D22:healthy male vs. D90:cystic female	-3.370	No	0.0793	0.1693
D22:healthy female vs. D22:cystic male	-5.018	Yes	0.0302	0.0564
D22:healthy female vs. D22:cystic female	-5.288	Yes	0.0325	0.0626
D22:healthy female vs. D42:healthy male	-2.778	No	0.1536	0.3539
D22:healthy female vs. D42:healthy female	-5.078	Yes	0.0324	0.0618
D22:healthy female vs. D42:cystic male	-6.995	Yes	0.0065	0.0105
D22:healthy female vs. D42:cystic female	-8.814	Yes	0.0015	0.0022
D22:healthy female vs. D90:healthy male	-15.74	Yes	<0.0001	<0.0001
D22:healthy female vs. D90:healthy female	-17.68	Yes	<0.0001	<0.0001
D22:healthy female vs. D90:cystic male	-12.56	Yes	<0.0001	<0.0001
D22:healthy female vs. D90:cystic female	-10.34	Yes	<0.0001	<0.0001
D22:cystic male vs. D22:cystic female	-0.2700	No	0.3386	0.9177
D22:cystic male vs. D42:healthy male	2.240	No	0.1786	0.4235
D22:cystic male vs. D42:healthy female	-0.06000	No	0.3570	0.9807
D22:cystic male vs. D42:cystic male	-1.977	No	0.1786	0.4262
D22:cystic male vs. D42:cystic female	-3.796	No	0.0721	0.1477
D22:cystic male vs. D90:healthy male	-10.72	Yes	<0.0001	<0.0001
D22:cystic male vs. D90:healthy female	-12.66	Yes	<0.0001	<0.0001
D22:cystic male vs. D90:cystic male	-7.543	Yes	0.0007	0.0010
D22:cystic male vs. D90:cystic female	-5.320	Yes	0.0098	0.0167
D22:cystic female vs. D42:healthy male	2.510	No	0.1714	0.4022
D22:cystic female vs. D42:healthy female	0.2100	No	0.3440	0.9380

D22:cystic female vs. D42:cystic male	-1.707	No	0.2114	0.5278
D22:cystic female vs. D42:cystic female	-3.526	No	0.0982	0.2127
D22:cystic female vs. D90:healthy male	-10.45	Yes	<0.0001	0.0001
D22:cystic female vs. D90:healthy female	-12.39	Yes	<0.0001	<0.0001
D22:cystic female vs. D90:cystic male	-7.273	Yes	0.0026	0.0040
D22:cystic female vs. D90:cystic female	-5.050	Yes	0.0221	0.0403
D42:healthy male vs. D42:healthy female	-2.300	No	0.1786	0.4249
D42:healthy male vs. D42:cystic male	-4.217	No	0.0711	0.1447
D42:healthy male vs. D42:cystic female	-6.036	Yes	0.0246	0.0453
D42:healthy male vs. D90:healthy male	-12.96	Yes	<0.0001	<0.0001
D42:healthy male vs. D90:healthy female	-14.90	Yes	<0.0001	<0.0001
D42:healthy male vs. D90:cystic male	-9.783	Yes	0.0003	0.0004
D42:healthy male vs. D90:cystic female	-7.560	Yes	0.0031	0.0048
D42:healthy female vs. D42:cystic male	-1.917	No	0.1892	0.4572
D42:healthy female vs. D42:cystic female	-3.736	No	0.0793	0.1681
D42:healthy female vs. D90:healthy male	-10.66	Yes	<0.0001	<0.0001
D42:healthy female vs. D90:healthy female	-12.60	Yes	<0.0001	<0.0001
D42:healthy female vs. D90:cystic male	-7.483	Yes	0.0013	0.0018
D42:healthy female vs. D90:cystic female	-5.260	Yes	0.0135	0.0237
D42:cystic male vs. D42:cystic female	-1.819	No	0.2030	0.5009
D42:cystic male vs. D90:healthy male	-8.748	Yes	0.0005	0.0006
D42:cystic male vs. D90:healthy female	-10.68	Yes	0.0003	0.0003
D42:cystic male vs. D90:cystic male	-5.567	Yes	0.0111	0.0191
D42:cystic male vs. D90:cystic female	-3.343	No	0.0721	0.1482
D42:cystic female vs. D90:healthy male	-6.928	Yes	0.0055	0.0088
D42:cystic female vs. D90:healthy female	-8.864	Yes	0.0024	0.0036
D42:cystic female vs. D90:cystic male	-3.747	No	0.0663	0.1337
D42:cystic female vs. D90:cystic female	-1.524	No	0.2127	0.5330
D90:healthy male vs. D90:healthy female	-1.936	No	0.1991	0.4891
D90:healthy male vs. D90:cystic male	3.181	No	0.0764	0.1586
D90:healthy male vs. D90:cystic female	5.404	Yes	0.0089	0.0151
D90:healthy female vs. D90:cystic male	5.117	Yes	0.0309	0.0580
D90:healthy female vs. D90:cystic female	7.340	Yes	0.0039	0.0061
D90:cystic male vs. D90:cystic female	2.223	No	0.1265	0.2790

Supplemental Table 3. One-way ANOVA of healthy male mice for Supplemental Figure 3A.

Table Analyzed	Fig S3A - healthy male 3 mo. old, D90 LV, D90 LC KRM % live CD45+				
Data sets analyzed	A-C				
Distribution assumption	Normal (Gaussian)				
ANOVA summary					
F	2.011				
P value	0.1707				
P value summary	ns				
Significant diff. among means (P < 0.05)?	No				
R squared	0.2232				
Brown-Forsythe test					
F (DFn, DFd)	0.4994 (2, 14)				
P value	0.6173				
P value summary	ns				
Are SDs significantly different (P < 0.05)?	No				
Bartlett's test					
Bartlett's statistic (corrected)	1.205				
P value	0.5475				
P value summary	ns				
Are SDs significantly different (P < 0.05)?	No				
ANOVA table	SS	DF	MS	F (DFn, DFd)	P value
Treatment (between columns)	102.0	2	51.01	F (2, 14) = 2.011	P=0.1707
Residual (within columns)	355.1	14	25.36		
Total	457.1	16			
Data summary					
Number of treatments (columns)	3				
Number of values (total)	17				

Number of families	1							
Number of comparisons per family	3							
Alpha	0.05							
Tukey's multiple comparisons test	Mean diff.	95.00% CI of diff.	Below threshold?	Summary	Adjusted P Value			
3mo. old vs. D90 LV	-6.342	-14.85 to 2.167	No	ns	0.1614	A-B		
3mo. old vs. D90 LC	-1.381	-8.715 to 5.953	No	ns	0.8758	A-C		
D90 LV vs. D90 LC	4.961	-3.301 to 13.22	No	ns	0.2898	B-C		
Test details	Mean 1	Mean 2	Mean diff.	SE of diff.	n1	n2	q	DF
3mo. old vs. D90 LV	25.13	31.48	-6.342	3.251	6	4	2.759	14
3mo. old vs. D90 LC	25.13	26.51	-1.381	2.802	6	7	0.6970	14
D90 LV vs. D90 LC	31.48	26.51	4.961	3.157	4	7	2.222	14
Compact letter display								
D90 LV	A							
D90 LC	A							

3mo. old	A							

Supplemental Table 4. One-way ANOVA of healthy female mice for Supplemental Figure 3A.

Table Analyzed	Fig S3B - healthy female 3mo. old, D90 LV, D90 LC KRM % live CD45+				
Data sets analyzed	A-C				
Distribution assumption	Normal (Gaussian)				
ANOVA summary					
F	1.477				
P value	0.2642				
P value summary	ns				
Significant diff. among means (P < 0.05)?	No				
R squared	0.1852				
Brown-Forsythe test					
F (DFn, DFd)	0.1587 (2, 13)				
P value	0.8549				
P value summary	ns				
Are SDs significantly different (P < 0.05)?	No				
Bartlett's test					
Bartlett's statistic (corrected)					
P value					
P value summary					
Are SDs significantly different (P < 0.05)?					
ANOVA table	SS	DF	MS	F (DFn, DFd)	P value
Treatment (between columns)	45.39	2	22.70	F (2, 13) = 1.477	P=0.2642
Residual (within columns)	199.7	13	15.36		
Total	245.1	15			
Data summary					
Number of treatments (columns)	3				
Number of values (total)	16				

Number of families	1							
Number of comparisons per family	3							
Alpha	0.05							
Tukey's multiple comparisons test	Mean diff.	95.00% CI of diff.	Below threshold?	Summary	Adjusted P Value			
3mo. old vs. D90 LV	-4.111	-11.01 to 2.789	No	ns	0.2914	A-B		
3mo. old vs. D90 LC	0.5389	-5.681 to 6.758	No	ns	0.9716	A-C		
D90 LV vs. D90 LC	4.650	-3.255 to 12.55	No	ns	0.2997	B-C		
Test details	Mean 1	Mean 2	Mean diff.	SE of diff.	n1	n2	q	DF
3mo. old vs. D90 LV	28.99	33.10	-4.111	2.613	9	3	2.225	13
3mo. old vs. D90 LC	28.99	28.45	0.5389	2.355	9	4	0.3235	13
D90 LV vs. D90 LC	33.10	28.45	4.650	2.994	3	4	2.197	13
Compact letter display								
D90 LV	A							
3mo. old	A							
D90 LC	A							

Supplemental Table 5. One-way ANOVA of cystic male mice for Supplemental Figure 3A.

Table Analyzed	Fig S3C - cystic male 3mo. old, D90 LV, D90 LC KRM % live CD45+				
Data sets analyzed	A-C				
Distribution assumption	Normal (Gaussian)				
ANOVA summary					
F	0.7424				
P value	0.4875				
P value summary	ns				
Significant diff. among means (P < 0.05)?	No				
R squared	0.06322				
Brown-Forsythe test					
F (DFn, DFd)	1.929 (2, 22)				
P value	0.1691				
P value summary	ns				
Are SDs significantly different (P < 0.05)?	No				
Bartlett's test					
Bartlett's statistic (corrected)	2.983				
P value	0.2250				
P value summary	ns				
Are SDs significantly different (P < 0.05)?	No				
ANOVA table	SS	DF	MS	F (DFn, DFd)	P value
Treatment (between columns)	64.99	2	32.50	F (2, 22) = 0.7424	P=0.4875
Residual (within columns)	963.0	22	43.77		
Total	1028	24			
Data summary					
Number of treatments (columns)	3				
Number of values (total)	25				

Number of families	1							
Number of comparisons per family	3							
Alpha	0.05							
Tukey's multiple comparisons test	Mean diff.	95.00% CI of diff.	Below threshold?	Summary	Adjusted P Value			
3mo. old vs. D90 LV	4.065	-4.899 to 13.03	No	ns	0.5009	A-B		
3mo. old vs. D90 LC	3.987	-5.284 to 13.26	No	ns	0.5359	A-C		
D90 LV vs. D90 LC	-0.07879	-7.549 to 7.391	No	ns	0.9996	B-C		
Test details	Mean 1	Mean 2	Mean diff.	SE of diff.	n1	n2	q	DF
3mo. old vs. D90 LV	27.32	23.25	4.065	3.568	5	11	1.611	22
3mo. old vs. D90 LC	27.32	23.33	3.987	3.690	5	9	1.528	22
D90 LV vs. D90 LC	23.25	23.33	-0.07879	2.974	11	9	0.03747	22
Compact letter display								
3mo. old	A							
D90 LC	A							
D90 LV	A							

Supplemental Table 6. One-way ANOVA of cystic female mice for Supplemental Figure 3A.

Table Analyzed	Fig S3D - cystic female 3mo. old, D90 LV, D90 LC KRM % live CD45+				
Data sets analyzed	A-C				
Distribution assumption	Normal (Gaussian)				
ANOVA summary					
F	4.745				
P value	0.0183				
P value summary	*				
Significant diff. among means (P < 0.05)?	Yes				
R squared	0.2834				
Brown-Forsythe test					
F (DFn, DFd)	3.636 (2, 24)				
P value	0.0418				
P value summary	*				
Are SDs significantly different (P < 0.05)?	Yes				
Bartlett's test					
Bartlett's statistic (corrected)	8.204				
P value	0.0165				
P value summary	*				
Are SDs significantly different (P < 0.05)?	Yes				
ANOVA table	SS	DF	MS	F (DFn, DFd)	P value
Treatment (between columns)	453.9	2	226.9	F (2, 24) = 4.745	P=0.0183
Residual (within columns)	1148	24	47.82		
Total	1602	26			
Data summary					
Number of treatments (columns)	3				
Number of values (total)	27				

Number of families	1							
Number of comparisons per family	3							
Alpha	0.05							
Tukey's multiple comparisons test	Mean diff.	95.00% CI of diff.	Below threshold?	Summary	Adjusted P Value			
3mo. old vs. D90 LV	0.1333	-8.258 to 8.525	No	ns	0.9991	A-B		
3mo. old vs. D90 LC	8.560	0.3682 to 16.75	Yes	*	0.0394	A-C		
D90 LV vs. D90 LC	8.427	0.4917 to 16.36	Yes	*	0.0359	B-C		
Test details	Mean 1	Mean 2	Mean diff.	SE of diff.	n1	n2	q	DF
3mo. old vs. D90 LV	29.70	29.57	0.1333	3.360	8	9	0.05611	24
3mo. old vs. D90 LC	29.70	21.14	8.560	3.280	8	10	3.690	24
D90 LV vs. D90 LC	29.57	21.14	8.427	3.177	9	10	3.751	24
Compact letter display								
3mo. old	A							
D90 LV	A							

D90 LC	B							

Supplemental Table 7. Three-way ANOVA for Supplemental Figure 3B.

Table Analyzed	CD11c+ DCs % live CD45+				
Three-way ANOVA	Ordinary				
Alpha	0.05				
Source of Variation	% of total variation	P value	P value summary	Significant?	
Time	16.66	<0.0001	****	Yes	
Genotype	14.71	<0.0001	****	Yes	
Sex	0.5727	0.1453	ns	No	
Time x Genotype	17.79	<0.0001	****	Yes	
Time x Sex	3.018	0.0518	ns	No	
Genotype x Sex	0.02581	0.7563	ns	No	
Time x Genotype x Sex	3.862	0.0163	*	Yes	
ANOVA table	SS (Type III)	DF	MS	F (DFn, DFd)	P value
Time	305.6	5	61.13	F (5, 134) = 12.49	P<0.0001
Genotype	269.8	1	269.8	F (1, 134) = 55.12	P<0.0001
Sex	10.51	1	10.51	F (1, 134) = 2.146	P=0.1453
Time x Genotype	326.4	5	65.27	F (5, 134) = 13.34	P<0.0001
Time x Sex	55.36	5	11.07	F (5, 134) = 2.262	P=0.0518
Genotype x Sex	0.4735	1	0.4735	F (1, 134) = 0.09674	P=0.7563
Time x Genotype x Sex	70.85	5	14.17	F (5, 134) = 2.895	P=0.0163
Residual	655.9	134	4.895		

Supplemental Table 8. Three-way ANOVA post-hoc multiple comparisons test for Supplemental Figure 3B.

Compare each cell mean with every other cell mean				
Number of families	1			
Number of comparisons per family	276			
Q	0.05			
Two-stage linear step-up procedure of Benjamini, Krieger and Yekutieli	Mean diff.	Discovery?	q value	Individual P Value
3mo. old:healthy male vs. 3mo. old:healthy female	-0.3289	No	0.4710	0.7783
3mo. old:healthy male vs. 3mo. old:cystic male	-4.735	Yes	0.0013	0.0006
3mo. old:healthy male vs. 3mo. old:cystic female	-7.388	Yes	<0.0001	<0.0001
3mo. old:healthy male vs. D3:healthy male	-2.116	No	0.0533	0.0498
3mo. old:healthy male vs. D3:healthy female	-1.289	No	0.2241	0.2969
3mo. old:healthy male vs. D3:cystic male	-10.07	Yes	<0.0001	<0.0001
3mo. old:healthy male vs. D3:cystic female	-7.793	Yes	<0.0001	<0.0001
3mo. old:healthy male vs. D10:healthy male	-5.943	Yes	0.0002	<0.0001
3mo. old:healthy male vs. D10:healthy female	-5.745	Yes	0.0001	<0.0001
3mo. old:healthy male vs. D10:cystic male	-6.095	Yes	<0.0001	<0.0001
3mo. old:healthy male vs. D10:cystic female	-10.70	Yes	<0.0001	<0.0001
3mo. old:healthy male vs. D22:healthy male	-4.157	Yes	0.0039	0.0023
3mo. old:healthy male vs. D22:healthy female	-7.015	Yes	<0.0001	<0.0001
3mo. old:healthy male vs. D22:cystic male	-6.855	Yes	<0.0001	<0.0001
3mo. old:healthy male vs. D22:cystic female	-5.347	Yes	0.0003	0.0001
3mo. old:healthy male vs. D42:healthy male	-2.268	No	0.1044	0.1146
3mo. old:healthy male vs. D42:healthy female	-2.605	Yes	0.0478	0.0434
3mo. old:healthy male vs. D42:cystic male	-3.402	Yes	0.0098	0.0065
3mo. old:healthy male vs. D42:cystic female	-2.691	No	0.0502	0.0466
3mo. old:healthy male vs. D90:healthy male	-4.715	Yes	0.0005	0.0002
3mo. old:healthy male vs. D90:healthy female	-4.781	Yes	0.0021	0.0011
3mo. old:healthy male vs. D90:cystic male	-3.850	Yes	0.0024	0.0012
3mo. old:healthy male vs. D90:cystic female	-5.032	Yes	<0.0001	<0.0001
3mo. old:healthy female vs. 3mo. old:cystic male	-4.406	Yes	0.0012	0.0005
3mo. old:healthy female vs. 3mo. old:cystic female	-7.059	Yes	<0.0001	<0.0001
3mo. old:healthy female vs. D3:healthy male	-1.787	No	0.0598	0.0575
3mo. old:healthy female vs. D3:healthy female	-0.9602	No	0.2841	0.3907
3mo. old:healthy female vs. D3:cystic male	-9.743	Yes	<0.0001	<0.0001
3mo. old:healthy female vs. D3:cystic female	-7.464	Yes	<0.0001	<0.0001
3mo. old:healthy female vs. D10:healthy male	-5.614	Yes	0.0001	<0.0001
3mo. old:healthy female vs. D10:healthy female	-5.416	Yes	<0.0001	<0.0001
3mo. old:healthy female vs. D10:cystic male	-5.766	Yes	<0.0001	<0.0001
3mo. old:healthy female vs. D10:cystic female	-10.37	Yes	<0.0001	<0.0001
3mo. old:healthy female vs. D22:healthy male	-3.828	Yes	0.0039	0.0023
3mo. old:healthy female vs. D22:healthy female	-6.686	Yes	<0.0001	<0.0001
3mo. old:healthy female vs. D22:cystic male	-6.526	Yes	<0.0001	<0.0001
3mo. old:healthy female vs. D22:cystic female	-5.018	Yes	0.0002	<0.0001
3mo. old:healthy female vs. D42:healthy male	-1.939	No	0.1253	0.1470
3mo. old:healthy female vs. D42:healthy female	-2.276	No	0.0564	0.0530
3mo. old:healthy female vs. D42:cystic male	-3.073	Yes	0.0099	0.0067
3mo. old:healthy female vs. D42:cystic female	-2.362	No	0.0598	0.0577

3mo. old:healthy female vs. D90:healthy male	-4.386	Yes	0.0004	0.0001
3mo. old:healthy female vs. D90:healthy female	-4.452	Yes	0.0021	0.0011
3mo. old:healthy female vs. D90:cystic male	-3.521	Yes	0.0020	0.0010
3mo. old:healthy female vs. D90:cystic female	-4.703	Yes	<0.0001	<0.0001
3mo. old:cystic male vs. 3mo. old:cystic female	-2.653	Yes	0.0428	0.0373
3mo. old:cystic male vs. D3:healthy male	2.619	Yes	0.0292	0.0234
3mo. old:cystic male vs. D3:healthy female	3.446	Yes	0.0127	0.0088
3mo. old:cystic male vs. D3:cystic male	-5.337	Yes	0.0001	<0.0001
3mo. old:cystic male vs. D3:cystic female	-3.058	Yes	0.0365	0.0306
3mo. old:cystic male vs. D10:healthy male	-1.208	No	0.2966	0.4171
3mo. old:cystic male vs. D10:healthy female	-1.010	No	0.3251	0.4717
3mo. old:cystic male vs. D10:cystic male	-1.359	No	0.2241	0.2959
3mo. old:cystic male vs. D10:cystic female	-5.968	Yes	0.0001	<0.0001
3mo. old:cystic male vs. D22:healthy male	0.5780	No	0.4263	0.6802
3mo. old:cystic male vs. D22:healthy female	-2.280	No	0.0985	0.1056
3mo. old:cystic male vs. D22:cystic male	-2.119	No	0.0977	0.1042
3mo. old:cystic male vs. D22:cystic female	-0.6120	No	0.4202	0.6625
3mo. old:cystic male vs. D42:healthy male	2.467	No	0.0938	0.0988
3mo. old:cystic male vs. D42:healthy female	2.130	No	0.1044	0.1141
3mo. old:cystic male vs. D42:cystic male	1.333	No	0.2293	0.3052
3mo. old:cystic male vs. D42:cystic female	2.044	No	0.1253	0.1464
3mo. old:cystic male vs. D90:healthy male	0.02057	No	0.5672	0.9874
3mo. old:cystic male vs. D90:healthy female	-0.04550	No	0.5625	0.9756
3mo. old:cystic male vs. D90:cystic male	0.8853	No	0.3256	0.4743
3mo. old:cystic male vs. D90:cystic female	-0.2970	No	0.4809	0.8068
3mo. old:cystic female vs. D3:healthy male	5.272	Yes	<0.0001	<0.0001
3mo. old:cystic female vs. D3:healthy female	6.099	Yes	<0.0001	<0.0001
3mo. old:cystic female vs. D3:cystic male	-2.684	Yes	0.0223	0.0166
3mo. old:cystic female vs. D3:cystic female	-0.4050	No	0.4618	0.7486
3mo. old:cystic female vs. D10:healthy male	1.445	No	0.2207	0.2881
3mo. old:cystic female vs. D10:healthy female	1.643	No	0.1635	0.1949
3mo. old:cystic female vs. D10:cystic male	1.294	No	0.2045	0.2606
3mo. old:cystic female vs. D10:cystic female	-3.315	Yes	0.0136	0.0096
3mo. old:cystic female vs. D22:healthy male	3.231	Yes	0.0157	0.0115
3mo. old:cystic female vs. D22:healthy female	0.3730	No	0.4710	0.7679
3mo. old:cystic female vs. D22:cystic male	0.5336	No	0.4104	0.6420
3mo. old:cystic female vs. D22:cystic female	2.041	No	0.1001	0.1080
3mo. old:cystic female vs. D42:healthy male	5.120	Yes	0.0006	0.0002
3mo. old:cystic female vs. D42:healthy female	4.783	Yes	0.0003	0.0001
3mo. old:cystic female vs. D42:cystic male	3.986	Yes	0.0015	0.0007
3mo. old:cystic female vs. D42:cystic female	4.697	Yes	0.0007	0.0003
3mo. old:cystic female vs. D90:healthy male	2.674	Yes	0.0267	0.0210
3mo. old:cystic female vs. D90:healthy female	2.608	No	0.0592	0.0564
3mo. old:cystic female vs. D90:cystic male	3.538	Yes	0.0024	0.0013
3mo. old:cystic female vs. D90:cystic female	2.356	Yes	0.0322	0.0264
D3:healthy male vs. D3:healthy female	0.8270	No	0.2966	0.4156
D3:healthy male vs. D3:cystic male	-7.956	Yes	<0.0001	<0.0001
D3:healthy male vs. D3:cystic female	-5.677	Yes	<0.0001	<0.0001
D3:healthy male vs. D10:healthy male	-3.827	Yes	0.0042	0.0026
D3:healthy male vs. D10:healthy female	-3.629	Yes	0.0032	0.0018

D3:healthy male vs. D10:cystic male	-3.979	Yes	0.0004	0.0001
D3:healthy male vs. D10:cystic female	-8.587	Yes	<0.0001	<0.0001
D3:healthy male vs. D22:healthy male	-2.041	No	0.0751	0.0762
D3:healthy male vs. D22:healthy female	-4.899	Yes	0.0001	<0.0001
D3:healthy male vs. D22:cystic male	-4.739	Yes	<0.0001	<0.0001
D3:healthy male vs. D22:cystic female	-3.231	Yes	0.0084	0.0054
D3:healthy male vs. D42:healthy male	-0.1523	No	0.5282	0.9028
D3:healthy male vs. D42:healthy female	-0.4890	No	0.4126	0.6480
D3:healthy male vs. D42:cystic male	-1.286	No	0.1713	0.2064
D3:healthy male vs. D42:cystic female	-0.5753	No	0.3980	0.6154
D3:healthy male vs. D90:healthy male	-2.599	Yes	0.0157	0.0114
D3:healthy male vs. D90:healthy female	-2.665	Yes	0.0401	0.0341
D3:healthy male vs. D90:cystic male	-1.734	No	0.0667	0.0652
D3:healthy male vs. D90:cystic female	-2.916	Yes	0.0029	0.0016
D3:healthy female vs. D3:cystic male	-8.783	Yes	<0.0001	<0.0001
D3:healthy female vs. D3:cystic female	-6.504	Yes	<0.0001	<0.0001
D3:healthy female vs. D10:healthy male	-4.654	Yes	0.0021	0.0010
D3:healthy female vs. D10:healthy female	-4.456	Yes	0.0017	0.0008
D3:healthy female vs. D10:cystic male	-4.806	Yes	0.0002	<0.0001
D3:healthy female vs. D10:cystic female	-9.414	Yes	<0.0001	<0.0001
D3:healthy female vs. D22:healthy male	-2.868	Yes	0.0345	0.0285
D3:healthy female vs. D22:healthy female	-5.726	Yes	<0.0001	<0.0001
D3:healthy female vs. D22:cystic male	-5.566	Yes	<0.0001	<0.0001
D3:healthy female vs. D22:cystic female	-4.058	Yes	0.0037	0.0021
D3:healthy female vs. D42:healthy male	-0.9793	No	0.3275	0.4813
D3:healthy female vs. D42:healthy female	-1.316	No	0.2207	0.2869
D3:healthy female vs. D42:cystic male	-2.113	No	0.0751	0.0763
D3:healthy female vs. D42:cystic female	-1.402	No	0.2184	0.2810
D3:healthy female vs. D90:healthy male	-3.426	Yes	0.0070	0.0044
D3:healthy female vs. D90:healthy female	-3.492	Yes	0.0176	0.0130
D3:healthy female vs. D90:cystic male	-2.561	Yes	0.0292	0.0232
D3:healthy female vs. D90:cystic female	-3.743	Yes	0.0017	0.0008
D3:cystic male vs. D3:cystic female	2.279	No	0.0728	0.0730
D3:cystic male vs. D10:healthy male	4.129	Yes	0.0045	0.0028
D3:cystic male vs. D10:healthy female	4.327	Yes	0.0017	0.0008
D3:cystic male vs. D10:cystic male	3.977	Yes	0.0015	0.0007
D3:cystic male vs. D10:cystic female	-0.6313	No	0.3980	0.6175
D3:cystic male vs. D22:healthy male	5.915	Yes	<0.0001	<0.0001
D3:cystic male vs. D22:healthy female	3.057	Yes	0.0223	0.0167
D3:cystic male vs. D22:cystic male	3.217	Yes	0.0088	0.0057
D3:cystic male vs. D22:cystic female	4.725	Yes	0.0007	0.0003
D3:cystic male vs. D42:healthy male	7.804	Yes	<0.0001	<0.0001
D3:cystic male vs. D42:healthy female	7.467	Yes	<0.0001	<0.0001
D3:cystic male vs. D42:cystic male	6.670	Yes	<0.0001	<0.0001
D3:cystic male vs. D42:cystic female	7.381	Yes	<0.0001	<0.0001
D3:cystic male vs. D90:healthy male	5.357	Yes	<0.0001	<0.0001
D3:cystic male vs. D90:healthy female	5.291	Yes	0.0004	0.0001
D3:cystic male vs. D90:cystic male	6.222	Yes	<0.0001	<0.0001
D3:cystic male vs. D90:cystic female	5.040	Yes	<0.0001	<0.0001
D3:cystic female vs. D10:healthy male	1.850	No	0.1773	0.2147

D3:cystic female vs. D10:healthy female	2.048	No	0.1253	0.1456
D3:cystic female vs. D10:cystic male	1.699	No	0.1620	0.1920
D3:cystic female vs. D10:cystic female	-2.910	Yes	0.0447	0.0395
D3:cystic female vs. D22:healthy male	3.636	Yes	0.0146	0.0104
D3:cystic female vs. D22:healthy female	0.7780	No	0.3779	0.5791
D3:cystic female vs. D22:cystic male	0.9386	No	0.3251	0.4700
D3:cystic female vs. D22:cystic female	2.446	No	0.0800	0.0827
D3:cystic female vs. D42:healthy male	5.525	Yes	0.0007	0.0003
D3:cystic female vs. D42:healthy female	5.188	Yes	0.0005	0.0002
D3:cystic female vs. D42:cystic male	4.391	Yes	0.0019	0.0009
D3:cystic female vs. D42:cystic female	5.102	Yes	0.0009	0.0004
D3:cystic female vs. D90:healthy male	3.079	Yes	0.0250	0.0189
D3:cystic female vs. D90:healthy female	3.013	Yes	0.0485	0.0444
D3:cystic female vs. D90:cystic male	3.943	Yes	0.0031	0.0017
D3:cystic female vs. D90:cystic female	2.761	Yes	0.0299	0.0243
D10:healthy male vs. D10:healthy female	0.1980	No	0.5270	0.8941
D10:healthy male vs. D10:cystic male	-0.1514	No	0.5323	0.9132
D10:healthy male vs. D10:cystic female	-4.760	Yes	0.0030	0.0017
D10:healthy male vs. D22:healthy male	1.786	No	0.1887	0.2309
D10:healthy male vs. D22:healthy female	-1.072	No	0.3251	0.4714
D10:healthy male vs. D22:cystic male	-0.9114	No	0.3441	0.5121
D10:healthy male vs. D22:cystic female	0.5960	No	0.4282	0.6886
D10:healthy male vs. D42:healthy male	3.675	Yes	0.0266	0.0203
D10:healthy male vs. D42:healthy female	3.338	Yes	0.0267	0.0209
D10:healthy male vs. D42:cystic male	2.541	No	0.0702	0.0691
D10:healthy male vs. D42:cystic female	3.252	Yes	0.0362	0.0302
D10:healthy male vs. D90:healthy male	1.229	No	0.2756	0.3772
D10:healthy male vs. D90:healthy female	1.163	No	0.3218	0.4587
D10:healthy male vs. D90:cystic male	2.093	No	0.1067	0.1177
D10:healthy male vs. D90:cystic female	0.9110	No	0.3290	0.4876
D10:healthy female vs. D10:cystic male	-0.3494	No	0.4749	0.7878
D10:healthy female vs. D10:cystic female	-4.958	Yes	0.0013	0.0005
D10:healthy female vs. D22:healthy male	1.588	No	0.2039	0.2584
D10:healthy female vs. D22:healthy female	-1.270	No	0.2684	0.3657
D10:healthy female vs. D22:cystic male	-1.109	No	0.2847	0.3933
D10:healthy female vs. D22:cystic female	0.3980	No	0.4710	0.7765
D10:healthy female vs. D42:healthy male	3.477	Yes	0.0266	0.0206
D10:healthy female vs. D42:healthy female	3.140	Yes	0.0266	0.0205
D10:healthy female vs. D42:cystic male	2.343	No	0.0728	0.0727
D10:healthy female vs. D42:cystic female	3.054	Yes	0.0365	0.0308
D10:healthy female vs. D90:healthy male	1.031	No	0.3027	0.4277
D10:healthy female vs. D90:healthy female	0.9645	No	0.3454	0.5169
D10:healthy female vs. D90:cystic male	1.895	No	0.1137	0.1269
D10:healthy female vs. D90:cystic female	0.7130	No	0.3681	0.5573
D10:cystic male vs. D10:cystic female	-4.609	Yes	0.0012	0.0005
D10:cystic male vs. D22:healthy male	1.937	No	0.1188	0.1371
D10:cystic male vs. D22:healthy female	-0.9206	No	0.3270	0.4786
D10:cystic male vs. D22:cystic male	-0.7600	No	0.3460	0.5215
D10:cystic male vs. D22:cystic female	0.7474	No	0.3701	0.5649
D10:cystic male vs. D42:healthy male	3.826	Yes	0.0099	0.0066

D10:cystic male vs. D42:healthy female	3.490	Yes	0.0083	0.0053
D10:cystic male vs. D42:cystic male	2.693	Yes	0.0299	0.0244
D10:cystic male vs. D42:cystic female	3.403	Yes	0.0136	0.0096
D10:cystic male vs. D90:healthy male	1.380	No	0.1967	0.2453
D10:cystic male vs. D90:healthy female	1.314	No	0.2557	0.3451
D10:cystic male vs. D90:cystic male	2.245	No	0.0500	0.0461
D10:cystic male vs. D90:cystic female	1.062	No	0.2468	0.3316
D10:cystic female vs. D22:healthy male	6.546	Yes	<0.0001	<0.0001
D10:cystic female vs. D22:healthy female	3.688	Yes	0.0135	0.0094
D10:cystic female vs. D22:cystic male	3.849	Yes	0.0057	0.0035
D10:cystic female vs. D22:cystic female	5.356	Yes	0.0005	0.0002
D10:cystic female vs. D42:healthy male	8.435	Yes	<0.0001	<0.0001
D10:cystic female vs. D42:healthy female	8.098	Yes	<0.0001	<0.0001
D10:cystic female vs. D42:cystic male	7.301	Yes	<0.0001	<0.0001
D10:cystic female vs. D42:cystic female	8.012	Yes	<0.0001	<0.0001
D10:cystic female vs. D90:healthy male	5.989	Yes	<0.0001	<0.0001
D10:cystic female vs. D90:healthy female	5.923	Yes	0.0003	0.0001
D10:cystic female vs. D90:cystic male	6.853	Yes	<0.0001	<0.0001
D10:cystic female vs. D90:cystic female	5.671	Yes	<0.0001	<0.0001
D22:healthy male vs. D22:healthy female	-2.858	Yes	0.0477	0.0431
D22:healthy male vs. D22:cystic male	-2.697	Yes	0.0447	0.0392
D22:healthy male vs. D22:cystic female	-1.190	No	0.2858	0.3966
D22:healthy male vs. D42:healthy male	1.889	No	0.1713	0.2053
D22:healthy male vs. D42:healthy female	1.552	No	0.1971	0.2486
D22:healthy male vs. D42:cystic male	0.7554	No	0.3689	0.5608
D22:healthy male vs. D42:cystic female	1.466	No	0.2241	0.2967
D22:healthy male vs. D90:healthy male	-0.5574	No	0.4217	0.6677
D22:healthy male vs. D90:healthy female	-0.6235	No	0.4247	0.6751
D22:healthy male vs. D90:cystic male	0.3073	No	0.4809	0.8037
D22:healthy male vs. D90:cystic female	-0.8750	No	0.3251	0.4715
D22:healthy female vs. D22:cystic male	0.1606	No	0.5282	0.9015
D22:healthy female vs. D22:cystic female	1.668	No	0.1911	0.2353
D22:healthy female vs. D42:healthy male	4.747	Yes	0.0031	0.0017
D22:healthy female vs. D42:healthy female	4.410	Yes	0.0024	0.0013
D22:healthy female vs. D42:cystic male	3.613	Yes	0.0092	0.0061
D22:healthy female vs. D42:cystic female	4.324	Yes	0.0041	0.0024
D22:healthy female vs. D90:healthy male	2.301	No	0.0764	0.0780
D22:healthy female vs. D90:healthy female	2.235	No	0.1188	0.1345
D22:healthy female vs. D90:cystic male	3.165	Yes	0.0157	0.0114
D22:healthy female vs. D90:cystic female	1.983	No	0.0977	0.1041
D22:cystic male vs. D22:cystic female	1.507	No	0.1967	0.2466
D22:cystic male vs. D42:healthy male	4.586	Yes	0.0023	0.0012
D22:cystic male vs. D42:healthy female	4.250	Yes	0.0016	0.0007
D22:cystic male vs. D42:cystic male	3.453	Yes	0.0066	0.0041
D22:cystic male vs. D42:cystic female	4.163	Yes	0.0030	0.0016
D22:cystic male vs. D90:healthy male	2.140	No	0.0728	0.0726
D22:cystic male vs. D90:healthy female	2.074	No	0.1188	0.1371
D22:cystic male vs. D90:cystic male	3.005	Yes	0.0117	0.0079
D22:cystic male vs. D90:cystic female	1.822	No	0.0926	0.0970
D22:cystic female vs. D42:healthy male	3.079	Yes	0.0449	0.0399

D22:cystic female vs. D42:healthy female	2.742	Yes	0.0476	0.0426
D22:cystic female vs. D42:cystic male	1.945	No	0.1188	0.1355
D22:cystic female vs. D42:cystic female	2.656	No	0.0616	0.0598
D22:cystic female vs. D90:healthy male	0.6326	No	0.4019	0.6261
D22:cystic female vs. D90:healthy female	0.5665	No	0.4356	0.7033
D22:cystic female vs. D90:cystic male	1.497	No	0.1866	0.2271
D22:cystic female vs. D90:cystic female	0.3150	No	0.4776	0.7953
D42:healthy male vs. D42:healthy female	-0.3367	No	0.4834	0.8140
D42:healthy male vs. D42:cystic male	-1.134	No	0.2966	0.4151
D42:healthy male vs. D42:cystic female	-0.4230	No	0.4710	0.7761
D42:healthy male vs. D90:healthy male	-2.446	No	0.0778	0.0800
D42:healthy male vs. D90:healthy female	-2.513	No	0.1020	0.1106
D42:healthy male vs. D90:cystic male	-1.582	No	0.1911	0.2363
D42:healthy male vs. D90:cystic female	-2.764	Yes	0.0423	0.0366
D42:healthy female vs. D42:cystic male	-0.7969	No	0.3454	0.5185
D42:healthy female vs. D42:cystic female	-0.08633	No	0.5510	0.9487
D42:healthy female vs. D90:healthy male	-2.110	No	0.0854	0.0888
D42:healthy female vs. D90:healthy female	-2.176	No	0.1158	0.1300
D42:healthy female vs. D90:cystic male	-1.245	No	0.2207	0.2876
D42:healthy female vs. D90:cystic female	-2.427	Yes	0.0413	0.0355
D42:cystic male vs. D42:cystic female	0.7106	No	0.3796	0.5843
D42:cystic male vs. D90:healthy male	-1.313	No	0.2100	0.2689
D42:cystic male vs. D90:healthy female	-1.379	No	0.2407	0.3218
D42:cystic male vs. D90:cystic male	-0.4481	No	0.4282	0.6884
D42:cystic male vs. D90:cystic female	-1.630	No	0.1188	0.1372
D42:cystic female vs. D90:healthy male	-2.023	No	0.1087	0.1207
D42:cystic female vs. D90:healthy female	-2.090	No	0.1369	0.1615
D42:cystic female vs. D90:cystic male	-1.159	No	0.2577	0.3494
D42:cystic female vs. D90:cystic female	-2.341	No	0.0586	0.0555
D90:healthy male vs. D90:healthy female	-0.06607	No	0.5567	0.9621
D90:healthy male vs. D90:cystic male	0.8648	No	0.3096	0.4393
D90:healthy male vs. D90:cystic female	-0.3176	No	0.4710	0.7713
D90:healthy female vs. D90:cystic male	0.9308	No	0.3287	0.4850
D90:healthy female vs. D90:cystic female	-0.2515	No	0.5016	0.8479
D90:cystic male vs. D90:cystic female	-1.182	No	0.1967	0.2468

Supplemental Table 9. Three-way ANOVA for Figure 1C.

Table Analyzed	Kidney Ly6chi mono % live CD45+				
Three-way ANOVA	Ordinary				
Alpha	0.05				
Source of Variation	% of total variation	P value	P value summary	Significant?	
Time	43.24	<0.0001	****	Yes	
Genotype	3.397	0.0005	***	Yes	
Sex	1.230	0.0336	*	Yes	
Time x Genotype	4.641	0.0055	**	Yes	
Time x Sex	0.2188	0.9753	ns	No	
Genotype x Sex	0.1243	0.4960	ns	No	
Time x Genotype x Sex	1.004	0.5853	ns	No	
ANOVA table	SS (Type III)	DF	MS	F (DFn, DFd)	P value
Time	540.1	5	108.0	F (5, 130) = 32.42	P<0.0001
Genotype	42.43	1	42.43	F (1, 130) = 12.74	P=0.0005
Sex	15.37	1	15.37	F (1, 130) = 4.613	P=0.0336
Time x Genotype	57.97	5	11.59	F (5, 130) = 3.480	P=0.0055
Time x Sex	2.734	5	0.5467	F (5, 130) = 0.1641	P=0.9753
Genotype x Sex	1.553	1	1.553	F (1, 130) = 0.4661	P=0.4960
Time x Genotype x Sex	12.54	5	2.509	F (5, 130) = 0.7531	P=0.5853
Residual	433.1	130	3.331		

Supplemental Table 10. Three-way ANOVA post-hoc multiple comparisons test for Figure 1C.

Compare each cell mean with every other cell mean				
Number of families	1			
Number of comparisons per family	276			
Q	0.05			
Two-stage linear step-up procedure of Benjamini, Krieger and Yekutieli	Mean diff.	Discovery?	q value	Individual P Value
3mo. old:healthy male vs. 3mo. old:healthy female	-0.1872	No	0.5278	0.8460
3mo. old:healthy male vs. 3mo. old:cystic male	0.1170	No	0.5583	0.9159
3mo. old:healthy male vs. 3mo. old:cystic female	0.9225	No	0.2760	0.3511
3mo. old:healthy male vs. D3:healthy male	-6.584	Yes	<0.0001	<0.0001
3mo. old:healthy male vs. D3:healthy female	-5.596	Yes	<0.0001	<0.0001
3mo. old:healthy male vs. D3:cystic male	-5.126	Yes	<0.0001	<0.0001
3mo. old:healthy male vs. D3:cystic female	-4.215	Yes	0.0006	0.0002
3mo. old:healthy male vs. D10:healthy male	-4.898	Yes	0.0002	<0.0001
3mo. old:healthy male vs. D10:healthy female	-4.221	Yes	0.0006	0.0002
3mo. old:healthy male vs. D10:cystic male	-2.712	Yes	0.0175	0.0112
3mo. old:healthy male vs. D10:cystic female	-1.525	No	0.1628	0.1700
3mo. old:healthy male vs. D22:healthy male	-1.301	No	0.2089	0.2413
3mo. old:healthy male vs. D22:healthy female	-1.869	No	0.1018	0.0932
3mo. old:healthy male vs. D22:cystic male	-3.299	Yes	0.0031	0.0015
3mo. old:healthy male vs. D22:cystic female	-1.165	No	0.2403	0.2938
3mo. old:healthy male vs. D42:healthy male	-3.388	Yes	0.0083	0.0047
3mo. old:healthy male vs. D42:healthy female	-2.525	Yes	0.0255	0.0180
3mo. old:healthy male vs. D42:cystic male	0.2207	No	0.5266	0.8283
3mo. old:healthy male vs. D42:cystic female	-0.1170	No	0.5583	0.9159
3mo. old:healthy male vs. D90:healthy male	-1.092	No	0.2342	0.2841
3mo. old:healthy male vs. D90:healthy female	-0.1550	No	0.5521	0.8955
3mo. old:healthy male vs. D90:cystic male	-1.127	No	0.2091	0.2434
3mo. old:healthy male vs. D90:cystic female	-0.5950	No	0.3822	0.5472
3mo. old:healthy female vs. 3mo. old:cystic male	0.3042	No	0.4909	0.7655
3mo. old:healthy female vs. 3mo. old:cystic female	1.110	No	0.1927	0.2131
3mo. old:healthy female vs. D3:healthy male	-6.396	Yes	<0.0001	<0.0001
3mo. old:healthy female vs. D3:healthy female	-5.409	Yes	<0.0001	<0.0001
3mo. old:healthy female vs. D3:cystic male	-4.939	Yes	<0.0001	<0.0001
3mo. old:healthy female vs. D3:cystic female	-4.028	Yes	0.0004	0.0001
3mo. old:healthy female vs. D10:healthy male	-4.710	Yes	0.0001	<0.0001
3mo. old:healthy female vs. D10:healthy female	-4.034	Yes	0.0004	0.0001
3mo. old:healthy female vs. D10:cystic male	-2.524	Yes	0.0154	0.0097
3mo. old:healthy female vs. D10:cystic female	-1.338	No	0.1778	0.1911
3mo. old:healthy female vs. D22:healthy male	-1.114	No	0.2297	0.2760
3mo. old:healthy female vs. D22:healthy female	-1.682	No	0.1081	0.1010
3mo. old:healthy female vs. D22:cystic male	-3.112	Yes	0.0021	0.0009
3mo. old:healthy female vs. D22:cystic female	-0.9778	No	0.2688	0.3386
3mo. old:healthy female vs. D42:healthy male	-3.200	Yes	0.0075	0.0042
3mo. old:healthy female vs. D42:healthy female	-2.338	Yes	0.0235	0.0165
3mo. old:healthy female vs. D42:cystic male	0.4079	No	0.4354	0.6581
3mo. old:healthy female vs. D42:cystic female	0.07022	No	0.5680	0.9451
3mo. old:healthy female vs. D90:healthy male	-0.9049	No	0.2622	0.3270

3mo. old:healthy female vs. D90:healthy female	0.03222	No	0.5780	0.9766
3mo. old:healthy female vs. D90:cystic male	-0.9400	No	0.2297	0.2766
3mo. old:healthy female vs. D90:cystic female	-0.4078	No	0.4296	0.6464
3mo. old:cystic male vs. 3mo. old:cystic female	0.8055	No	0.3257	0.4403
3mo. old:cystic male vs. D3:healthy male	-6.701	Yes	<0.0001	<0.0001
3mo. old:cystic male vs. D3:healthy female	-5.713	Yes	<0.0001	<0.0001
3mo. old:cystic male vs. D3:cystic male	-5.243	Yes	<0.0001	<0.0001
3mo. old:cystic male vs. D3:cystic female	-4.332	Yes	0.0007	0.0003
3mo. old:cystic male vs. D10:healthy male	-5.015	Yes	0.0003	<0.0001
3mo. old:cystic male vs. D10:healthy female	-4.338	Yes	0.0007	0.0003
3mo. old:cystic male vs. D10:cystic male	-2.829	Yes	0.0179	0.0116
3mo. old:cystic male vs. D10:cystic female	-1.642	No	0.1552	0.1573
3mo. old:cystic male vs. D22:healthy male	-1.418	No	0.1967	0.2215
3mo. old:cystic male vs. D22:healthy female	-1.986	No	0.0971	0.0877
3mo. old:cystic male vs. D22:cystic male	-3.416	Yes	0.0036	0.0017
3mo. old:cystic male vs. D22:cystic female	-1.282	No	0.2266	0.2688
3mo. old:cystic male vs. D42:healthy male	-3.505	Yes	0.0085	0.0049
3mo. old:cystic male vs. D42:healthy female	-2.642	Yes	0.0256	0.0183
3mo. old:cystic male vs. D42:cystic male	0.1037	No	0.5604	0.9228
3mo. old:cystic male vs. D42:cystic female	-0.2340	No	0.5278	0.8397
3mo. old:cystic male vs. D90:healthy male	-1.209	No	0.2215	0.2600
3mo. old:cystic male vs. D90:healthy female	-0.2720	No	0.5263	0.8245
3mo. old:cystic male vs. D90:cystic male	-1.244	No	0.1967	0.2239
3mo. old:cystic male vs. D90:cystic female	-0.7120	No	0.3540	0.4950
3mo. old:cystic female vs. D3:healthy male	-7.506	Yes	<0.0001	<0.0001
3mo. old:cystic female vs. D3:healthy female	-6.519	Yes	<0.0001	<0.0001
3mo. old:cystic female vs. D3:cystic male	-6.049	Yes	<0.0001	<0.0001
3mo. old:cystic female vs. D3:cystic female	-5.138	Yes	<0.0001	<0.0001
3mo. old:cystic female vs. D10:healthy male	-5.820	Yes	<0.0001	<0.0001
3mo. old:cystic female vs. D10:healthy female	-5.144	Yes	<0.0001	<0.0001
3mo. old:cystic female vs. D10:cystic male	-3.634	Yes	0.0009	0.0003
3mo. old:cystic female vs. D10:cystic female	-2.448	Yes	0.0274	0.0202
3mo. old:cystic female vs. D22:healthy male	-2.224	Yes	0.0435	0.0345
3mo. old:cystic female vs. D22:healthy female	-2.792	Yes	0.0136	0.0083
3mo. old:cystic female vs. D22:cystic male	-4.222	Yes	<0.0001	<0.0001
3mo. old:cystic female vs. D22:cystic female	-2.088	No	0.0557	0.0469
3mo. old:cystic female vs. D42:healthy male	-4.310	Yes	0.0006	0.0002
3mo. old:cystic female vs. D42:healthy female	-3.448	Yes	0.0016	0.0006
3mo. old:cystic female vs. D42:cystic male	-0.7018	No	0.3349	0.4589
3mo. old:cystic female vs. D42:cystic female	-1.040	No	0.2588	0.3196
3mo. old:cystic female vs. D90:healthy male	-2.015	Yes	0.0436	0.0348
3mo. old:cystic female vs. D90:healthy female	-1.078	No	0.2687	0.3368
3mo. old:cystic female vs. D90:cystic male	-2.050	Yes	0.0294	0.0224
3mo. old:cystic female vs. D90:cystic female	-1.518	No	0.1070	0.0988
D3:healthy male vs. D3:healthy female	0.9872	No	0.2084	0.2395
D3:healthy male vs. D3:cystic male	1.457	No	0.0931	0.0835
D3:healthy male vs. D3:cystic female	2.369	Yes	0.0197	0.0132
D3:healthy male vs. D10:healthy male	1.686	No	0.1089	0.1031
D3:healthy male vs. D10:healthy female	2.363	Yes	0.0198	0.0134
D3:healthy male vs. D10:cystic male	3.872	Yes	<0.0001	<0.0001

D3:healthy male vs. D10:cystic female	5.059	Yes	<0.0001	<0.0001
D3:healthy male vs. D22:healthy male	5.283	Yes	<0.0001	<0.0001
D3:healthy male vs. D22:healthy female	4.715	Yes	<0.0001	<0.0001
D3:healthy male vs. D22:cystic male	3.284	Yes	0.0004	0.0001
D3:healthy male vs. D22:cystic female	5.419	Yes	<0.0001	<0.0001
D3:healthy male vs. D42:healthy male	3.196	Yes	0.0047	0.0023
D3:healthy male vs. D42:healthy female	4.059	Yes	<0.0001	<0.0001
D3:healthy male vs. D42:cystic male	6.804	Yes	<0.0001	<0.0001
D3:healthy male vs. D42:cystic female	6.467	Yes	<0.0001	<0.0001
D3:healthy male vs. D90:healthy male	5.492	Yes	<0.0001	<0.0001
D3:healthy male vs. D90:healthy female	6.429	Yes	<0.0001	<0.0001
D3:healthy male vs. D90:cystic male	5.456	Yes	<0.0001	<0.0001
D3:healthy male vs. D90:cystic female	5.989	Yes	<0.0001	<0.0001
D3:healthy female vs. D3:cystic male	0.4700	No	0.4225	0.6308
D3:healthy female vs. D3:cystic female	1.381	No	0.1825	0.1984
D3:healthy female vs. D10:healthy male	0.6989	No	0.3809	0.5423
D3:healthy female vs. D10:healthy female	1.375	No	0.1832	0.2004
D3:healthy female vs. D10:cystic male	2.885	Yes	0.0090	0.0052
D3:healthy female vs. D10:cystic female	4.071	Yes	0.0006	0.0002
D3:healthy female vs. D22:healthy male	4.295	Yes	0.0003	<0.0001
D3:healthy female vs. D22:healthy female	3.727	Yes	0.0016	0.0007
D3:healthy female vs. D22:cystic male	2.297	Yes	0.0274	0.0200
D3:healthy female vs. D22:cystic female	4.431	Yes	0.0002	<0.0001
D3:healthy female vs. D42:healthy male	2.209	No	0.0643	0.0557
D3:healthy female vs. D42:healthy female	3.071	Yes	0.0057	0.0030
D3:healthy female vs. D42:cystic male	5.817	Yes	<0.0001	<0.0001
D3:healthy female vs. D42:cystic female	5.479	Yes	<0.0001	<0.0001
D3:healthy female vs. D90:healthy male	4.504	Yes	<0.0001	<0.0001
D3:healthy female vs. D90:healthy female	5.441	Yes	<0.0001	<0.0001
D3:healthy female vs. D90:cystic male	4.469	Yes	<0.0001	<0.0001
D3:healthy female vs. D90:cystic female	5.001	Yes	<0.0001	<0.0001
D3:cystic male vs. D3:cystic female	0.9114	No	0.2979	0.3953
D3:cystic male vs. D10:healthy male	0.2289	No	0.5278	0.8417
D3:cystic male vs. D10:healthy female	0.9054	No	0.2988	0.3984
D3:cystic male vs. D10:cystic male	2.415	Yes	0.0262	0.0189
D3:cystic male vs. D10:cystic female	3.601	Yes	0.0022	0.0010
D3:cystic male vs. D22:healthy male	3.825	Yes	0.0012	0.0005
D3:cystic male vs. D22:healthy female	3.257	Yes	0.0055	0.0028
D3:cystic male vs. D22:cystic male	1.827	No	0.0726	0.0633
D3:cystic male vs. D22:cystic female	3.961	Yes	0.0008	0.0003
D3:cystic male vs. D42:healthy male	1.739	No	0.1329	0.1309
D3:cystic male vs. D42:healthy female	2.601	Yes	0.0179	0.0116
D3:cystic male vs. D42:cystic male	5.347	Yes	<0.0001	<0.0001
D3:cystic male vs. D42:cystic female	5.009	Yes	<0.0001	<0.0001
D3:cystic male vs. D90:healthy male	4.034	Yes	0.0002	<0.0001
D3:cystic male vs. D90:healthy female	4.971	Yes	0.0001	<0.0001
D3:cystic male vs. D90:cystic male	3.999	Yes	0.0001	<0.0001
D3:cystic male vs. D90:cystic female	4.531	Yes	<0.0001	<0.0001
D3:cystic female vs. D10:healthy male	-0.6825	No	0.3954	0.5782
D3:cystic female vs. D10:healthy female	-0.00600	No	0.5872	0.9959

D3:cystic female vs. D10:cystic male	1.503	No	0.1666	0.1761
D3:cystic female vs. D10:cystic female	2.690	Yes	0.0282	0.0213
D3:cystic female vs. D22:healthy male	2.914	Yes	0.0193	0.0128
D3:cystic female vs. D22:healthy female	2.346	No	0.0540	0.0442
D3:cystic female vs. D22:cystic male	0.9157	No	0.2976	0.3931
D3:cystic female vs. D22:cystic female	3.050	Yes	0.0148	0.0092
D3:cystic female vs. D42:healthy male	0.8275	No	0.3540	0.5003
D3:cystic female vs. D42:healthy female	1.690	No	0.1325	0.1287
D3:cystic female vs. D42:cystic male	4.436	Yes	0.0002	<0.0001
D3:cystic female vs. D42:cystic female	4.098	Yes	0.0013	0.0005
D3:cystic female vs. D90:healthy male	3.123	Yes	0.0075	0.0041
D3:cystic female vs. D90:healthy female	4.060	Yes	0.0025	0.0012
D3:cystic female vs. D90:cystic male	3.088	Yes	0.0057	0.0029
D3:cystic female vs. D90:cystic female	3.620	Yes	0.0016	0.0007
D10:healthy male vs. D10:healthy female	0.6765	No	0.3960	0.5815
D10:healthy male vs. D10:cystic male	2.186	No	0.0744	0.0658
D10:healthy male vs. D10:cystic female	3.373	Yes	0.0114	0.0067
D10:healthy male vs. D22:healthy male	3.597	Yes	0.0073	0.0039
D10:healthy male vs. D22:healthy female	3.029	Yes	0.0211	0.0147
D10:healthy male vs. D22:cystic male	1.598	No	0.1587	0.1648
D10:healthy male vs. D22:cystic female	3.733	Yes	0.0055	0.0028
D10:healthy male vs. D42:healthy male	1.510	No	0.2091	0.2442
D10:healthy male vs. D42:healthy female	2.373	No	0.0557	0.0461
D10:healthy male vs. D42:cystic male	5.118	Yes	<0.0001	<0.0001
D10:healthy male vs. D42:cystic female	4.781	Yes	0.0005	0.0002
D10:healthy male vs. D90:healthy male	3.805	Yes	0.0025	0.0011
D10:healthy male vs. D90:healthy female	4.743	Yes	0.0009	0.0003
D10:healthy male vs. D90:cystic male	3.770	Yes	0.0018	0.0008
D10:healthy male vs. D90:cystic female	4.303	Yes	0.0006	0.0002
D10:healthy female vs. D10:cystic male	1.509	No	0.1660	0.1744
D10:healthy female vs. D10:cystic female	2.696	Yes	0.0281	0.0210
D10:healthy female vs. D22:healthy male	2.920	Yes	0.0192	0.0126
D10:healthy female vs. D22:healthy female	2.352	No	0.0538	0.0436
D10:healthy female vs. D22:cystic male	0.9217	No	0.2966	0.3900
D10:healthy female vs. D22:cystic female	3.056	Yes	0.0147	0.0091
D10:healthy female vs. D42:healthy male	0.8335	No	0.3540	0.4972
D10:healthy female vs. D42:healthy female	1.696	No	0.1320	0.1273
D10:healthy female vs. D42:cystic male	4.442	Yes	0.0002	<0.0001
D10:healthy female vs. D42:cystic female	4.104	Yes	0.0013	0.0005
D10:healthy female vs. D90:healthy male	3.129	Yes	0.0075	0.0040
D10:healthy female vs. D90:healthy female	4.066	Yes	0.0025	0.0012
D10:healthy female vs. D90:cystic male	3.094	Yes	0.0056	0.0029
D10:healthy female vs. D90:cystic female	3.626	Yes	0.0016	0.0007
D10:cystic male vs. D10:cystic female	1.187	No	0.2342	0.2850
D10:cystic male vs. D22:healthy male	1.411	No	0.1856	0.2041
D10:cystic male vs. D22:healthy female	0.8427	No	0.3278	0.4472
D10:cystic male vs. D22:cystic male	-0.5876	No	0.3888	0.5638
D10:cystic male vs. D22:cystic female	1.547	No	0.1587	0.1641
D10:cystic male vs. D42:healthy male	-0.6758	No	0.3895	0.5672
D10:cystic male vs. D42:healthy female	0.1867	No	0.5340	0.8597

D10:cystic male vs. D42:cystic male	2.932	Yes	0.0081	0.0045
D10:cystic male vs. D42:cystic female	2.595	Yes	0.0274	0.0204
D10:cystic male vs. D90:healthy male	1.620	No	0.1181	0.1132
D10:cystic male vs. D90:healthy female	2.557	Yes	0.0408	0.0318
D10:cystic male vs. D90:cystic male	1.584	No	0.1085	0.1020
D10:cystic male vs. D90:cystic female	2.117	Yes	0.0428	0.0336
D10:cystic female vs. D22:healthy male	0.2240	No	0.5278	0.8464
D10:cystic female vs. D22:healthy female	-0.3440	No	0.4909	0.7662
D10:cystic female vs. D22:cystic male	-1.774	No	0.1070	0.0993
D10:cystic female vs. D22:cystic female	0.3600	No	0.4880	0.7556
D10:cystic female vs. D42:healthy male	-1.863	No	0.1329	0.1306
D10:cystic female vs. D42:healthy female	-1.000	No	0.2846	0.3672
D10:cystic female vs. D42:cystic male	1.746	No	0.1100	0.1048
D10:cystic female vs. D42:cystic female	1.408	No	0.1967	0.2248
D10:cystic female vs. D90:healthy male	0.4329	No	0.4521	0.6861
D10:cystic female vs. D90:healthy female	1.370	No	0.2248	0.2652
D10:cystic female vs. D90:cystic male	0.3978	No	0.4553	0.6966
D10:cystic female vs. D90:cystic female	0.9300	No	0.2878	0.3731
D22:healthy male vs. D22:healthy female	-0.5680	No	0.4193	0.6235
D22:healthy male vs. D22:cystic male	-1.998	No	0.0726	0.0638
D22:healthy male vs. D22:cystic female	0.1360	No	0.5567	0.9064
D22:healthy male vs. D42:healthy male	-2.087	No	0.0998	0.0907
D22:healthy male vs. D42:healthy female	-1.224	No	0.2266	0.2701
D22:healthy male vs. D42:cystic male	1.522	No	0.1552	0.1569
D22:healthy male vs. D42:cystic female	1.184	No	0.2498	0.3070
D22:healthy male vs. D90:healthy male	0.2089	No	0.5278	0.8454
D22:healthy male vs. D90:healthy female	1.146	No	0.2760	0.3510
D22:healthy male vs. D90:cystic male	0.1738	No	0.5351	0.8647
D22:healthy male vs. D90:cystic female	0.7060	No	0.3540	0.4987
D22:healthy female vs. D22:cystic male	-1.430	No	0.1713	0.1831
D22:healthy female vs. D22:cystic female	0.7040	No	0.3809	0.5430
D22:healthy female vs. D42:healthy male	-1.519	No	0.1952	0.2171
D22:healthy female vs. D42:healthy female	-0.6560	No	0.3836	0.5538
D22:healthy female vs. D42:cystic male	2.090	No	0.0613	0.0527
D22:healthy female vs. D42:cystic female	1.752	No	0.1329	0.1315
D22:healthy female vs. D90:healthy male	0.7769	No	0.3375	0.4686
D22:healthy female vs. D90:healthy female	1.714	No	0.1587	0.1639
D22:healthy female vs. D90:cystic male	0.7418	No	0.3375	0.4675
D22:healthy female vs. D90:cystic female	1.274	No	0.1967	0.2230
D22:cystic male vs. D22:cystic female	2.134	No	0.0565	0.0479
D22:cystic male vs. D42:healthy male	-0.08821	No	0.5679	0.9387
D22:cystic male vs. D42:healthy female	0.7743	No	0.3278	0.4471
D22:cystic male vs. D42:cystic male	3.520	Yes	0.0011	0.0004
D22:cystic male vs. D42:cystic female	3.182	Yes	0.0066	0.0035
D22:cystic male vs. D90:healthy male	2.207	Yes	0.0330	0.0253
D22:cystic male vs. D90:healthy female	3.144	Yes	0.0115	0.0068
D22:cystic male vs. D90:cystic male	2.172	Yes	0.0272	0.0197
D22:cystic male vs. D90:cystic female	2.704	Yes	0.0085	0.0049
D22:cystic female vs. D42:healthy male	-2.223	No	0.0806	0.0718
D22:cystic female vs. D42:healthy female	-1.360	No	0.1967	0.2207

D22:cystic female vs. D42:cystic male	1.386	No	0.1822	0.1971
D22:cystic female vs. D42:cystic female	1.048	No	0.2846	0.3656
D22:cystic female vs. D90:healthy male	0.07286	No	0.5680	0.9458
D22:cystic female vs. D90:healthy female	1.010	No	0.3068	0.4109
D22:cystic female vs. D90:cystic male	0.03778	No	0.5780	0.9705
D22:cystic female vs. D90:cystic female	0.5700	No	0.3965	0.5848
D42:healthy male vs. D42:healthy female	0.8625	No	0.3375	0.4654
D42:healthy male vs. D42:cystic male	3.608	Yes	0.0041	0.0020
D42:healthy male vs. D42:cystic female	3.271	Yes	0.0139	0.0085
D42:healthy male vs. D90:healthy male	2.295	No	0.0557	0.0469
D42:healthy male vs. D90:healthy female	3.233	Yes	0.0198	0.0135
D42:healthy male vs. D90:cystic male	2.260	No	0.0513	0.0413
D42:healthy male vs. D90:cystic female	2.793	Yes	0.0199	0.0137
D42:healthy female vs. D42:cystic male	2.746	Yes	0.0129	0.0078
D42:healthy female vs. D42:cystic female	2.408	Yes	0.0402	0.0312
D42:healthy female vs. D90:healthy male	1.433	No	0.1575	0.1606
D42:healthy female vs. D90:healthy female	2.370	No	0.0557	0.0463
D42:healthy female vs. D90:cystic male	1.398	No	0.1484	0.1486
D42:healthy female vs. D90:cystic female	1.930	No	0.0613	0.0524
D42:cystic male vs. D42:cystic female	-0.3377	No	0.4879	0.7525
D42:cystic male vs. D90:healthy male	-1.313	No	0.1700	0.1808
D42:cystic male vs. D90:healthy female	-0.3757	No	0.4838	0.7431
D42:cystic male vs. D90:cystic male	-1.348	No	0.1459	0.1452
D42:cystic male vs. D90:cystic female	-0.8157	No	0.2966	0.3894
D42:cystic female vs. D90:healthy male	-0.9751	No	0.2842	0.3632
D42:cystic female vs. D90:healthy female	-0.03800	No	0.5780	0.9753
D42:cystic female vs. D90:cystic male	-1.010	No	0.2602	0.3229
D42:cystic female vs. D90:cystic female	-0.4780	No	0.4296	0.6467
D90:healthy male vs. D90:healthy female	0.9371	No	0.3078	0.4142
D90:healthy male vs. D90:cystic male	-0.03508	No	0.5780	0.9696
D90:healthy male vs. D90:cystic female	0.4971	No	0.4049	0.5996
D90:healthy female vs. D90:cystic male	-0.9722	No	0.2894	0.3770
D90:healthy female vs. D90:cystic female	-0.4400	No	0.4553	0.6945
D90:cystic male vs. D90:cystic female	0.5322	No	0.3822	0.5495

Supplemental Table 11. Three-way ANOVA for Figure 1D.

Table Analyzed	KRM %TdT+				
Three-way ANOVA	Ordinary				
Alpha	0.05				

Source of Variation	% of total variation	P value	P value summary	Significant?	
Time	51.45	<0.0001	****	Yes	
Genotype	2.329	0.0004	***	Yes	
Sex	9.009	<0.0001	****	Yes	
Time x Genotype	8.746	<0.0001	****	Yes	
Time x Sex	1.557	0.1194	ns	No	
Genotype x Sex	0.09700	0.4567	ns	No	
Time x Genotype x Sex	1.798	0.0737	ns	No	

ANOVA table	SS (Type III)	DF	MS	F (DFn, DFd)	P value
Time	10886	5	2177	F (5, 130) = 59.13	P<0.0001
Genotype	492.6	1	492.6	F (1, 130) = 13.38	P=0.0004
Sex	1906	1	1906	F (1, 130) = 51.77	P<0.0001
Time x Genotype	1850	5	370.1	F (5, 130) = 10.05	P<0.0001
Time x Sex	329.5	5	65.89	F (5, 130) = 1.790	P=0.1194
Genotype x Sex	20.52	1	20.52	F (1, 130) = 0.5574	P=0.4567
Time x Genotype x Sex	380.4	5	76.08	F (5, 130) = 2.066	P=0.0737
Residual	4787	130	36.82		

Supplemental Table 12. Three-way ANOVA post-hoc multiple comparisons test for Figure 1D.

Compare each cell mean with every other cell mean				
Number of families	1			
Number of comparisons per family	276			
Q	0.05			
Two-stage linear step-up procedure of Benjamini, Krieger and Yekutieli	Mean diff.	Discovery?	q value	Individual P Value
3mo. old:healthy male vs. 3mo. old:healthy female	0.3000	No	0.3447	0.9254
3mo. old:healthy male vs. 3mo. old:cystic male	-12.09	Yes	0.0009	0.0013
3mo. old:healthy male vs. 3mo. old:cystic female	-1.117	No	0.2886	0.7338
3mo. old:healthy male vs. D3:healthy male	-9.667	Yes	0.0009	0.0013
3mo. old:healthy male vs. D3:healthy female	-10.85	Yes	0.0012	0.0016
3mo. old:healthy male vs. D3:cystic male	-6.024	Yes	0.0382	0.0767
3mo. old:healthy male vs. D3:cystic female	1.033	No	0.3027	0.7790
3mo. old:healthy male vs. D10:healthy male	-28.92	Yes	<0.0001	<0.0001
3mo. old:healthy male vs. D10:healthy female	-11.91	Yes	0.0011	0.0015
3mo. old:healthy male vs. D10:cystic male	-16.35	Yes	<0.0001	<0.0001
3mo. old:healthy male vs. D10:cystic female	-7.667	Yes	0.0204	0.0389
3mo. old:healthy male vs. D22:healthy male	-29.21	Yes	<0.0001	<0.0001
3mo. old:healthy male vs. D22:healthy female	-20.37	Yes	<0.0001	<0.0001
3mo. old:healthy male vs. D22:cystic male	-29.08	Yes	<0.0001	<0.0001
3mo. old:healthy male vs. D22:cystic female	-24.61	Yes	<0.0001	<0.0001
3mo. old:healthy male vs. D42:healthy male	-29.54	Yes	<0.0001	<0.0001
3mo. old:healthy male vs. D42:healthy female	-23.38	Yes	<0.0001	<0.0001
3mo. old:healthy male vs. D42:cystic male	-15.57	Yes	<0.0001	<0.0001
3mo. old:healthy male vs. D42:cystic female	-7.747	Yes	0.0197	0.0369
3mo. old:healthy male vs. D90:healthy male	-28.62	Yes	<0.0001	<0.0001
3mo. old:healthy male vs. D90:healthy female	-20.12	Yes	<0.0001	<0.0001
3mo. old:healthy male vs. D90:cystic male	-28.96	Yes	<0.0001	<0.0001
3mo. old:healthy male vs. D90:cystic female	-19.15	Yes	<0.0001	<0.0001
3mo. old:healthy female vs. 3mo. old:cystic male	-12.39	Yes	0.0003	0.0004
3mo. old:healthy female vs. 3mo. old:cystic female	-1.417	No	0.2534	0.6317
3mo. old:healthy female vs. D3:healthy male	-9.967	Yes	0.0001	0.0002
3mo. old:healthy female vs. D3:healthy female	-11.15	Yes	0.0003	0.0004
3mo. old:healthy female vs. D3:cystic male	-6.324	Yes	0.0212	0.0406
3mo. old:healthy female vs. D3:cystic female	0.7333	No	0.3171	0.8288
3mo. old:healthy female vs. D10:healthy male	-29.22	Yes	<0.0001	<0.0001
3mo. old:healthy female vs. D10:healthy female	-12.21	Yes	0.0004	0.0004
3mo. old:healthy female vs. D10:cystic male	-16.65	Yes	<0.0001	<0.0001
3mo. old:healthy female vs. D10:cystic female	-7.967	Yes	0.0119	0.0201
3mo. old:healthy female vs. D22:healthy male	-29.51	Yes	<0.0001	<0.0001
3mo. old:healthy female vs. D22:healthy female	-20.67	Yes	<0.0001	<0.0001
3mo. old:healthy female vs. D22:cystic male	-29.38	Yes	<0.0001	<0.0001
3mo. old:healthy female vs. D22:cystic female	-24.91	Yes	<0.0001	<0.0001
3mo. old:healthy female vs. D42:healthy male	-29.84	Yes	<0.0001	<0.0001
3mo. old:healthy female vs. D42:healthy female	-23.68	Yes	<0.0001	<0.0001
3mo. old:healthy female vs. D42:cystic male	-15.87	Yes	<0.0001	<0.0001
3mo. old:healthy female vs. D42:cystic female	-8.047	Yes	0.0114	0.0189
3mo. old:healthy female vs. D90:healthy male	-28.92	Yes	<0.0001	<0.0001

3mo. old:healthy female vs. D90:healthy female	-20.42	Yes	<0.0001	<0.0001
3mo. old:healthy female vs. D90:cystic male	-29.26	Yes	<0.0001	<0.0001
3mo. old:healthy female vs. D90:cystic female	-19.45	Yes	<0.0001	<0.0001
3mo. old:cystic male vs. 3mo. old:cystic female	10.97	Yes	0.0013	0.0019
3mo. old:cystic male vs. D3:healthy male	2.420	No	0.1815	0.4413
3mo. old:cystic male vs. D3:healthy female	1.234	No	0.2878	0.7289
3mo. old:cystic male vs. D3:cystic male	6.063	Yes	0.0446	0.0903
3mo. old:cystic male vs. D3:cystic female	13.12	Yes	0.0007	0.0008
3mo. old:cystic male vs. D10:healthy male	-16.83	Yes	<0.0001	<0.0001
3mo. old:cystic male vs. D10:healthy female	0.1800	No	0.3497	0.9627
3mo. old:cystic male vs. D10:cystic male	-4.263	No	0.1108	0.2480
3mo. old:cystic male vs. D10:cystic female	4.420	No	0.1118	0.2515
3mo. old:cystic male vs. D22:healthy male	-17.12	Yes	<0.0001	<0.0001
3mo. old:cystic male vs. D22:healthy female	-8.280	Yes	0.0177	0.0328
3mo. old:cystic male vs. D22:cystic male	-16.99	Yes	<0.0001	<0.0001
3mo. old:cystic male vs. D22:cystic female	-12.52	Yes	0.0010	0.0014
3mo. old:cystic male vs. D42:healthy male	-17.46	Yes	<0.0001	<0.0001
3mo. old:cystic male vs. D42:healthy female	-11.30	Yes	0.0017	0.0026
3mo. old:cystic male vs. D42:cystic male	-3.480	No	0.1394	0.3292
3mo. old:cystic male vs. D42:cystic female	4.340	No	0.1142	0.2602
3mo. old:cystic male vs. D90:healthy male	-16.54	Yes	<0.0001	<0.0001
3mo. old:cystic male vs. D90:healthy female	-8.030	Yes	0.0256	0.0506
3mo. old:cystic male vs. D90:cystic male	-16.87	Yes	<0.0001	<0.0001
3mo. old:cystic male vs. D90:cystic female	-7.068	Yes	0.0223	0.0431
3mo. old:cystic female vs. D3:healthy male	-8.550	Yes	0.0012	0.0016
3mo. old:cystic female vs. D3:healthy female	-9.736	Yes	0.0016	0.0024
3mo. old:cystic female vs. D3:cystic male	-4.907	No	0.0575	0.1206
3mo. old:cystic female vs. D3:cystic female	2.150	No	0.2174	0.5353
3mo. old:cystic female vs. D10:healthy male	-27.80	Yes	<0.0001	<0.0001
3mo. old:cystic female vs. D10:healthy female	-10.79	Yes	0.0015	0.0022
3mo. old:cystic female vs. D10:cystic male	-15.23	Yes	<0.0001	<0.0001
3mo. old:cystic female vs. D10:cystic female	-6.550	Yes	0.0303	0.0605
3mo. old:cystic female vs. D22:healthy male	-28.09	Yes	<0.0001	<0.0001
3mo. old:cystic female vs. D22:healthy female	-19.25	Yes	<0.0001	<0.0001
3mo. old:cystic female vs. D22:cystic male	-27.96	Yes	<0.0001	<0.0001
3mo. old:cystic female vs. D22:cystic female	-23.49	Yes	<0.0001	<0.0001
3mo. old:cystic female vs. D42:healthy male	-28.43	Yes	<0.0001	<0.0001
3mo. old:cystic female vs. D42:healthy female	-22.27	Yes	<0.0001	<0.0001
3mo. old:cystic female vs. D42:cystic male	-14.45	Yes	<0.0001	<0.0001
3mo. old:cystic female vs. D42:cystic female	-6.630	Yes	0.0289	0.0575
3mo. old:cystic female vs. D90:healthy male	-27.51	Yes	<0.0001	<0.0001
3mo. old:cystic female vs. D90:healthy female	-19.00	Yes	<0.0001	<0.0001
3mo. old:cystic female vs. D90:cystic male	-27.84	Yes	<0.0001	<0.0001
3mo. old:cystic female vs. D90:cystic female	-18.04	Yes	<0.0001	<0.0001
D3:healthy male vs. D3:healthy female	-1.186	No	0.2667	0.6702
D3:healthy male vs. D3:cystic male	3.643	No	0.0890	0.1920
D3:healthy male vs. D3:cystic female	10.70	Yes	0.0007	0.0009
D3:healthy male vs. D10:healthy male	-19.25	Yes	<0.0001	<0.0001
D3:healthy male vs. D10:healthy female	-2.240	No	0.1949	0.4760
D3:healthy male vs. D10:cystic male	-6.683	Yes	0.0138	0.0242

D3:healthy male vs. D10:cystic female	2.000	No	0.2139	0.5244
D3:healthy male vs. D22:healthy male	-19.54	Yes	<0.0001	<0.0001
D3:healthy male vs. D22:healthy female	-10.70	Yes	0.0007	0.0009
D3:healthy male vs. D22:cystic male	-19.41	Yes	<0.0001	<0.0001
D3:healthy male vs. D22:cystic female	-14.94	Yes	<0.0001	<0.0001
D3:healthy male vs. D42:healthy male	-19.88	Yes	<0.0001	<0.0001
D3:healthy male vs. D42:healthy female	-13.72	Yes	<0.0001	<0.0001
D3:healthy male vs. D42:cystic male	-5.900	Yes	0.0191	0.0355
D3:healthy male vs. D42:cystic female	1.920	No	0.2189	0.5411
D3:healthy male vs. D90:healthy male	-18.96	Yes	<0.0001	<0.0001
D3:healthy male vs. D90:healthy female	-10.45	Yes	0.0018	0.0027
D3:healthy male vs. D90:cystic male	-19.29	Yes	<0.0001	<0.0001
D3:healthy male vs. D90:cystic female	-9.488	Yes	0.0004	0.0005
D3:healthy female vs. D3:cystic male	4.829	No	0.0656	0.1390
D3:healthy female vs. D3:cystic female	11.89	Yes	0.0008	0.0011
D3:healthy female vs. D10:healthy male	-18.06	Yes	<0.0001	<0.0001
D3:healthy female vs. D10:healthy female	-1.054	No	0.2993	0.7671
D3:healthy female vs. D10:cystic male	-5.498	No	0.0515	0.1058
D3:healthy female vs. D10:cystic female	3.186	No	0.1561	0.3716
D3:healthy female vs. D22:healthy male	-18.35	Yes	<0.0001	<0.0001
D3:healthy female vs. D22:healthy female	-9.514	Yes	0.0053	0.0084
D3:healthy female vs. D22:cystic male	-18.23	Yes	<0.0001	<0.0001
D3:healthy female vs. D22:cystic female	-13.75	Yes	0.0002	0.0002
D3:healthy female vs. D42:healthy male	-18.69	Yes	<0.0001	<0.0001
D3:healthy female vs. D42:healthy female	-12.53	Yes	0.0003	0.0003
D3:healthy female vs. D42:cystic male	-4.714	No	0.0698	0.1485
D3:healthy female vs. D42:cystic female	3.106	No	0.1605	0.3837
D3:healthy female vs. D90:healthy male	-17.77	Yes	<0.0001	<0.0001
D3:healthy female vs. D90:healthy female	-9.264	Yes	0.0099	0.0162
D3:healthy female vs. D90:cystic male	-18.10	Yes	<0.0001	<0.0001
D3:healthy female vs. D90:cystic female	-8.302	Yes	0.0058	0.0092
D3:cystic male vs. D3:cystic female	7.057	Yes	0.0250	0.0491
D3:cystic male vs. D10:healthy male	-22.89	Yes	<0.0001	<0.0001
D3:cystic male vs. D10:healthy female	-5.883	Yes	0.0490	0.1002
D3:cystic male vs. D10:cystic male	-10.33	Yes	0.0018	0.0027
D3:cystic male vs. D10:cystic female	-1.643	No	0.2576	0.6446
D3:cystic male vs. D22:healthy male	-23.18	Yes	<0.0001	<0.0001
D3:cystic male vs. D22:healthy female	-14.34	Yes	<0.0001	<0.0001
D3:cystic male vs. D22:cystic male	-23.06	Yes	<0.0001	<0.0001
D3:cystic male vs. D22:cystic female	-18.58	Yes	<0.0001	<0.0001
D3:cystic male vs. D42:healthy male	-23.52	Yes	<0.0001	<0.0001
D3:cystic male vs. D42:healthy female	-17.36	Yes	<0.0001	<0.0001
D3:cystic male vs. D42:cystic male	-9.543	Yes	0.0025	0.0039
D3:cystic male vs. D42:cystic female	-1.723	No	0.2532	0.6286
D3:cystic male vs. D90:healthy male	-22.60	Yes	<0.0001	<0.0001
D3:cystic male vs. D90:healthy female	-14.09	Yes	0.0003	0.0003
D3:cystic male vs. D90:cystic male	-22.93	Yes	<0.0001	<0.0001
D3:cystic male vs. D90:cystic female	-13.13	Yes	<0.0001	<0.0001
D3:cystic female vs. D10:healthy male	-29.95	Yes	<0.0001	<0.0001
D3:cystic female vs. D10:healthy female	-12.94	Yes	0.0008	0.0010

D3:cystic female vs. D10:cystic male	-17.38	Yes	<0.0001	<0.0001
D3:cystic female vs. D10:cystic female	-8.700	Yes	0.0142	0.0250
D3:cystic female vs. D22:healthy male	-30.24	Yes	<0.0001	<0.0001
D3:cystic female vs. D22:healthy female	-21.40	Yes	<0.0001	<0.0001
D3:cystic female vs. D22:cystic male	-30.11	Yes	<0.0001	<0.0001
D3:cystic female vs. D22:cystic female	-25.64	Yes	<0.0001	<0.0001
D3:cystic female vs. D42:healthy male	-30.58	Yes	<0.0001	<0.0001
D3:cystic female vs. D42:healthy female	-24.42	Yes	<0.0001	<0.0001
D3:cystic female vs. D42:cystic male	-16.60	Yes	<0.0001	<0.0001
D3:cystic female vs. D42:cystic female	-8.780	Yes	0.0136	0.0238
D3:cystic female vs. D90:healthy male	-29.66	Yes	<0.0001	<0.0001
D3:cystic female vs. D90:healthy female	-21.15	Yes	<0.0001	<0.0001
D3:cystic female vs. D90:cystic male	-29.99	Yes	<0.0001	<0.0001
D3:cystic female vs. D90:cystic female	-20.19	Yes	<0.0001	<0.0001
D10:healthy male vs. D10:healthy female	17.01	Yes	<0.0001	<0.0001
D10:healthy male vs. D10:cystic male	12.57	Yes	0.0012	0.0017
D10:healthy male vs. D10:cystic female	21.25	Yes	<0.0001	<0.0001
D10:healthy male vs. D22:healthy male	-0.2900	No	0.3461	0.9433
D10:healthy male vs. D22:healthy female	8.550	Yes	0.0200	0.0376
D10:healthy male vs. D22:cystic male	-0.1643	No	0.3497	0.9656
D10:healthy male vs. D22:cystic female	4.310	No	0.1246	0.2916
D10:healthy male vs. D42:healthy male	-0.6250	No	0.3344	0.8844
D10:healthy male vs. D42:healthy female	5.533	No	0.0749	0.1601
D10:healthy male vs. D42:cystic male	13.35	Yes	0.0005	0.0006
D10:healthy male vs. D42:cystic female	21.17	Yes	<0.0001	<0.0001
D10:healthy male vs. D90:healthy male	0.2929	No	0.3461	0.9387
D10:healthy male vs. D90:healthy female	8.800	Yes	0.0220	0.0423
D10:healthy male vs. D90:cystic male	-0.03889	No	0.3546	0.9915
D10:healthy male vs. D90:cystic female	9.763	Yes	0.0060	0.0096
D10:healthy female vs. D10:cystic male	-4.443	No	0.1036	0.2287
D10:healthy female vs. D10:cystic female	4.240	No	0.1178	0.2713
D10:healthy female vs. D22:healthy male	-17.30	Yes	<0.0001	<0.0001
D10:healthy female vs. D22:healthy female	-8.460	Yes	0.0160	0.0293
D10:healthy female vs. D22:cystic male	-17.17	Yes	<0.0001	<0.0001
D10:healthy female vs. D22:cystic female	-12.70	Yes	0.0009	0.0012
D10:healthy female vs. D42:healthy male	-17.64	Yes	<0.0001	<0.0001
D10:healthy female vs. D42:healthy female	-11.48	Yes	0.0015	0.0022
D10:healthy female vs. D42:cystic male	-3.660	No	0.1297	0.3049
D10:healthy female vs. D42:cystic female	4.160	No	0.1203	0.2804
D10:healthy female vs. D90:healthy male	-16.72	Yes	<0.0001	<0.0001
D10:healthy female vs. D90:healthy female	-8.210	Yes	0.0235	0.0458
D10:healthy female vs. D90:cystic male	-17.05	Yes	<0.0001	<0.0001
D10:healthy female vs. D90:cystic female	-7.248	Yes	0.0201	0.0381
D10:cystic male vs. D10:cystic female	8.683	Yes	0.0117	0.0196
D10:cystic male vs. D22:healthy male	-12.86	Yes	0.0005	0.0006
D10:cystic male vs. D22:healthy female	-4.017	No	0.1191	0.2763
D10:cystic male vs. D22:cystic male	-12.73	Yes	0.0002	0.0002
D10:cystic male vs. D22:cystic female	-8.257	Yes	0.0148	0.0263
D10:cystic male vs. D42:healthy male	-13.19	Yes	0.0008	0.0010
D10:cystic male vs. D42:healthy female	-7.033	Yes	0.0239	0.0468

D10:cystic male vs. D42:cystic male	0.7833	No	0.3137	0.8169
D10:cystic male vs. D42:cystic female	8.603	Yes	0.0122	0.0207
D10:cystic male vs. D90:healthy male	-12.27	Yes	0.0003	0.0004
D10:cystic male vs. D90:healthy female	-3.767	No	0.1426	0.3380
D10:cystic male vs. D90:cystic male	-12.61	Yes	0.0001	0.0001
D10:cystic male vs. D90:cystic female	-2.804	No	0.1640	0.3937
D10:cystic female vs. D22:healthy male	-21.54	Yes	<0.0001	<0.0001
D10:cystic female vs. D22:healthy female	-12.70	Yes	0.0009	0.0012
D10:cystic female vs. D22:cystic male	-21.41	Yes	<0.0001	<0.0001
D10:cystic female vs. D22:cystic female	-16.94	Yes	<0.0001	<0.0001
D10:cystic female vs. D42:healthy male	-21.88	Yes	<0.0001	<0.0001
D10:cystic female vs. D42:healthy female	-15.72	Yes	<0.0001	<0.0001
D10:cystic female vs. D42:cystic male	-7.900	Yes	0.0154	0.0279
D10:cystic female vs. D42:cystic female	-0.08000	No	0.3530	0.9834
D10:cystic female vs. D90:healthy male	-20.96	Yes	<0.0001	<0.0001
D10:cystic female vs. D90:healthy female	-12.45	Yes	0.0018	0.0027
D10:cystic female vs. D90:cystic male	-21.29	Yes	<0.0001	<0.0001
D10:cystic female vs. D90:cystic female	-11.49	Yes	0.0009	0.0012
D22:healthy male vs. D22:healthy female	8.840	Yes	0.0132	0.0228
D22:healthy male vs. D22:cystic male	0.1257	No	0.3501	0.9718
D22:healthy male vs. D22:cystic female	4.600	No	0.1049	0.2328
D22:healthy male vs. D42:healthy male	-0.3350	No	0.3461	0.9345
D22:healthy male vs. D42:healthy female	5.823	No	0.0558	0.1154
D22:healthy male vs. D42:cystic male	13.64	Yes	0.0002	0.0002
D22:healthy male vs. D42:cystic female	21.46	Yes	<0.0001	<0.0001
D22:healthy male vs. D90:healthy male	0.5829	No	0.3312	0.8699
D22:healthy male vs. D90:healthy female	9.090	Yes	0.0151	0.0272
D22:healthy male vs. D90:cystic male	0.2511	No	0.3461	0.9410
D22:healthy male vs. D90:cystic female	10.05	Yes	0.0028	0.0043
D22:healthy female vs. D22:cystic male	-8.714	Yes	0.0095	0.0155
D22:healthy female vs. D22:cystic female	-4.240	No	0.1178	0.2713
D22:healthy female vs. D42:healthy male	-9.175	Yes	0.0146	0.0259
D22:healthy female vs. D42:healthy female	-3.017	No	0.1706	0.4131
D22:healthy female vs. D42:cystic male	4.800	No	0.0834	0.1791
D22:healthy female vs. D42:cystic female	12.62	Yes	0.0009	0.0013
D22:healthy female vs. D90:healthy male	-8.257	Yes	0.0127	0.0217
D22:healthy female vs. D90:healthy female	0.2500	No	0.3477	0.9511
D22:healthy female vs. D90:cystic male	-8.589	Yes	0.0076	0.0123
D22:healthy female vs. D90:cystic female	1.213	No	0.2878	0.7265
D22:cystic male vs. D22:cystic female	4.474	No	0.0960	0.2102
D22:cystic male vs. D42:healthy male	-0.4607	No	0.3392	0.9038
D22:cystic male vs. D42:healthy female	5.698	Yes	0.0461	0.0939
D22:cystic male vs. D42:cystic male	13.51	Yes	<0.0001	<0.0001
D22:cystic male vs. D42:cystic female	21.33	Yes	<0.0001	<0.0001
D22:cystic male vs. D90:healthy male	0.4571	No	0.3346	0.8881
D22:cystic male vs. D90:healthy female	8.964	Yes	0.0118	0.0199
D22:cystic male vs. D90:cystic male	0.1254	No	0.3497	0.9674
D22:cystic male vs. D90:cystic female	9.927	Yes	0.0014	0.0020
D22:cystic female vs. D42:healthy male	-4.935	No	0.1035	0.2276
D22:cystic female vs. D42:healthy female	1.223	No	0.2897	0.7397

D22:cystic female vs. D42:cystic male	9.040	Yes	0.0075	0.0121
D22:cystic female vs. D42:cystic female	16.86	Yes	<0.0001	<0.0001
D22:cystic female vs. D90:healthy male	-4.017	No	0.1142	0.2603
D22:cystic female vs. D90:healthy female	4.490	No	0.1178	0.2720
D22:cystic female vs. D90:cystic male	-4.349	No	0.0923	0.2011
D22:cystic female vs. D90:cystic female	5.453	No	0.0565	0.1174
D42:healthy male vs. D42:healthy female	6.158	No	0.0567	0.1183
D42:healthy male vs. D42:cystic male	13.98	Yes	0.0003	0.0003
D42:healthy male vs. D42:cystic female	21.80	Yes	<0.0001	<0.0001
D42:healthy male vs. D90:healthy male	0.9179	No	0.3122	0.8097
D42:healthy male vs. D90:healthy female	9.425	Yes	0.0162	0.0298
D42:healthy male vs. D90:cystic male	0.5861	No	0.3312	0.8725
D42:healthy male vs. D90:cystic female	10.39	Yes	0.0038	0.0060
D42:healthy female vs. D42:cystic male	7.817	Yes	0.0129	0.0222
D42:healthy female vs. D42:cystic female	15.64	Yes	<0.0001	<0.0001
D42:healthy female vs. D90:healthy male	-5.240	No	0.0584	0.1230
D42:healthy female vs. D90:healthy female	3.267	No	0.1683	0.4058
D42:healthy female vs. D90:cystic male	-5.572	Yes	0.0416	0.0838
D42:healthy female vs. D90:cystic female	4.229	No	0.0919	0.1992
D42:cystic male vs. D42:cystic female	7.820	Yes	0.0161	0.0295
D42:cystic male vs. D90:healthy male	-13.06	Yes	<0.0001	<0.0001
D42:cystic male vs. D90:healthy female	-4.550	No	0.1049	0.2337
D42:cystic male vs. D90:cystic male	-13.39	Yes	<0.0001	<0.0001
D42:cystic male vs. D90:cystic female	-3.587	No	0.1130	0.2554
D42:cystic female vs. D90:healthy male	-20.88	Yes	<0.0001	<0.0001
D42:cystic female vs. D90:healthy female	-12.37	Yes	0.0019	0.0029
D42:cystic female vs. D90:cystic male	-21.21	Yes	<0.0001	<0.0001
D42:cystic female vs. D90:cystic female	-11.41	Yes	0.0009	0.0013
D90:healthy male vs. D90:healthy female	8.507	Yes	0.0151	0.0270
D90:healthy male vs. D90:cystic male	-0.3317	No	0.3416	0.9138
D90:healthy male vs. D90:cystic female	9.470	Yes	0.0020	0.0031
D90:healthy female vs. D90:cystic male	-8.839	Yes	0.0101	0.0167
D90:healthy female vs. D90:cystic female	0.9625	No	0.3081	0.7960
D90:cystic male vs. D90:cystic female	9.801	Yes	0.0009	0.0012

Supplemental Table 13. Three-way ANOVA for Figure 1E.

Table Analyzed	KRM %Ki67+				
Three-way ANOVA	Ordinary				
Alpha	0.05				
Source of Variation	% of total variation	P value	P value summary	Significant?	
Time	73.80	<0.0001	****	Yes	
Genotype	7.065	<0.0001	****	Yes	
Sex	0.5480	0.0270	*	Yes	
Time x Genotype	1.516	0.0208	*	Yes	
Time x Sex	0.2995	0.7394	ns	No	
Genotype x Sex	0.06084	0.4570	ns	No	
Time x Genotype x Sex	0.1558	0.9205	ns	No	
ANOVA table	SS (Type III)	DF	MS	F (DFn, DFd)	P value
Time	27663	5	5533	F (5, 121) = 135.1	P<0.0001
Genotype	2648	1	2648	F (1, 121) = 64.66	P<0.0001
Sex	205.4	1	205.4	F (1, 121) = 5.015	P=0.0270
Time x Genotype	568.2	5	113.6	F (5, 121) = 2.774	P=0.0208
Time x Sex	112.3	5	22.46	F (5, 121) = 0.5482	P=0.7394
Genotype x Sex	22.81	1	22.81	F (1, 121) = 0.5568	P=0.4570
Time x Genotype x Sex	58.39	5	11.68	F (5, 121) = 0.2851	P=0.9205
Residual	4956	121	40.96		

Supplemental Table 14. Three-way ANOVA post-hoc multiple comparisons test for Figure 1E.

Compare each cell mean with every other cell mean				
Number of families	1			
Number of comparisons per family	276			
Q	0.05			
Two-stage linear step-up procedure of Benjamini, Krieger and Yekutieli	Mean diff.	Discovery?	q value	Individual P Value
3mo. old:healthy male vs. 3mo. old:healthy female	1.601	No	0.2401	0.6359
3mo. old:healthy male vs. 3mo. old:cystic male	-2.491	No	0.2009	0.5215
3mo. old:healthy male vs. 3mo. old:cystic female	-3.030	No	0.1549	0.3825
3mo. old:healthy male vs. D3:healthy male	-37.89	Yes	<0.0001	<0.0001
3mo. old:healthy male vs. D3:healthy female	-36.69	Yes	<0.0001	<0.0001
3mo. old:healthy male vs. D3:cystic male	-47.45	Yes	<0.0001	<0.0001
3mo. old:healthy male vs. D3:cystic female	-44.49	Yes	<0.0001	<0.0001
3mo. old:healthy male vs. D10:healthy male	-14.97	Yes	0.0003	0.0004
3mo. old:healthy male vs. D10:healthy female	-6.425	Yes	0.0480	0.0999
3mo. old:healthy male vs. D10:cystic male	-29.19	Yes	<0.0001	<0.0001
3mo. old:healthy male vs. D10:cystic female	-25.33	Yes	<0.0001	<0.0001
3mo. old:healthy male vs. D22:healthy male	-12.16	Yes	0.0014	0.0021
3mo. old:healthy male vs. D22:healthy female	-8.121	Yes	0.0196	0.0382
3mo. old:healthy male vs. D22:cystic male	-16.95	Yes	<0.0001	<0.0001
3mo. old:healthy male vs. D22:cystic female	-15.61	Yes	<0.0001	<0.0001
3mo. old:healthy male vs. D42:healthy male	-1.881	No	0.2444	0.6497
3mo. old:healthy male vs. D42:healthy female	0.8333	No	0.2930	0.8219
3mo. old:healthy male vs. D42:cystic male	-10.84	Yes	0.0018	0.0029
3mo. old:healthy male vs. D42:cystic female	-12.13	Yes	0.0014	0.0022
3mo. old:healthy male vs. D90:healthy male	0.4938	No	0.3115	0.8899
3mo. old:healthy male vs. D90:healthy female	2.009	No	0.2380	0.6276
3mo. old:healthy male vs. D90:cystic male	-7.400	Yes	0.0156	0.0302
3mo. old:healthy male vs. D90:cystic female	-3.908	No	0.1121	0.2604
3mo. old:healthy female vs. 3mo. old:cystic male	-4.092	No	0.1098	0.2539
3mo. old:healthy female vs. 3mo. old:cystic female	-4.631	No	0.0658	0.1391
3mo. old:healthy female vs. D3:healthy male	-39.49	Yes	<0.0001	<0.0001
3mo. old:healthy female vs. D3:healthy female	-38.29	Yes	<0.0001	<0.0001
3mo. old:healthy female vs. D3:cystic male	-49.05	Yes	<0.0001	<0.0001
3mo. old:healthy female vs. D3:cystic female	-46.09	Yes	<0.0001	<0.0001
3mo. old:healthy female vs. D10:healthy male	-16.57	Yes	<0.0001	<0.0001
3mo. old:healthy female vs. D10:healthy female	-8.026	Yes	0.0139	0.0264
3mo. old:healthy female vs. D10:cystic male	-30.80	Yes	<0.0001	<0.0001
3mo. old:healthy female vs. D10:cystic female	-26.93	Yes	<0.0001	<0.0001
3mo. old:healthy female vs. D22:healthy male	-13.76	Yes	0.0001	0.0002
3mo. old:healthy female vs. D22:healthy female	-9.722	Yes	0.0045	0.0074
3mo. old:healthy female vs. D22:cystic male	-18.55	Yes	<0.0001	<0.0001
3mo. old:healthy female vs. D22:cystic female	-17.21	Yes	<0.0001	<0.0001
3mo. old:healthy female vs. D42:healthy male	-3.482	No	0.1492	0.3671
3mo. old:healthy female vs. D42:healthy female	-0.7678	No	0.2930	0.8203
3mo. old:healthy female vs. D42:cystic male	-12.44	Yes	0.0001	0.0002
3mo. old:healthy female vs. D42:cystic female	-13.73	Yes	0.0002	0.0002
3mo. old:healthy female vs. D90:healthy male	-1.107	No	0.2680	0.7320

3mo. old:healthy female vs. D90:healthy female	0.4081	No	0.3193	0.9157
3mo. old:healthy female vs. D90:cystic male	-9.001	Yes	0.0022	0.0034
3mo. old:healthy female vs. D90:cystic female	-5.509	Yes	0.0383	0.0790
3mo. old:cystic male vs. 3mo. old:cystic female	-0.5383	No	0.3113	0.8830
3mo. old:cystic male vs. D3:healthy male	-35.40	Yes	<0.0001	<0.0001
3mo. old:cystic male vs. D3:healthy female	-34.20	Yes	<0.0001	<0.0001
3mo. old:cystic male vs. D3:cystic male	-44.96	Yes	<0.0001	<0.0001
3mo. old:cystic male vs. D3:cystic female	-42.00	Yes	<0.0001	<0.0001
3mo. old:cystic male vs. D10:healthy male	-12.48	Yes	0.0027	0.0043
3mo. old:cystic male vs. D10:healthy female	-3.934	No	0.1366	0.3330
3mo. old:cystic male vs. D10:cystic male	-26.70	Yes	<0.0001	<0.0001
3mo. old:cystic male vs. D10:cystic female	-22.84	Yes	<0.0001	<0.0001
3mo. old:cystic male vs. D22:healthy male	-9.664	Yes	0.0104	0.0185
3mo. old:cystic male vs. D22:healthy female	-5.630	No	0.0763	0.1668
3mo. old:cystic male vs. D22:cystic male	-14.46	Yes	0.0001	0.0002
3mo. old:cystic male vs. D22:cystic female	-13.12	Yes	0.0010	0.0015
3mo. old:cystic male vs. D42:healthy male	0.6105	No	0.3115	0.8872
3mo. old:cystic male vs. D42:healthy female	3.325	No	0.1570	0.3926
3mo. old:cystic male vs. D42:cystic male	-8.346	Yes	0.0144	0.0278
3mo. old:cystic male vs. D42:cystic female	-9.642	Yes	0.0105	0.0188
3mo. old:cystic male vs. D90:healthy male	2.985	No	0.1694	0.4273
3mo. old:cystic male vs. D90:healthy female	4.501	No	0.1243	0.2966
3mo. old:cystic male vs. D90:cystic male	-4.909	No	0.0781	0.1716
3mo. old:cystic male vs. D90:cystic female	-1.417	No	0.2607	0.6984
3mo. old:cystic female vs. D3:healthy male	-34.86	Yes	<0.0001	<0.0001
3mo. old:cystic female vs. D3:healthy female	-33.66	Yes	<0.0001	<0.0001
3mo. old:cystic female vs. D3:cystic male	-44.42	Yes	<0.0001	<0.0001
3mo. old:cystic female vs. D3:cystic female	-41.46	Yes	<0.0001	<0.0001
3mo. old:cystic female vs. D10:healthy male	-11.94	Yes	0.0018	0.0028
3mo. old:cystic female vs. D10:healthy female	-3.396	No	0.1445	0.3539
3mo. old:cystic female vs. D10:cystic male	-26.17	Yes	<0.0001	<0.0001
3mo. old:cystic female vs. D10:cystic female	-22.30	Yes	<0.0001	<0.0001
3mo. old:cystic female vs. D22:healthy male	-9.126	Yes	0.0081	0.0137
3mo. old:cystic female vs. D22:healthy female	-5.092	No	0.0760	0.1654
3mo. old:cystic female vs. D22:cystic male	-13.92	Yes	<0.0001	<0.0001
3mo. old:cystic female vs. D22:cystic female	-12.58	Yes	0.0006	0.0008
3mo. old:cystic female vs. D42:healthy male	1.149	No	0.2797	0.7699
3mo. old:cystic female vs. D42:healthy female	3.863	No	0.1134	0.2659
3mo. old:cystic female vs. D42:cystic male	-7.808	Yes	0.0110	0.0200
3mo. old:cystic female vs. D42:cystic female	-9.104	Yes	0.0082	0.0139
3mo. old:cystic female vs. D90:healthy male	3.523	No	0.1224	0.2896
3mo. old:cystic female vs. D90:healthy female	5.039	No	0.0891	0.2010
3mo. old:cystic female vs. D90:cystic male	-4.370	No	0.0750	0.1625
3mo. old:cystic female vs. D90:cystic female	-0.8787	No	0.2821	0.7841
D3:healthy male vs. D3:healthy female	1.197	No	0.2735	0.7499
D3:healthy male vs. D3:cystic male	-9.557	Yes	0.0037	0.0061
D3:healthy male vs. D3:cystic female	-6.603	Yes	0.0389	0.0806
D3:healthy male vs. D10:healthy male	22.92	Yes	<0.0001	<0.0001
D3:healthy male vs. D10:healthy female	31.47	Yes	<0.0001	<0.0001
D3:healthy male vs. D10:cystic male	8.696	Yes	0.0073	0.0123

D3:healthy male vs. D10:cystic female	12.56	Yes	0.0008	0.0011
D3:healthy male vs. D22:healthy male	25.74	Yes	<0.0001	<0.0001
D3:healthy male vs. D22:healthy female	29.77	Yes	<0.0001	<0.0001
D3:healthy male vs. D22:cystic male	20.94	Yes	<0.0001	<0.0001
D3:healthy male vs. D22:cystic female	22.28	Yes	<0.0001	<0.0001
D3:healthy male vs. D42:healthy male	36.01	Yes	<0.0001	<0.0001
D3:healthy male vs. D42:healthy female	38.72	Yes	<0.0001	<0.0001
D3:healthy male vs. D42:cystic male	27.05	Yes	<0.0001	<0.0001
D3:healthy male vs. D42:cystic female	25.76	Yes	<0.0001	<0.0001
D3:healthy male vs. D90:healthy male	38.38	Yes	<0.0001	<0.0001
D3:healthy male vs. D90:healthy female	39.90	Yes	<0.0001	<0.0001
D3:healthy male vs. D90:cystic male	30.49	Yes	<0.0001	<0.0001
D3:healthy male vs. D90:cystic female	33.98	Yes	<0.0001	<0.0001
D3:healthy female vs. D3:cystic male	-10.75	Yes	0.0030	0.0048
D3:healthy female vs. D3:cystic female	-7.800	Yes	0.0280	0.0563
D3:healthy female vs. D10:healthy male	21.72	Yes	<0.0001	<0.0001
D3:healthy female vs. D10:healthy female	30.27	Yes	<0.0001	<0.0001
D3:healthy female vs. D10:cystic male	7.499	Yes	0.0242	0.0476
D3:healthy female vs. D10:cystic female	11.36	Yes	0.0036	0.0058
D3:healthy female vs. D22:healthy male	24.54	Yes	<0.0001	<0.0001
D3:healthy female vs. D22:healthy female	28.57	Yes	<0.0001	<0.0001
D3:healthy female vs. D22:cystic male	19.75	Yes	<0.0001	<0.0001
D3:healthy female vs. D22:cystic female	21.08	Yes	<0.0001	<0.0001
D3:healthy female vs. D42:healthy male	34.81	Yes	<0.0001	<0.0001
D3:healthy female vs. D42:healthy female	37.53	Yes	<0.0001	<0.0001
D3:healthy female vs. D42:cystic male	25.86	Yes	<0.0001	<0.0001
D3:healthy female vs. D42:cystic female	24.56	Yes	<0.0001	<0.0001
D3:healthy female vs. D90:healthy male	37.19	Yes	<0.0001	<0.0001
D3:healthy female vs. D90:healthy female	38.70	Yes	<0.0001	<0.0001
D3:healthy female vs. D90:cystic male	29.29	Yes	<0.0001	<0.0001
D3:healthy female vs. D90:cystic female	32.79	Yes	<0.0001	<0.0001
D3:cystic male vs. D3:cystic female	2.954	No	0.1706	0.4320
D3:cystic male vs. D10:healthy male	32.47	Yes	<0.0001	<0.0001
D3:cystic male vs. D10:healthy female	41.02	Yes	<0.0001	<0.0001
D3:cystic male vs. D10:cystic male	18.25	Yes	<0.0001	<0.0001
D3:cystic male vs. D10:cystic female	22.11	Yes	<0.0001	<0.0001
D3:cystic male vs. D22:healthy male	35.29	Yes	<0.0001	<0.0001
D3:cystic male vs. D22:healthy female	39.33	Yes	<0.0001	<0.0001
D3:cystic male vs. D22:cystic male	30.50	Yes	<0.0001	<0.0001
D3:cystic male vs. D22:cystic female	31.83	Yes	<0.0001	<0.0001
D3:cystic male vs. D42:healthy male	45.57	Yes	<0.0001	<0.0001
D3:cystic male vs. D42:healthy female	48.28	Yes	<0.0001	<0.0001
D3:cystic male vs. D42:cystic male	36.61	Yes	<0.0001	<0.0001
D3:cystic male vs. D42:cystic female	35.31	Yes	<0.0001	<0.0001
D3:cystic male vs. D90:healthy male	47.94	Yes	<0.0001	<0.0001
D3:cystic male vs. D90:healthy female	49.46	Yes	<0.0001	<0.0001
D3:cystic male vs. D90:cystic male	40.05	Yes	<0.0001	<0.0001
D3:cystic male vs. D90:cystic female	43.54	Yes	<0.0001	<0.0001
D3:cystic female vs. D10:healthy male	29.52	Yes	<0.0001	<0.0001
D3:cystic female vs. D10:healthy female	38.07	Yes	<0.0001	<0.0001

D3:cystic female vs. D10:cystic male	15.30	Yes	<0.0001	<0.0001
D3:cystic female vs. D10:cystic female	19.16	Yes	<0.0001	<0.0001
D3:cystic female vs. D22:healthy male	32.34	Yes	<0.0001	<0.0001
D3:cystic female vs. D22:healthy female	36.37	Yes	<0.0001	<0.0001
D3:cystic female vs. D22:cystic male	27.55	Yes	<0.0001	<0.0001
D3:cystic female vs. D22:cystic female	28.88	Yes	<0.0001	<0.0001
D3:cystic female vs. D42:healthy male	42.61	Yes	<0.0001	<0.0001
D3:cystic female vs. D42:healthy female	45.33	Yes	<0.0001	<0.0001
D3:cystic female vs. D42:cystic male	33.66	Yes	<0.0001	<0.0001
D3:cystic female vs. D42:cystic female	32.36	Yes	<0.0001	<0.0001
D3:cystic female vs. D90:healthy male	44.99	Yes	<0.0001	<0.0001
D3:cystic female vs. D90:healthy female	46.50	Yes	<0.0001	<0.0001
D3:cystic female vs. D90:cystic male	37.09	Yes	<0.0001	<0.0001
D3:cystic female vs. D90:cystic female	40.59	Yes	<0.0001	<0.0001
D10:healthy male vs. D10:healthy female	8.548	Yes	0.0246	0.0487
D10:healthy male vs. D10:cystic male	-14.22	Yes	0.0004	0.0006
D10:healthy male vs. D10:cystic female	-10.36	Yes	0.0100	0.0173
D10:healthy male vs. D22:healthy male	2.818	No	0.1984	0.5128
D10:healthy male vs. D22:healthy female	6.852	No	0.0540	0.1131
D10:healthy male vs. D22:cystic male	-1.974	No	0.2373	0.6235
D10:healthy male vs. D22:cystic female	-0.6400	No	0.3113	0.8817
D10:healthy male vs. D42:healthy male	13.09	Yes	0.0028	0.0045
D10:healthy male vs. D42:healthy female	15.81	Yes	0.0002	0.0002
D10:healthy male vs. D42:cystic male	4.136	No	0.1267	0.3046
D10:healthy male vs. D42:cystic female	2.840	No	0.1984	0.5095
D10:healthy male vs. D90:healthy male	15.47	Yes	0.0001	0.0002
D10:healthy male vs. D90:healthy female	16.98	Yes	0.0002	0.0003
D10:healthy male vs. D90:cystic male	7.573	Yes	0.0258	0.0512
D10:healthy male vs. D90:cystic female	11.07	Yes	0.0034	0.0056
D10:healthy female vs. D10:cystic male	-22.77	Yes	<0.0001	<0.0001
D10:healthy female vs. D10:cystic female	-18.91	Yes	<0.0001	<0.0001
D10:healthy female vs. D22:healthy male	-5.730	No	0.0743	0.1595
D10:healthy female vs. D22:healthy female	-1.696	No	0.2533	0.6760
D10:healthy female vs. D22:cystic male	-10.52	Yes	0.0036	0.0058
D10:healthy female vs. D22:cystic female	-9.188	Yes	0.0134	0.0250
D10:healthy female vs. D42:healthy male	4.545	No	0.1229	0.2919
D10:healthy female vs. D42:healthy female	7.259	Yes	0.0314	0.0635
D10:healthy female vs. D42:cystic male	-4.412	No	0.1053	0.2413
D10:healthy female vs. D42:cystic female	-5.708	No	0.0747	0.1610
D10:healthy female vs. D90:healthy male	6.919	Yes	0.0330	0.0673
D10:healthy female vs. D90:healthy female	8.435	Yes	0.0259	0.0518
D10:healthy female vs. D90:cystic male	-0.9747	No	0.2821	0.7853
D10:healthy female vs. D90:cystic female	2.517	No	0.1925	0.4916
D10:cystic male vs. D10:cystic female	3.861	No	0.1267	0.3049
D10:cystic male vs. D22:healthy male	17.04	Yes	<0.0001	<0.0001
D10:cystic male vs. D22:healthy female	21.07	Yes	<0.0001	<0.0001
D10:cystic male vs. D22:cystic male	12.25	Yes	0.0004	0.0005
D10:cystic male vs. D22:cystic female	13.58	Yes	0.0003	0.0004
D10:cystic male vs. D42:healthy male	27.31	Yes	<0.0001	<0.0001
D10:cystic male vs. D42:healthy female	30.03	Yes	<0.0001	<0.0001

D10:cystic male vs. D42:cystic male	18.36	Yes	<0.0001	<0.0001
D10:cystic male vs. D42:cystic female	17.06	Yes	<0.0001	<0.0001
D10:cystic male vs. D90:healthy male	29.69	Yes	<0.0001	<0.0001
D10:cystic male vs. D90:healthy female	31.20	Yes	<0.0001	<0.0001
D10:cystic male vs. D90:cystic male	21.79	Yes	<0.0001	<0.0001
D10:cystic male vs. D90:cystic female	25.29	Yes	<0.0001	<0.0001
D10:cystic female vs. D22:healthy male	13.18	Yes	0.0010	0.0015
D10:cystic female vs. D22:healthy female	17.21	Yes	<0.0001	<0.0001
D10:cystic female vs. D22:cystic male	8.386	Yes	0.0142	0.0271
D10:cystic female vs. D22:cystic female	9.720	Yes	0.0102	0.0179
D10:cystic female vs. D42:healthy male	23.45	Yes	<0.0001	<0.0001
D10:cystic female vs. D42:healthy female	26.17	Yes	<0.0001	<0.0001
D10:cystic female vs. D42:cystic male	14.50	Yes	0.0001	0.0002
D10:cystic female vs. D42:cystic female	13.20	Yes	0.0010	0.0014
D10:cystic female vs. D90:healthy male	25.83	Yes	<0.0001	<0.0001
D10:cystic female vs. D90:healthy female	27.34	Yes	<0.0001	<0.0001
D10:cystic female vs. D90:cystic male	17.93	Yes	<0.0001	<0.0001
D10:cystic female vs. D90:cystic female	21.43	Yes	<0.0001	<0.0001
D22:healthy male vs. D22:healthy female	4.034	No	0.1328	0.3209
D22:healthy male vs. D22:cystic male	-4.792	No	0.0896	0.2034
D22:healthy male vs. D22:cystic female	-3.458	No	0.1571	0.3946
D22:healthy male vs. D42:healthy male	10.27	Yes	0.0104	0.0182
D22:healthy male vs. D42:healthy female	12.99	Yes	0.0008	0.0011
D22:healthy male vs. D42:cystic male	1.318	No	0.2677	0.7257
D22:healthy male vs. D42:cystic female	0.02200	No	0.3447	0.9957
D22:healthy male vs. D90:healthy male	12.65	Yes	0.0007	0.0010
D22:healthy male vs. D90:healthy female	14.16	Yes	0.0009	0.0013
D22:healthy male vs. D90:cystic male	4.755	No	0.0835	0.1853
D22:healthy male vs. D90:cystic female	8.247	Yes	0.0137	0.0256
D22:healthy female vs. D22:cystic male	-8.826	Yes	0.0110	0.0201
D22:healthy female vs. D22:cystic female	-7.492	Yes	0.0328	0.0666
D22:healthy female vs. D42:healthy male	6.241	No	0.0700	0.1487
D22:healthy female vs. D42:healthy female	8.955	Yes	0.0123	0.0225
D22:healthy female vs. D42:cystic male	-2.716	No	0.1848	0.4699
D22:healthy female vs. D42:cystic female	-4.012	No	0.1333	0.3236
D22:healthy female vs. D90:healthy male	8.615	Yes	0.0125	0.0232
D22:healthy female vs. D90:healthy female	10.13	Yes	0.0110	0.0199
D22:healthy female vs. D90:cystic male	0.7213	No	0.2984	0.8402
D22:healthy female vs. D90:cystic female	4.213	No	0.1088	0.2505
D22:cystic male vs. D22:cystic female	1.334	No	0.2675	0.7224
D22:cystic male vs. D42:healthy male	15.07	Yes	0.0002	0.0003
D22:cystic male vs. D42:healthy female	17.78	Yes	<0.0001	<0.0001
D22:cystic male vs. D42:cystic male	6.110	Yes	0.0373	0.0766
D22:cystic male vs. D42:cystic female	4.814	No	0.0891	0.2014
D22:cystic male vs. D90:healthy male	17.44	Yes	<0.0001	<0.0001
D22:cystic male vs. D90:healthy female	18.96	Yes	<0.0001	<0.0001
D22:cystic male vs. D90:cystic male	9.548	Yes	0.0023	0.0037
D22:cystic male vs. D90:cystic female	13.04	Yes	0.0001	0.0001
D22:cystic female vs. D42:healthy male	13.73	Yes	0.0012	0.0018
D22:cystic female vs. D42:healthy female	16.45	Yes	<0.0001	<0.0001

D22:cystic female vs. D42:cystic male	4.776	No	0.0898	0.2050
D22:cystic female vs. D42:cystic female	3.480	No	0.1570	0.3916
D22:cystic female vs. D90:healthy male	16.11	Yes	<0.0001	<0.0001
D22:cystic female vs. D90:healthy female	17.62	Yes	<0.0001	<0.0001
D22:cystic female vs. D90:cystic male	8.213	Yes	0.0125	0.0231
D22:cystic female vs. D90:cystic female	11.71	Yes	0.0011	0.0017
D42:healthy male vs. D42:healthy female	2.714	No	0.1984	0.5124
D42:healthy male vs. D42:cystic male	-8.957	Yes	0.0143	0.0274
D42:healthy male vs. D42:cystic female	-10.25	Yes	0.0104	0.0185
D42:healthy male vs. D90:healthy male	2.375	No	0.2130	0.5550
D42:healthy male vs. D90:healthy female	3.890	No	0.1570	0.3917
D42:healthy male vs. D90:cystic male	-5.519	No	0.0721	0.1538
D42:healthy male vs. D90:cystic female	-2.028	No	0.2316	0.6059
D42:healthy female vs. D42:cystic male	-11.67	Yes	0.0009	0.0014
D42:healthy female vs. D42:cystic female	-12.97	Yes	0.0008	0.0011
D42:healthy female vs. D90:healthy male	-0.3395	No	0.3211	0.9242
D42:healthy female vs. D90:healthy female	1.176	No	0.2810	0.7764
D42:healthy female vs. D90:cystic male	-8.233	Yes	0.0093	0.0161
D42:healthy female vs. D90:cystic female	-4.742	No	0.0782	0.1726
D42:cystic male vs. D42:cystic female	-1.296	No	0.2680	0.7301
D42:cystic male vs. D90:healthy male	11.33	Yes	0.0009	0.0012
D42:cystic male vs. D90:healthy female	12.85	Yes	0.0012	0.0017
D42:cystic male vs. D90:cystic male	3.438	No	0.1224	0.2886
D42:cystic male vs. D90:cystic female	6.929	Yes	0.0197	0.0385
D42:cystic female vs. D90:healthy male	12.63	Yes	0.0007	0.0010
D42:cystic female vs. D90:healthy female	14.14	Yes	0.0009	0.0013
D42:cystic female vs. D90:cystic male	4.733	No	0.0836	0.1873
D42:cystic female vs. D90:cystic female	8.225	Yes	0.0138	0.0260
D90:healthy male vs. D90:healthy female	1.515	No	0.2626	0.7063
D90:healthy male vs. D90:cystic male	-7.894	Yes	0.0092	0.0158
D90:healthy male vs. D90:cystic female	-4.402	No	0.0836	0.1863
D90:healthy female vs. D90:cystic male	-9.409	Yes	0.0092	0.0159
D90:healthy female vs. D90:cystic female	-5.918	No	0.0635	0.1337
D90:cystic male vs. D90:cystic female	3.492	No	0.1130	0.2637

Supplemental Table 15. Two-way ANOVA for Figure 2B.

Table Analyzed	%macrophage area over nuclear area				
Two-way ANOVA	Ordinary				
Alpha	0.05				
Source of Variation	% of total variation	P value	P value summary	Significant?	
Interaction	1.708	0.8832	ns	No	
Timepoint	16.11	0.0101	*	Yes	
Sex	7.794	0.0063	**	Yes	
ANOVA table	SS (Type III)	DF	MS	F (DFn, DFd)	P value
Interaction	0.5885	5	0.1177	F (5, 75) = 0.3461	P=0.8832
Timepoint	5.549	5	1.110	F (5, 75) = 3.264	P=0.0101
Sex	2.685	1	2.685	F (1, 75) = 7.897	P=0.0063
Residual	25.50	75	0.3400		
Difference between column means					
Predicted (LS) mean of cystic male	0.9807				
Predicted (LS) mean of cystic female	0.6010				
Difference between predicted means	0.3797				
SE of difference	0.1351				
95% CI of difference	0.1105 to 0.6489				
Data summary					
Number of columns (Sex)	2				
Number of rows (Timepoint)	6				
Number of values	87				

Supplemental Table 16. Two-way ANOVA post-hoc multiple comparisons test for Figure 2B.

Compare cell means with others in its row and its column					
Number of families	8				
Number of comparisons per row family	1				
Number of comparisons per column family	15				
Alpha	0.05				
Tukey's multiple comparisons test	Predicted (LS) Mean diff.	95.00% CI of diff.	Below threshold?	Summary	Adjusted P Value
3mo. old					
cystic male vs. cystic female	0.2606	-0.3603 to 0.8816	No	ns	0.4057
D3					
cystic male vs. cystic female	0.2188	-0.3210 to 0.7585	No	ns	0.4220
D10					
cystic male vs. cystic female	0.4643	-0.3373 to 1.266	No	ns	0.2522
D22					
cystic male vs. cystic female	0.6163	-0.002030 to 1.235	No	ns	0.0507
D42					
cystic male vs. cystic female	0.1779	-0.6014 to 0.9571	No	ns	0.6506
D90					
cystic male vs. cystic female	0.5406	-0.006987 to 1.088	No	ns	0.0529
cystic male					
3mo. old vs. D3	0.4763	-0.3483 to 1.301	No	ns	0.5430
3mo. old vs. D10	0.6896	-0.2220 to 1.601	No	ns	0.2444
3mo. old vs. D22	0.2699	-0.5412 to 1.081	No	ns	0.9250
3mo. old vs. D42	0.8849	-0.1841 to 1.954	No	ns	0.1623
3mo. old vs. D90	0.1523	-0.7071 to 1.012	No	ns	0.9953
D3 vs. D10	0.2133	-0.6113 to 1.038	No	ns	0.9738
D3 vs. D22	-0.2064	-0.9182 to 0.5055	No	ns	0.9574
D3 vs. D42	0.4086	-0.5872 to 1.404	No	ns	0.8355
D3 vs. D90	-0.3240	-1.090 to 0.4426	No	ns	0.8178
D10 vs. D22	-0.4197	-1.231 to 0.3914	No	ns	0.6569
D10 vs. D42	0.1953	-0.8737 to 1.264	No	ns	0.9946
D10 vs. D90	-0.5373	-1.397 to 0.3222	No	ns	0.4543
D22 vs. D42	0.6149	-0.3697 to 1.600	No	ns	0.4553
D22 vs. D90	-0.1176	-0.8696 to 0.6344	No	ns	0.9974
D42 vs. D90	-0.7325	-1.757 to 0.2923	No	ns	0.3034
cystic female					
3mo. old vs. D3	0.4344	-0.4482 to 1.317	No	ns	0.7030
3mo. old vs. D10	0.8933	-0.2836 to 2.070	No	ns	0.2411
3mo. old vs. D22	0.6256	-0.3730 to 1.624	No	ns	0.4518
3mo. old vs. D42	0.8021	-0.1965 to 1.801	No	ns	0.1879
3mo. old vs. D90	0.4323	-0.4272 to 1.292	No	ns	0.6834
D3 vs. D10	0.4589	-0.6957 to 1.613	No	ns	0.8531
D3 vs. D22	0.1912	-0.7811 to 1.163	No	ns	0.9924
D3 vs. D42	0.3677	-0.6046 to 1.340	No	ns	0.8774
D3 vs. D90	-0.002100	-0.8308 to 0.8266	No	ns	>0.9999

D10 vs. D22	-0.2677	-1.513 to 0.9778	No	ns	0.9885
D10 vs. D42	-0.09115	-1.337 to 1.154	No	ns	>0.9999
D10 vs. D90	-0.4610	-1.598 to 0.6760	No	ns	0.8423
D22 vs. D42	0.1765	-0.9021 to 1.255	No	ns	0.9968
D22 vs. D90	-0.1933	-1.145 to 0.7580	No	ns	0.9911
D42 vs. D90	-0.3698	-1.321 to 0.5814	No	ns	0.8643

Supplemental Table 17. Two-way ANOVA for Figure 2C.

Table Analyzed	%TdT+ F4/80+ double positive cells				
Two-way ANOVA	Ordinary				
Alpha	0.05				
Source of Variation	% of total variation	P value	P value summary	Significant?	
Interaction	8.149	0.1050	ns	No	
Timepoint	18.64	0.0016	**	Yes	
Sex	8.303	0.0027	**	Yes	
ANOVA table	SS (Type III)	DF	MS	F (DFn, DFd)	P value
Interaction	1459	5	291.8	F (5, 76) = 1.895	P=0.1050
Timepoint	3339	5	667.7	F (5, 76) = 4.337	P=0.0016
Sex	1487	1	1487	F (1, 76) = 9.656	P=0.0027
Residual	11702	76	154.0		
Difference between column means					
Predicted (LS) mean of cystic male	29.90				
Predicted (LS) mean of cystic female	21.18				
Difference between predicted means	8.716				
SE of difference	2.805				
95% CI of difference	3.129 to 14.30				
Data summary					
Number of columns (Sex)	2				
Number of rows (Timepoint)	6				
Number of values	88				

Supplemental Table 18. Two-way ANOVA post-hoc multiple comparisons test for Figure 2C.

Compare cell means with others in its row and its column					
Number of families	8				
Number of comparisons per row family	1				
Number of comparisons per column family	15				
Alpha	0.05				
Tukey's multiple comparisons test	Predicted (LS) Mean diff.	95.00% CI of diff.	Below threshold?	Summary	Adjusted P Value
3mo. old					
cystic male vs. cystic female	13.84	-0.6275 to 28.31	No	ns	0.0605
D3					
cystic male vs. cystic female	-5.133	-16.62 to 6.350	No	ns	0.3761
D10					
cystic male vs. cystic female	7.644	-7.490 to 22.78	No	ns	0.3176
D22					
cystic male vs. cystic female	3.998	-9.157 to 17.15	No	ns	0.5468
D42					
cystic male vs. cystic female	14.56	-1.073 to 30.19	No	ns	0.0675
D90					
cystic male vs. cystic female	17.38	5.735 to 29.04	Yes	**	0.0040
cystic male					
3mo. old vs. D3	11.92	-7.647 to 31.49	No	ns	0.4839
3mo. old vs. D10	4.777	-15.91 to 25.46	No	ns	0.9842
3mo. old vs. D22	3.981	-15.33 to 23.29	No	ns	0.9905
3mo. old vs. D42	-11.55	-34.50 to 11.39	No	ns	0.6827
3mo. old vs. D90	-14.62	-34.85 to 5.616	No	ns	0.2922
D3 vs. D10	-7.144	-24.00 to 9.713	No	ns	0.8162
D3 vs. D22	-7.940	-23.08 to 7.204	No	ns	0.6444
D3 vs. D42	-23.47	-43.04 to -3.907	Yes	**	0.0096
D3 vs. D90	-26.54	-42.85 to -10.23	Yes	***	0.0001
D10 vs. D22	-0.7954	-17.35 to 15.76	No	ns	>0.9999
D10 vs. D42	-16.33	-37.01 to 4.352	No	ns	0.2035
D10 vs. D90	-19.40	-37.02 to -1.768	Yes	*	0.0226
D22 vs. D42	-15.54	-34.85 to 3.776	No	ns	0.1867
D22 vs. D90	-18.60	-34.60 to -2.603	Yes	*	0.0133
D42 vs. D90	-3.065	-23.30 to 17.17	No	ns	0.9978
cystic female					
3mo. old vs. D3	-7.056	-25.83 to 11.72	No	ns	0.8804
3mo. old vs. D10	-1.423	-24.16 to 21.32	No	ns	>0.9999
3mo. old vs. D22	-5.864	-27.11 to 15.38	No	ns	0.9654
3mo. old vs. D42	-10.84	-32.08 to 10.40	No	ns	0.6704
3mo. old vs. D90	-11.08	-29.36 to 7.205	No	ns	0.4900
D3 vs. D10	5.633	-16.58 to 27.85	No	ns	0.9760
D3 vs. D22	1.192	-19.49 to 21.87	No	ns	>0.9999
D3 vs. D42	-3.784	-24.47 to 16.90	No	ns	0.9946
D3 vs. D90	-4.022	-21.65 to 13.61	No	ns	0.9850

D10 vs. D22	-4.441	-28.78 to 19.90	No	ns	0.9946
D10 vs. D42	-9.417	-33.75 to 14.92	No	ns	0.8668
D10 vs. D90	-9.655	-31.46 to 12.15	No	ns	0.7870
D22 vs. D42	-4.976	-27.92 to 17.97	No	ns	0.9881
D22 vs. D90	-5.214	-25.45 to 15.02	No	ns	0.9743
D42 vs. D90	-0.2380	-20.47 to 20.00	No	ns	>0.9999

Supplemental Table 19. One-way ANOVA and post-hoc multiple comparisons test for Figure 2E.

Table Analyzed	D90 cystic males %mac area over nuclear area				
Data sets analyzed	A-C				
Distribution assumption	Normal (Gaussian)				
ANOVA summary					
F	0.4494				
P value	0.6435				
P value summary	ns				
Significant diff. among means (P < 0.05)?	No				
R squared	0.03761				
Brown-Forsythe test					
F (DFn, DFd)	0.9316 (2, 23)				
P value	0.4083				
P value summary	ns				
Are SDs significantly different (P < 0.05)?	No				
Bartlett's test					
Bartlett's statistic (corrected)	4.235				
P value	0.1203				
P value summary	ns				
Are SDs significantly different (P < 0.05)?	No				
ANOVA table	SS	DF	MS	F (DFn, DFd)	P value
Treatment (between columns)	0.4103	2	0.2052	F (2, 23) = 0.4494	P=0.6435
Residual (within columns)	10.50	23	0.4565		
Total	10.91	25			
Data summary					
Number of treatments (columns)	3				
Number of values (total)	26				

Number of families	1								
Number of comparisons per family	3								
Alpha	0.05								
Tukey's multiple comparisons test	Mean diff.	95.00% CI of diff.	Below threshold?	Summary	Adjusted P Value				
LV vs. LC	-0.1610	-0.9384 to 0.6164	No	ns	0.8631	A-B			
LV vs. 3 mo. old	-0.3133	-1.147 to 0.5205	No	ns	0.6204	A-C			
LC vs. 3 mo. old	-0.1523	-1.005 to 0.7004	No	ns	0.8960	B-C			
Test details	Mean 1	Mean 2	Mean diff.	SE of diff.	n1	n2	q	DF	
LV vs. LC	1.080	1.241	-0.1610	0.3104	10	9	0.7335	23	
LV vs. 3 mo. old	1.080	1.393	-0.3133	0.3330	10	7	1.331	23	
LC vs. 3 mo. old	1.241	1.393	-0.1523	0.3405	9	7	0.6327	23	
Compact letter display									
3 mo. old	A								
LC	A								
LV	A								

Table Analyzed	D90 cystic females %mac area over nuclear area				
Data sets analyzed	A-C				
Distribution assumption	Normal (Gaussian)				
ANOVA summary					
F	3.861				
P value	0.0358				
P value summary	*				
Significant diff. among means (P < 0.05)?	Yes				
R squared	0.2513				
Brown-Forsythe test					
F (DFn, DFd)	3.941 (2, 23)				
P value	0.0337				
P value summary	*				
Are SDs significantly different (P < 0.05)?	Yes				
Bartlett's test					
Bartlett's statistic (corrected)	5.804				
P value	0.0549				
P value summary	ns				
Are SDs significantly different (P < 0.05)?	No				
ANOVA table	SS	DF	MS	F (DFn, DFd)	P value
Treatment (between columns)	4.180	2	2.090	F (2, 23) = 3.861	P=0.0358
Residual (within columns)	12.45	23	0.5413		
Total	16.63	25			
Data summary					
Number of treatments (columns)	3				
Number of values (total)	26				

Number of families	1							
Number of comparisons per family	3							
Alpha	0.05							
Tukey's multiple comparisons test	Mean diff.	95.00% CI of diff.	Below threshold?	Summary	Adjusted P Value			
LV vs. LC	0.9372	0.09060 to 1.784	Yes	*	0.0281	A-B		
LV vs. 3 mo. old	0.5049	-0.4031 to 1.413	No	ns	0.3612	A-C		
LC vs. 3 mo. old	-0.4323	-1.361 to 0.4962	No	ns	0.4848	B-C		
Test details	Mean 1	Mean 2	Mean diff.	SE of diff.	n1	n2	q	DF
LV vs. LC	1.637	0.7000	0.9372	0.3381	10	9	3.921	23
LV vs. 3 mo. old	1.637	1.132	0.5049	0.3626	10	7	1.969	23
LC vs. 3 mo. old	0.7000	1.132	-0.4323	0.3708	9	7	1.649	23
Compact letter display								
LV	A							
3 mo. old	A B							
LC	B							

Supplemental Table 20. One-way ANOVA and post-hoc multiple comparisons test for Figure 2F.

Table Analyzed	D90 cystic males %macs that are cyst localized				
Data sets analyzed	A-C				
Distribution assumption	Normal (Gaussian)				
ANOVA summary					
F	1.512				
P value	0.2417				
P value summary	ns				
Significant diff. among means (P < 0.05)?	No				
R squared	0.1162				
Brown-Forsythe test					
F (DFn, DFd)	1.743 (2, 23)				
P value	0.1973				
P value summary	ns				
Are SDs significantly different (P < 0.05)?	No				
Bartlett's test					
Bartlett's statistic (corrected)	7.195				
P value	0.0274				
P value summary	*				
Are SDs significantly different (P < 0.05)?	Yes				
ANOVA table	SS	DF	MS	F (DFn, DFd)	P value
Treatment (between columns)	95.27	2	47.64	F (2, 23) = 1.512	P=0.2417
Residual (within columns)	724.8	23	31.51		
Total	820.1	25			
Data summary					
Number of treatments (columns)	3				
Number of values (total)	26				

Number of families	1							
Number of comparisons per family	3							
Alpha	0.05							
Tukey's multiple Comparisons test	Mean diff.	95.00% CI of diff.	Below threshold?	Summary	Adjusted P Value			
3 mo. old vs. LV	-4.451	-12.77 to 3.867	No	ns	0.3881	A-B		
3 mo. old vs. LC	-5.626	-13.74 to 2.491	No	ns	0.2137	A-C		
LV vs. LC	-1.175	-7.195 to 4.845	No	ns	0.8773	B-C		
Test details	Mean 1	Mean 2	Mean diff.	SE of diff.	n1	n2	q	DF
3 mo. old vs. LV	3.753	8.204	-4.451	3.321	4	10	1.895	23
3 mo. old vs. LC	3.753	9.379	-5.626	3.241	4	12	2.455	23
LV vs. LC	8.204	9.379	-1.175	2.404	10	12	0.6913	23
Compact letter display								
LC	A							
LV	A							

3 mo. old	A							

Table Analyzed	D90 cystic females %macs that are cyst localized				
Data sets analyzed	A-C				
Distribution assumption	Normal (Gaussian)				
ANOVA summary					
F	4.831				
P value	0.0164				
P value summary	*				
Significant diff. among means (P < 0.05)?	Yes				
R squared	0.2709				
Brown-Forsythe test					
F (DFn, DFd)	4.097 (2, 26)				
P value	0.0284				
P value summary	*				
Are SDs significantly different (P < 0.05)?	Yes				
Bartlett's test					
Bartlett's statistic (corrected)	8.106				
P value	0.0174				
P value summary	*				
Are SDs significantly different (P < 0.05)?	Yes				
ANOVA table	SS	DF	MS	F (DFn, DFd)	P value
Treatment (between columns)	1296	2	648.2	F (2, 26) = 4.831	P=0.0164
Residual (within columns)	3489	26	134.2		
Total	4785	28			
Data summary					
Number of treatments (columns)	3				
Number of values (total)	29				

Number of families	1							
Number of comparisons per family	3							
Alpha	0.05							
Tukey's multiple comparisons test	Mean diff.	95.00% CI of diff.	Below threshold?	Summary	Adjusted P Value			
3 mo. old vs. LV	-14.57	-28.75 to -0.3812	Yes	*	0.0433	A-B		
3 mo. old vs. LC	-0.8162	-14.51 to 12.87	No	ns	0.9880	A-C		
LV vs. LC	13.75	1.425 to 26.08	Yes	*	0.0265	B-C		
Test details	Mean 1	Mean 2	Mean diff.	SE of diff.	n1	n2	q	DF
3 mo. old vs. LV	9.296	23.86	-14.57	5.709	7	10	3.609	26
3 mo. old vs. LC	9.296	10.11	-0.8162	5.509	7	12	0.2095	26
LV vs. LC	23.86	10.11	13.75	4.960	10	12	3.921	26
Compact letter display								
LV	A							
LC	B							

3 mo. old	B								

Supplemental Table 21. Welch's t-tests for Figure 3A-C.

Table Analyzed	D90 Males 2KW/BW only cystic
Column B	LC
vs.	vs.
Column A	LV
Unpaired t test	
P value	0.4402
P value summary	ns
Significantly different (P < 0.05)?	No
One- or two-tailed P value?	Two-tailed
t, df	t=0.7893, df=18
How large is the effect?	
Mean of column A	1.831
Mean of column B	1.968
Difference between means (B - A) ± SEM	0.1377 ± 0.1745
95% confidence interval	-0.2289 to 0.5044
R squared (eta squared)	0.03346
F test to compare variances	
F, DFn, Dfd	1.814, 8, 10
P value	0.3723
P value summary	ns
Significantly different (P < 0.05)?	No
Data analyzed	
Sample size, column A	11
Sample size, column B	9

Table Analyzed	D90 Males cystic index only cystic
Column B	LC
vs.	vs.
Column A	LV
Unpaired t test	
P value	0.8410
P value summary	ns
Significantly different (P < 0.05)?	No
One- or two-tailed P value?	Two-tailed
t, df	t=0.2038, df=17
How big is the difference?	
Mean of column A	10.86
Mean of column B	11.43
Difference between means (B - A) ± SEM	0.5775 ± 2.834
95% confidence interval	-5.402 to 6.557
R squared (eta squared)	0.002436
F test to compare variances	
F, DFn, Dfd	1.056, 9, 8
P value	0.9496
P value summary	ns
Significantly different (P < 0.05)?	No
Data analyzed	

Sample size, column A	10
Sample size, column B	9

Table Analyzed	D90 Males cyst count only cystic
Column B	LC
vs.	vs.
Column A	LV
Unpaired t test	
P value	0.3745
P value summary	ns
Significantly different (P < 0.05)?	No
One- or two-tailed P value?	Two-tailed
t, df	t=0.9121, df=17
How big is the difference?	
Mean of column A	1053
Mean of column B	872.2
Difference between means (B - A) ± SEM	-181.0 ± 198.4
95% confidence interval	-599.6 to 237.6
R squared (eta squared)	0.04666
F test to compare variances	
F, DFn, Dfd	3.109, 9, 8
P value	0.1249
P value summary	ns
Significantly different (P < 0.05)?	No
Data analyzed	
Sample size, column A	10
Sample size, column B	9

Table Analyzed	D90 Males avg cyst size only cystic
Column B	LC
vs.	vs.
Column A	LV
Unpaired t test	
P value	0.3535
P value summary	ns
Significantly different (P < 0.05)?	No
One- or two-tailed P value?	Two-tailed
t, df	t=0.9540, df=17
How big is the difference?	
Mean of column A	1280
Mean of column B	1515
Difference between means (B - A) ± SEM	234.9 ± 246.2
95% confidence interval	-284.6 to 754.3
R squared (eta squared)	0.05081
F test to compare variances	
F, DFn, Dfd	1.258, 8, 9
P value	0.7350
P value summary	ns

Significantly different (P < 0.05)?	No
Data analyzed	
Sample size, column A	10
Sample size, column B	9

Table Analyzed	D90 Females 2KW/BW cystic only
Column B	LC
vs.	vs.
Column A	LV
Unpaired t test	
P value	0.0147
P value summary	*
Significantly different (P < 0.05)?	Yes
One- or two-tailed P value?	Two-tailed
t, df	t=2.811, df=13
How large is the effect?	
Mean of column A	1.303
Mean of column B	1.582
Difference between means (B - A) ± SEM	0.2786 ± 0.09911
95% confidence interval	0.06451 to 0.4927
R squared (eta squared)	0.3781
F test to compare variances	
F, DFn, Dfd	6.189, 9, 4
P value	0.0948
P value summary	ns
Significantly different (P < 0.05)?	No
Data analyzed	
Sample size, column A	5
Sample size, column B	10

Table Analyzed	D90 Females cystic index only cystic
Column B	LC
vs.	vs.
Column A	LV
Unpaired t test	
P value	0.0041
P value summary	**
Significantly different (P < 0.05)?	Yes
One- or two-tailed P value?	Two-tailed
t, df	t=3.479, df=13
How large is the effect?	
Mean of column A	5.165
Mean of column B	9.891
Difference between means (B - A) ± SEM	4.725 ± 1.358
95% confidence interval	1.791 to 7.660
R squared (eta squared)	0.4821
F test to compare variances	
F, DFn, Dfd	1.370, 9, 4

P value	0.8128
P value summary	ns
Significantly different (P < 0.05)?	No
Data analyzed	
Sample size, column A	5
Sample size, column B	10

Table Analyzed	D90 Females cyst count only cystic
Column B	LC
vs.	vs.
Column A	LV
Unpaired t test	
P value	0.0879
P value summary	ns
Significantly different (P < 0.05)?	No
One- or two-tailed P value?	Two-tailed
t, df	t=1.845, df=13
How big is the difference?	
Mean of column A	640.0
Mean of column B	1044
Difference between means (B - A) ± SEM	404.2 ± 219.0
95% confidence interval	-68.97 to 877.4
R squared (eta squared)	0.2076
F test to compare variances	
F, DFn, Dfd	1.725, 9, 4
P value	0.6297
P value summary	ns
Significantly different (P < 0.05)?	No
Data analyzed	
Sample size, column A	5
Sample size, column B	10

Table Analyzed	D90 Females avg cyst size only cystic
Column B	LC
vs.	vs.
Column A	LV
Unpaired t test	
P value	0.1122
P value summary	ns
Significantly different (P < 0.05)?	No
One- or two-tailed P value?	Two-tailed
t, df	t=1.704, df=13
How big is the difference?	
Mean of column A	656.7
Mean of column B	947.4
Difference between means (B - A) ± SEM	290.7 ± 170.6
95% confidence interval	-77.90 to 659.3
R squared (eta squared)	0.1825

F test to compare variances	
F, DFn, Dfd	3.355, 9, 4
P value	0.2559
P value summary	ns
Significantly different (P < 0.05)?	No
Data analyzed	
Sample size, column A	5
Sample size, column B	10

Supplemental Table 22. Welch's t-tests for Figure 3D-F.

Table Analyzed	D90 Males picro quant
Column B	LC
vs.	vs.
Column A	LV
Unpaired t test with Welch's correction	
P value	0.3089
P value summary	ns
Significantly different (P < 0.05)?	No
One- or two-tailed P value?	Two-tailed
Welch-corrected t, df	t=1.052, df=15.37
How large is the effect?	
Mean of column A	3.285
Mean of column B	3.705
Difference between means (B - A) ± SEM	0.4204 ± 0.3995
95% confidence interval	-0.4292 to 1.270
R squared (eta squared)	0.06720
F test to compare variances	
F, DFn, Dfd	1.518, 8, 10
P value	0.5269
P value summary	ns
Significantly different (P < 0.05)?	No
Data analyzed	
Sample size, column A	11
Sample size, column B	9

Table Analyzed	D90 Males BUN quant
Column B	LC
vs.	vs.
Column A	LV
Unpaired t test with Welch's correction	
P value	0.1057
P value summary	ns
Significantly different (P < 0.05)?	No
One- or two-tailed P value?	Two-tailed
Welch-corrected t, df	t=1.735, df=13.37
How large is the effect?	
Mean of column A	18.27
Mean of column B	20.38
Difference between means (B - A) ± SEM	2.111 ± 1.217
95% confidence interval	-0.5105 to 4.733
R squared (eta squared)	0.1838
F test to compare variances	
F, DFn, Dfd	7.480, 10, 7
P value	0.0140
P value summary	*
Significantly different (P < 0.05)?	Yes
Data analyzed	

Sample size, column A	11
Sample size, column B	8

Table Analyzed	D90 Females picro quant
Column B	LC
vs.	vs.
Column A	LV
Unpaired t test with Welch's correction	
P value	0.0274
P value summary	*
Significantly different (P < 0.05)?	Yes
One- or two-tailed P value?	Two-tailed
Welch-corrected t, df	t=2.513, df=11.86
How large is the effect?	
Mean of column A	2.025
Mean of column B	3.097
Difference between means (B - A) ± SEM	1.072 ± 0.4267
95% confidence interval	0.1415 to 2.003
R squared (eta squared)	0.3476
F test to compare variances	
F, DFn, Dfd	10.16, 9, 4
P value	0.0394
P value summary	*
Significantly different (P < 0.05)?	Yes
Data analyzed	
Sample size, column A	5
Sample size, column B	10

Table Analyzed	D90 Females BUN quant
Column B	LC
vs.	vs.
Column A	LV
Unpaired t test with Welch's correction	
P value	0.0125
P value summary	*
Significantly different (P < 0.05)?	Yes
One- or two-tailed P value?	Two-tailed
Welch-corrected t, df	t=3.402, df=6.599
How large is the effect?	
Mean of column A	15.56
Mean of column B	21.09
Difference between means (B - A) ± SEM	5.529 ± 1.625
95% confidence interval	1.638 to 9.420
R squared (eta squared)	0.6369
F test to compare variances	
F, DFn, Dfd	1.603, 4, 9
P value	0.5104
P value summary	ns
Significantly different (P < 0.05)?	No

Data analyzed	
Sample size, column A	5
Sample size, column B	10

Supplemental Table 23. Primary antibodies used for flow cytometry.

Target antigen	Fluorophore	Vendor or Source	Catalog #	Clone	Lot #	Working Concentration
CD64	PerCP-Cy5.5	BioLegend	139308	X54-5/7.1	B384717	1:200
CD115	APC	BioLegend	135510	AFS98	B356285	1:200
CD11c	PE-Cy5	BioLegend	117316	N418	B429521	1:200
CD11b	BV650	BioLegend	101239	M1/70	B447001	1:200
Ly-6C	BV711	BioLegend	128037	HK1.4	B455499	1:200
Ly-6G	APC-Cy7	BioLegend	127624	1A8	B446227	1:100
I-A/I-E (MHC class II)	AF700	BioLegend	107622	M5/114.15.2	B448965	1:100
CD45	BV786	BD Pharmigen	557659	30-F11	4269071	1:200
CD43	BV605	BD OptiBuild	747726	S7	5086722	1:200
F4/80	eFluor 450	Invitrogen	48-4801-82	BM8	2964845	1:200
Live/Dead	Zombie Aqua	Invitrogen	L34966	N/A	2535918	1:500

Supplemental Table 24. List of flow cytometry, staining, and immunofluorescence reagents.

Reagent	Vendor	Cat. No.	Concentration or dilution used
Bovine Serum Albumin (BSA)	Fisher Scientific	BP1600-100	1% w/v
Dulbecco's Phosphate Buffered Saline (DPBS)	Corning	21-030-CV	
RPMI 1640	Gibco	32-404-014	
Collagenase type I	Sigma-Aldrich	C0130	1 mg/mL
Collagenase type IV	Sigma-Aldrich	C5138	1 mg/mL
Deoxyribonuclease I (DNase I)	Sigma-Aldrich	D5025-150KU	100 U/mL
70 µm cell strainers	Falcon	08-771-2	
ACK red blood cell lysis buffer	Quality Biological	50-751-7469	
Fc-blocker (anti-mouse CD16/CD32)	Fisher Scientific	50-152-8494	1:200
5% formalin	Fisher Scientific	SF100-4	
EDTA-coated blood collection tubes	BD Vacutainer	02-689-4	
Modified Harris Hematoxylin solution	Epredia	72704	
Eosin solution	Sigma-Aldrich	HT110132-1L	
Picrosirius Red stain kit	Polysciences	24901-500	
Blood urea nitrogen kit	Bioassay Systems	50-107-8333	
30% D-sucrose solution	Fisher Scientific	BP220-212	
O.C.T. medium	Fisher Scientific	23-730-571	
Triton X-100	Fisher Scientific	BP151-500	0.1% in 1X PBS
Donkey Serum	Sigma-Aldrich	D9663	1% v/v
Sodium Azide	Fischer Scientific	S2271-25	0.02%
Rat anti-mouse F4/80	Thermo Fisher	No. 14-4801-82; Clone: BM8	1:200
AF647 donkey anti-rat	Jackson ImmunoResearch	712-606-153, Lot: 148033	1:500
Hoescht 33258	Invitrogen	H3569	1:1000
Immu-Mount	Epredia	9990402	
Mounting coverslips	Fisher Scientific	12-541-056	

Supplemental Table 25. qRT-PCR primer sequences.

Gene and primer	Forward	Reverse
Ccl2 (Mm00441242_m1)	TAAAAACCTGGATCGGAACCAAA	GCATTAGCTTCAGATTTACGGGT
Cx3cl1 (Mm00436454_m1)	CTGGCCGCGTTCTTCCATT	GCACATGATTTCGCATTTCGT
Csf1 (Mm00432686_m1)	GACCTGCTCCACTTCTCCAG	GGGTTCAGACCAAGCGAGAAG

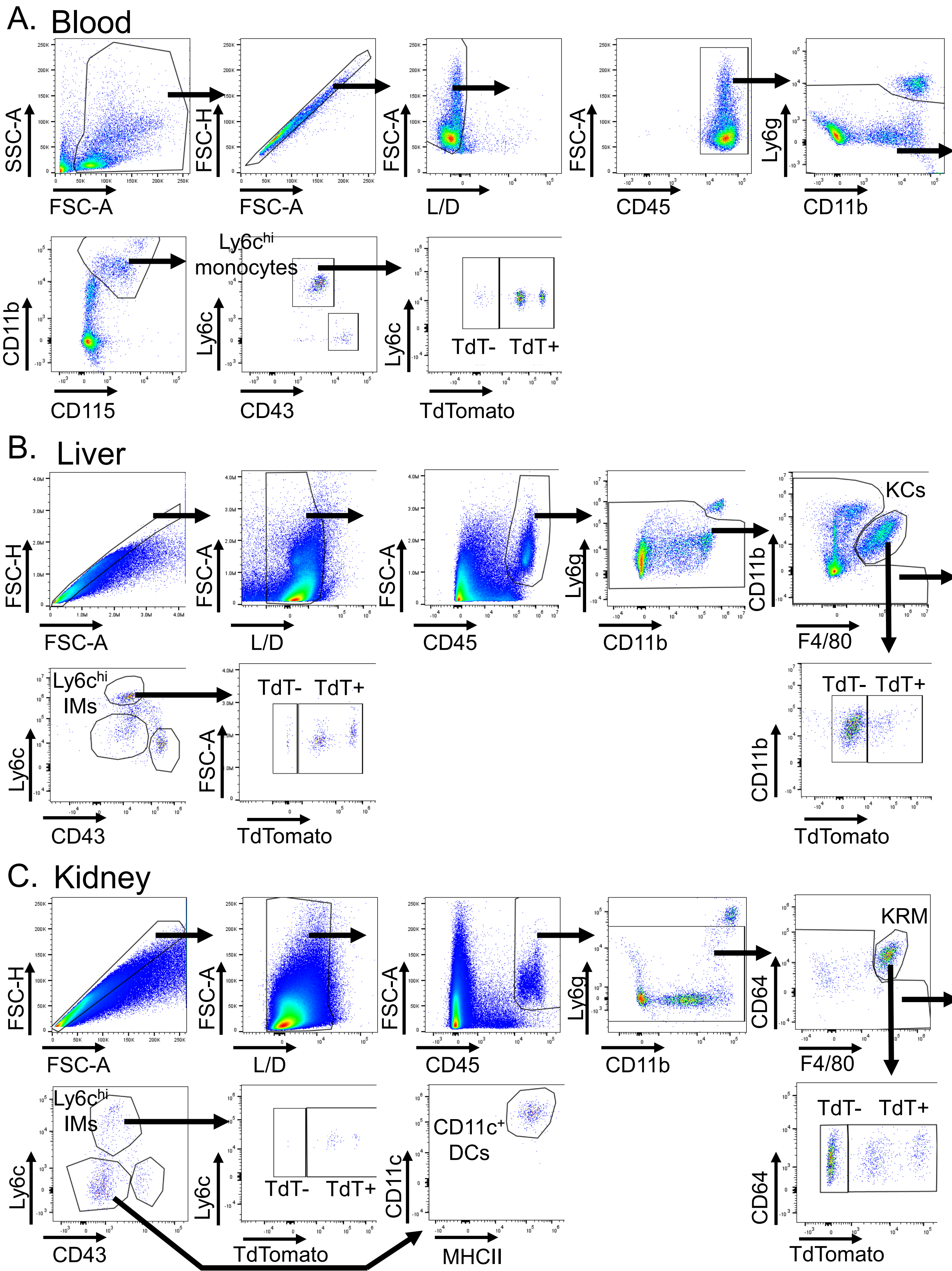
A. Blood
SSC-A
FSC-A
FSC-H
FSC-A
FSC-A
L/D
FSC-A
CD45
Ly6g
CD11b
CD11b
CD115
Ly6c^hi monocytes
Ly6c
CD43
Ly6c
TdT-
TdT+
TdTomato
B. Liver
FSC-H
FSC-A
FSC-A
L/D
FSC-A
CD45
Ly6g
CD11b
KCs
CD11b
F4/80
Ly6c^hi IMs
Ly6c
CD43
TdT-
TdT+
FSC-A
TdTomato
TdT-
TdT+
CD11b
TdTomato
C. Kidney
FSC-H
FSC-A
FSC-A
L/D
FSC-A
CD45
Ly6g
CD11b
KRM
CD64
F4/80
Ly6c^hi IMs
Ly6c
CD43
Ly6c
TdT-
TdT+
TdTomato
CD11c+ DCs
CD11c
MHCII
TdT-
TdT+
CD64
TdTomato

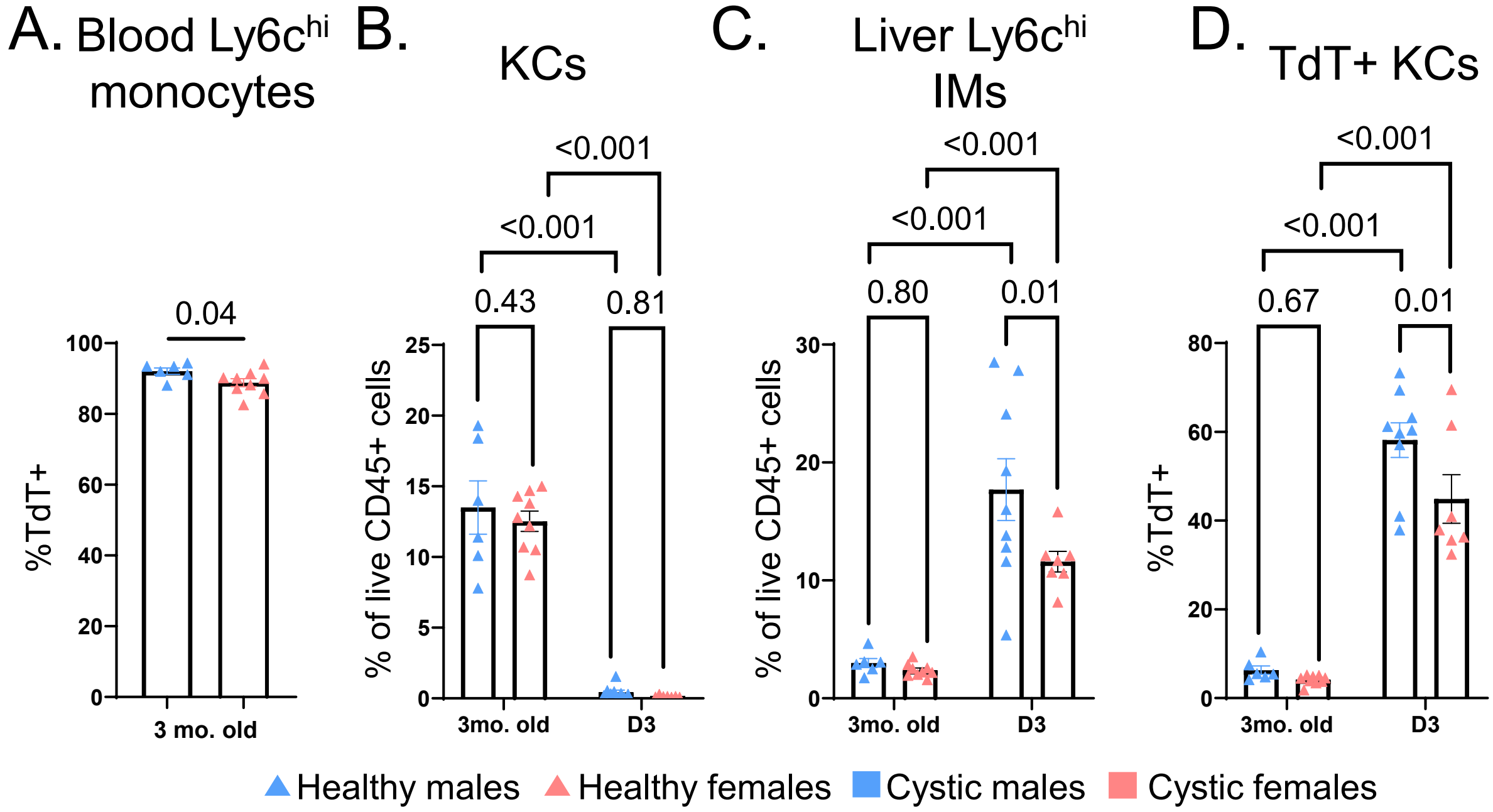
A. Blood Ly6c^hi monocytes
0.04
%TdT+
100
80
60
40
20
0
3 mo. old
B. KCs
<0.001
<0.001
0.43
0.81
% of live CD45+ cells
25
20
15
10
5
0
3mo. old
D3
C. Liver Ly6c^hi IMs
<0.001
<0.001
0.80
0.01
% of live CD45+ cells
30
20
10
0
3mo. old
D3
D. TdT+ KCs
<0.001
<0.001
0.67
0.01
%TdT+
80
60
40
20
0
3mo. old
D3
Healthy males
Healthy females
Cystic males
Cystic females

A.

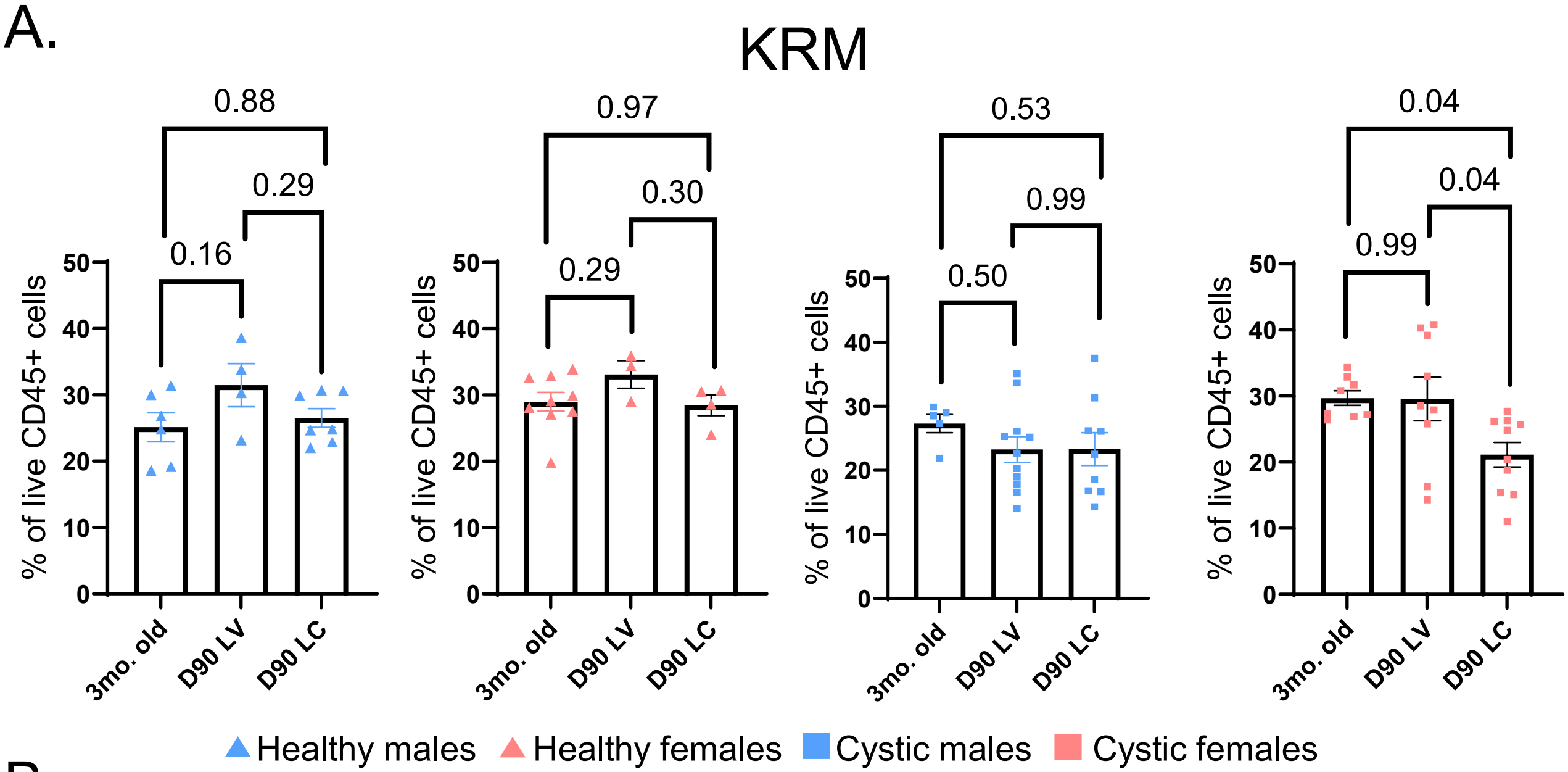

KRM
0.88
0.29
0.16
0.97
0.30
0.29
0.53
0.99
0.50
0.04
0.04
0.99
% of live CD45+ cells
3mo. old
D90 LV
D90 LC
Healthy males
Healthy females
Cystic males
Cystic females

B.

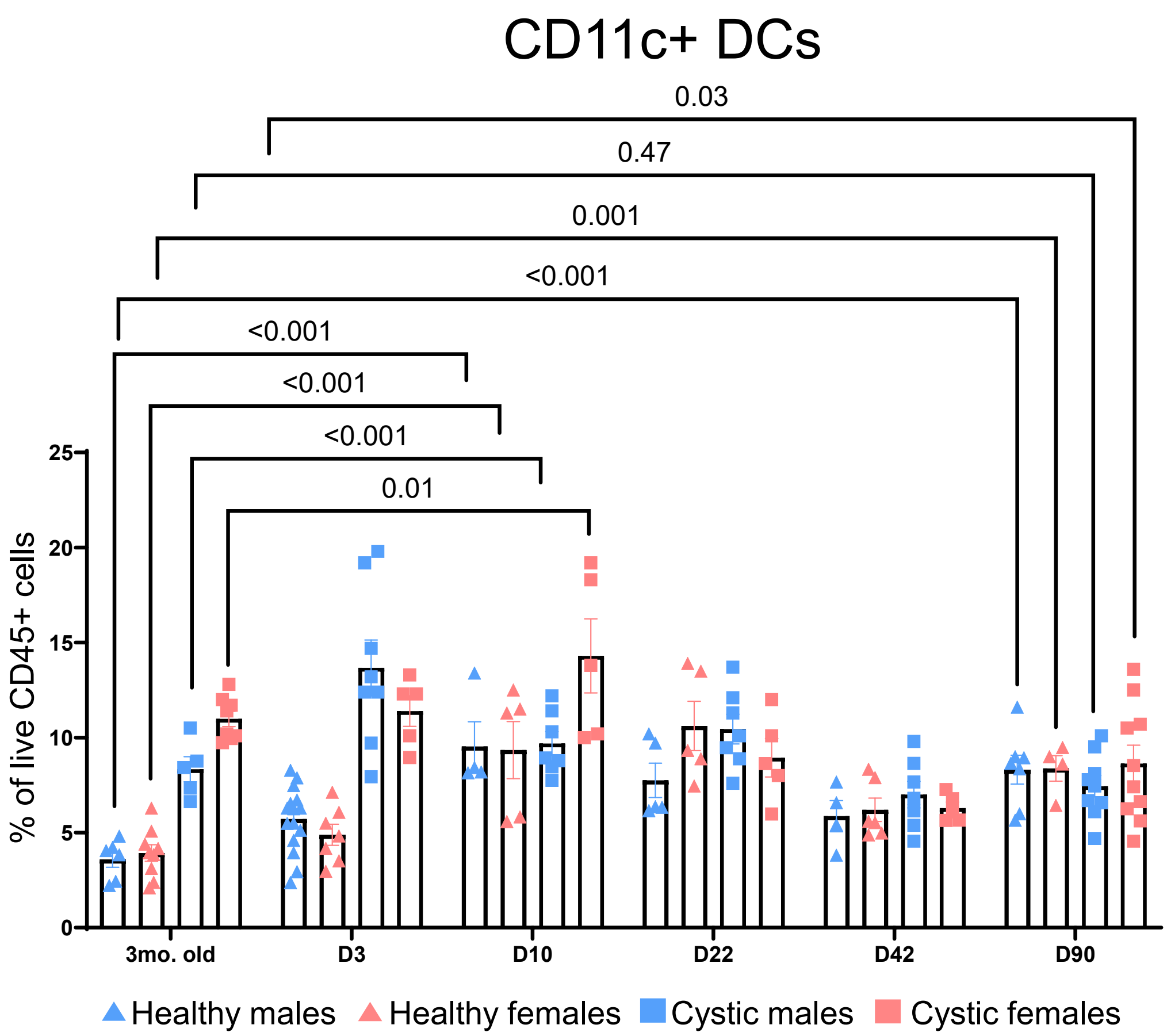

CD11c+ DCs
0.03
0.47
0.001
<0.001
<0.001
<0.001
<0.001
0.01
% of live CD45+ cells
3mo. old
D3
D10
D22
D42
D90
Healthy males
Healthy females
Cystic males
Cystic females

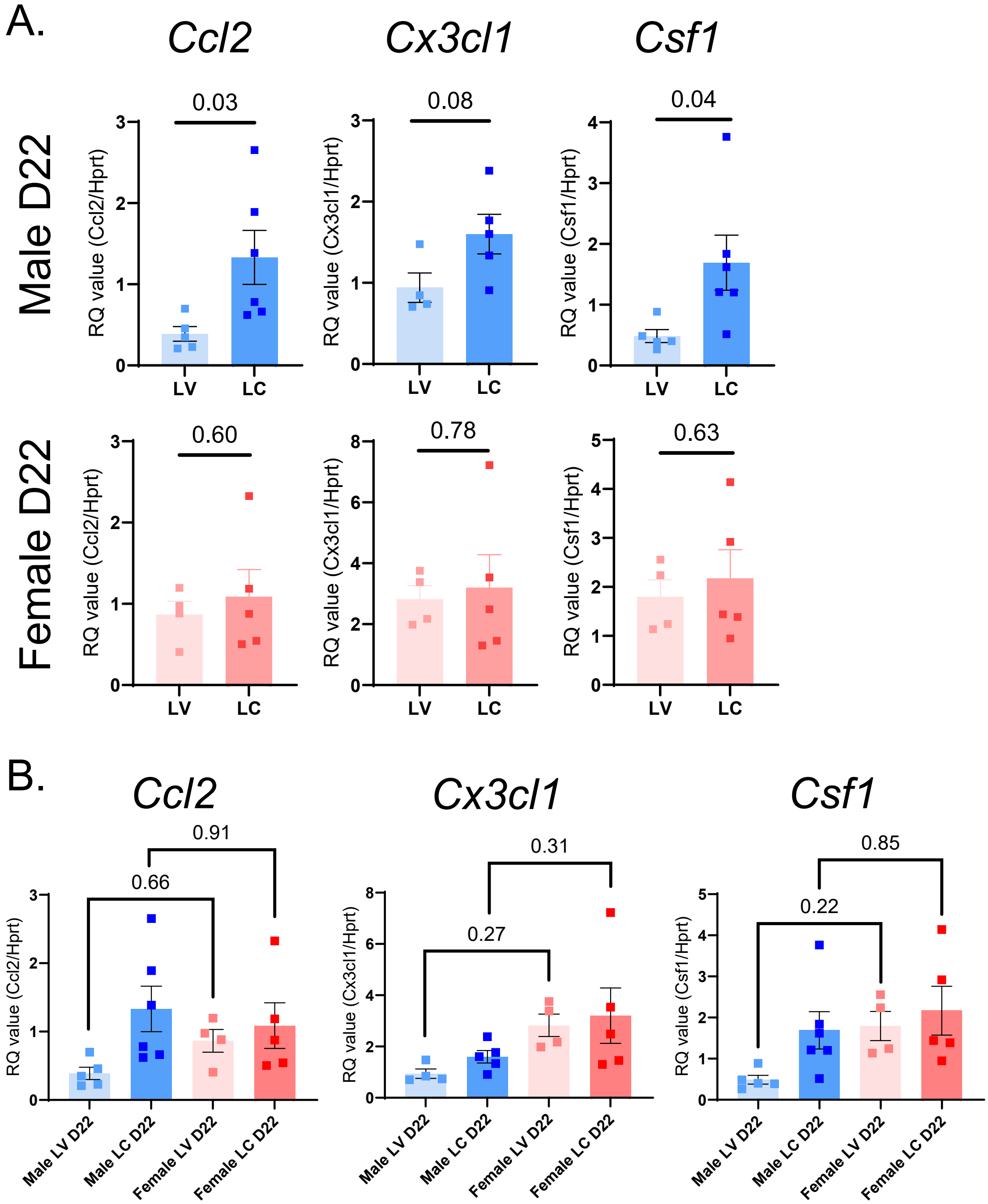

A.
Ccl2
Cx3cl1
Csf1
Male D22
Female D22
0.03
0.08
0.04
0.60
0.78
0.63
RQ value (Ccl2/Hprt)
RQ value (Cx3cl1/Hprt)
RQ value (Csf1/Hprt)
LV
LC
B.
Ccl2
Cx3cl1
Csf1
0.91
0.66
0.31
0.27
0.85
0.22
Male LV D22
Male LC D22
Female LV D22
Female LC D22
